# Impact of the number of mutations in survival and response outcomes to hypomethylating agents in patients with myelodysplastic syndromes or myelodysplastic/myeloproliferative neoplasms

**DOI:** 10.18632/oncotarget.23882

**Published:** 2018-01-03

**Authors:** Guillermo Montalban-Bravo, Koichi Takahashi, Keyur Patel, Feng Wang, Song Xingzhi, Graciela M. Nogueras, Xuelin Huang, Ana Alfonso Pierola, Elias Jabbour, Simona Colla, Irene Gañan-Gomez, Gautham Borthakur, Naval Daver, Zeev Estrov, Tapan Kadia, Naveen Pemmaraju, Farhad Ravandi, Carlos Bueso-Ramos, Ali Chamseddine, Marina Konopleva, Jianhua Zhang, Hagop Kantarjian, Andrew Futreal, Guillermo Garcia-Manero

**Affiliations:** ^1^ Department of Leukemia, The University of Texas MD Anderson Cancer Center, Houston, Texas, USA; ^2^ Department of Genomic Medicine, The University of Texas MD Anderson Cancer Center, Houston, Texas, USA; ^3^ Department of Hematopathology, The University of Texas MD Anderson Cancer Center, Houston, Texas, USA; ^4^ Applied Cancer Science Institute, The University of Texas MD Anderson Cancer Center, Houston, Texas, USA; ^5^ Department of Biostatistics, The University of Texas MD Anderson Cancer Center, Houston, Texas, USA

**Keywords:** myelodysplastic syndromes, chronic myelomonocytic leukemia, response, prognosis, mutations

## Abstract

The prognostic and predictive value of sequencing analysis in myelodysplastic syndromes (MDS) has not been fully integrated into clinical practice. We performed whole exome sequencing (WES) of bone marrow samples from 83 patients with MDS and 31 with MDS/MPN identifying 218 driver mutations in 31 genes in 98 (86%) patients. A total of 65 (57%) patients received therapy with hypomethylating agents. By univariate analysis, mutations in *BCOR, STAG2, TP53* and *SF3B1* significantly influenced survival. Increased number of mutations (≥ 3), but not clonal heterogeneity, predicted for shorter survival and LFS. Presence of 3 or more mutations also predicted for lower likelihood of response (26 vs 50%, *p* = 0.055), and shorter response duration (3.6 vs 26.5 months, *p* = 0.022). By multivariate analysis, *TP53* mutations (HR 3.1, CI 1.3–7.5, *p* = 0.011) and number of mutations (≥ 3) (HR 2.5, CI 1.3–4.8, *p* = 0.005) predicted for shorter survival. A novel prognostic model integrating this mutation data with IPSS-R separated patients into three categories with median survival of not reached, 29 months and 12 months respectively (*p* < 0.001) and increased stratification potential, compared to IPSS-R, in patients with high/very-high IPSS-R. This model was validated in a separate cohort of 413 patients with untreated MDS. Although the use of WES did not provide significant more information than that obtained with targeted sequencing, our findings indicate that increased number of mutations is an independent prognostic factor in MDS and that mutation data can add value to clinical prognostic models.

## INTRODUCTION

Clinical outcomes of patients with myelodysplastic syndromes (MDS) and myelodysplastic/myeloproliferative neoplasms (MDS/MPN), including chronic myelomonocytic leukemia (CMML), are very heterogeneous. Development of several prognostic risk models such as the IPSS [1] and IPSS-R [2] has allowed the stratification of patients with MDS to predict clinical outcomes and select therapy. These clasifications are based mainly in cytogenetic information, blast counts and degree of cytopenias.

Next generation sequencing techniques have allowed identification of multiple somatic mutations in relevant functional pathways [3–6] and have improved our understanding of the etiology of the disease, but have not been incorporated in clinical practice yet.

Although there is increasing data of the potential prognostic impact of single gene somatic mutations, there is still contradictory results among different studies [3, 7–10]. Several studies have evaluated the integration of mutation data with other patient characteristics to develop combined clinical and molecular classifications. In such studies, incorporation of sequencing data into current prognostic systems yielded improved stratification potential [4, 11]. However, new validated clinical-molecular prognostic risk systems aimed at substituting the current IPSS-R have not been developed. Although several studies have described the potential use of genomic abnormalities as predictors of response to therapy with hypomethylating agents [12–15], there are no validated biomarkers for response. Additionally, although the number of cytogenetic abnormalities (i.e complex karyotype) is a well-known prognostic factor in MDS [16–18], there is less data evaluating the value of the number of mutations in the prognosis of MDS and MDS/MPN (5). To improve our ability to predict patient outcomes, validated integration of this new mutation data into current prognostic models is needed.

To further explore the potential incorporation of mutation data into clinical practice, we performed whole exome sequencing (WES) of bone marrow samples from 114 previously untreated patients, 83 with MDS and 31 with MDS/MPN (including 26 patients with CMML), to evaluate the impact of each given mutation, mutation clonality and number of mutations in survival and treatment outcomes. By using this data, we could integrate molecular variables into existing scoring systems improving our ability to stratify patient risk and prognosis and validated these results in an independent cohort of 413 patients with MDS and MDS/MPN.

## RESULTS

### Patient characteristics

Clinical characteristics of the 114 patients with MDS are listed in Table 1. The median age was 66 years (range: 19-85). Ten (9%) patients had MDS with single lineage dysplasia (MDS-SLD), 7 (6%) MDS with ringed sideroblasts (MDS-RS), 25 (22%) MDS with multilineage dysplasia (MDS-MLD), 37 (33%) MDS with excess blasts (MDS-EB), 4 (4%) MDS unclassifiable (MDS-U), 5 (4%) myelodysplastic/myeloproliferative neoplasm (MDS/MPN). In addition, 26 (23%) patients had chronic myelomonocytic leukemia (CMML). One-hundred and ten (96%) patients had evaluable cytogenetics with 55 (48%) patients having normal karyotype and 18 patients having (16%) complex karyotype. A total of 65 (57%) patients were treated with frontline hypomethylating agents, 16 (24.6%) and 20 (30.7%) with azacitidine or decitabine, respectively, and 29 (44.6%) with either guadecitabine (SGI-110) or combination therapies within a clinical trial.

**Table 1:** Patient characteristics

Variable	Number [Range]/(%)
Age	66 [19–85]
Female	32 (28)
WBC (×10^9^/L)	4.3 [1.0–70.1]
ANC (×10^9^/L)	2.4 [0.1–42.5]
Hb (g/dL)	10.1 [6–15.8]
PLT (×10^9^/L)	89 [11–1033]
Bone marrow blasts (%)	5 [0–20]
WHO Classification	
MDS-SLD	10 (9)
MDS-RS	7 (6)
MDS-MLD	25 (22)
MDS-EB	37 (33)
MDS-U	4 (4)
MDS/MPN-U	5 (4)
CMML	26 (23)
Cytogenetic Abnormalities^*^	
Normal	55 (48)
-Y	5 (5)
del(3)/inv(3)	6 (6)
del(5q)	11 (10)
del(7q)/-7	14 (12)
+8	11(10)
del(11q)	2 (2)
del(12q)	6 (6)
del(17p)	6 (6)
i(17q)	2 (2)
+19	2 (2)
del(20q)	10 (9)
Insufficient metaphases	4 (4)
Complex Cytogenetics	18 (16)
IPSS	
Low	22 (21)
Int-1	45 (43)
Int-2	29 (38)
High	9 (9)
IPSS-R	
Very low	11 (10)
Low	23 (20)
Intermediate	33 (29)
High	28 (25)
Very high	13 (11)
Unknown	6 (5)
Therapy Related	19 (17)
Hypomethylating therapy	61 (54)

^*^ Relation of cytogenetic abnormalities identified in the patient population. Some cases presented more than one. Displayed percentages correspond to prevalence of cytogenetic abnormality isolated or as part of a more complex karyotype.

MDS: Myelodysplastic syndrome. MDS-SLD: MDS with single lineage dysplasia. MDS-RS: MDS with ringed sideroblasts. MDS-MLD: MDS with multilineage dysplasia. MDS-EB: MDS with excess blasts. MDS-U: MDS unclassifiable. MDS/MPN-U: unclassifiable myelodysplastic/myeloproliferative neoplasm. CMML: Chronic myelomonocytic leukemia.

### Landscape of driver mutations

**Figure 1:**
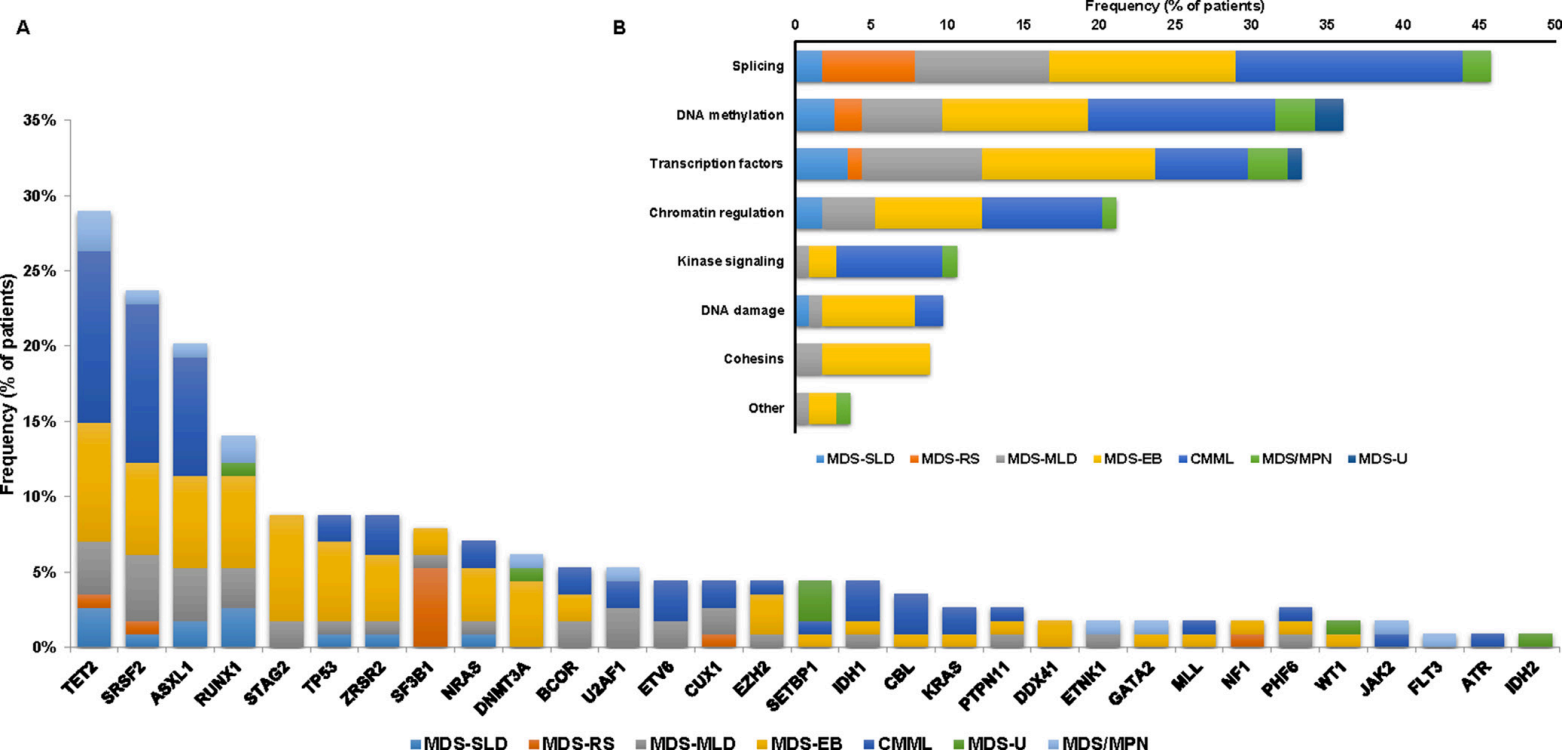
Mutational landscape of the studied MDS cohort (**A**) Distribution and frequency of identified mutations by WHO Classification. The Y axis includes the percentage of patients harboring the specified mutation. Stacked columns display prevalence of each given mutation by WHO classification. (**B**) Distribution of mutations by pathway within the 114 MDS patients.

Exome sequencing detected a total of 218 high-confidence mutations in 31 genes in 98 patients (86%) (Supplementary Table 1, Supplementary Material). We did not detect any mutations in 16 (14%) patients. The median number of mutations detected in each patient was 2 (range: 0-7) (Supplementary Figure 1, Supplementary Material). Distribution of mutations by WHO classification is shown in Figure 1A. Comparison of the distribution of mutations with previous studies [4, 5] is shown in Supplementary Figure 2 (Supplementary Material). Similar frequencies of the identified mutations where observed, with the exception of a lower frequency of *SF3B1* and *DNMT3A* mutations. The most frequently detected mutations included *TET2*, *SRSF2*, *ASXL1* and *RUNX1* in > 10% patients. We found 2 patients, one with MDS and one with MDS/MPN, with *ETNK1 N244S* mutation, which has been previously described in atypical CML, CMML and systemic mastocytosis but not in MDS [19–21].

Mutated genes were categorized among several functional pathways relevant in MDS (Figure 1B). The median number of altered pathways was 2 (range 0-6) with 43 (38%) cases having mutations in one pathway, 24 (21%) in two, 20 (18%) in three, 10 (9%) in four and 1 (1%) in six. A total of 16 cases had more than one detectable mutation in a same functional pathway including 5 cases (4%) with mutations within two DNA methylation genes, 4 (4%) with mutations in two chromatin regulation genes and 7 (6%) with mutations in two transcriptional regulators.

### Association between genomic abnormalities

**Figure 2:**
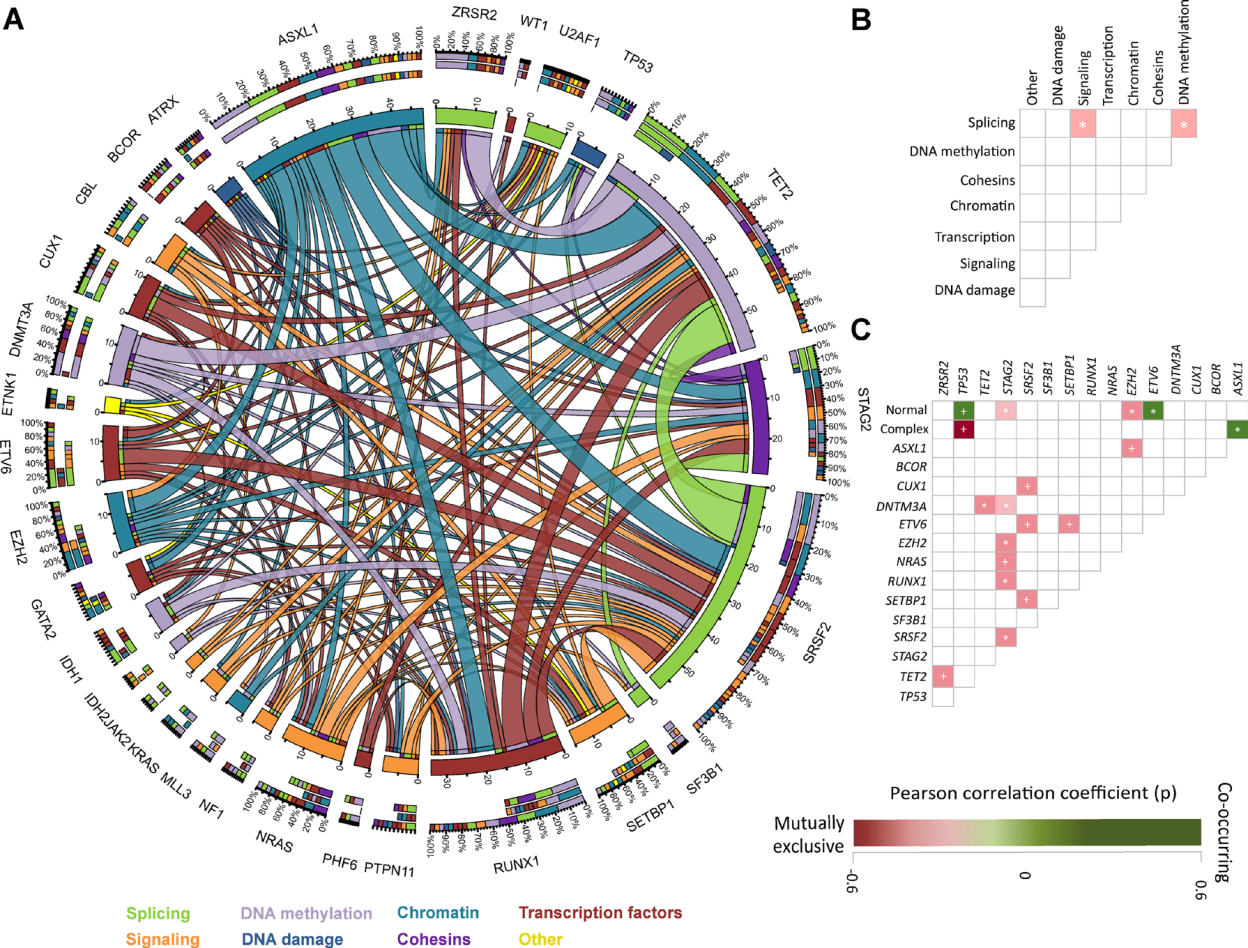
Association of mutations and pathway distribution (**A**) Circos plot including all mutation associations among the discovery cohort. Colors are determined by functional pathway of each given gene. (**B**) Patterns of association of pathway abnormalities among studied patients. Areas shaded in pink represent co-occurrence. ^*^ = *p* < 0.05. + = *p* < 0.001 (**C**) Patterns of association of mutations and karyotype among studied patients. Areas shaded in pink represent co-occurrence and those in green mutual exclusiveness. Color palette determined by Pearson´s r correlation. ^*^ = *p* < 0.05. + = *p* < 0.001.

To study whether specific mutational events tend to occur with other given mutations or cytogenetic abnormalities we evaluated patterns of occurrence of genomic alterations. Fifteen significant pairwise associations between identified mutations were observed (Figure 2A) (Supplementary Table 2, Supplementary Material). In most of these genomic pairing associations, different relevant molecular pathways were involved. Association between different mutations within a same molecular pathway was also identified. Mutations affecting splicing genes where commonly associated with mutations in genes involved in signaling and DNA methylation (Figure 2B).

Mutations in *EZH2* (0.218, *p* = 0.02), *STAG2* (r = 0.190, *p* = 0.04) and *ASXL1* (r = 0.182, *p* = 0.05) were more commonly observed in patients with normal karyotype, with mutations in *ETV6* being rare in these patients (r = –0.218, *p* = 0.02). Conversely, mutations on *TP53* were more common among patients with complex karyotype (*p* < 0.001) as previously reported [8, 22, 23]. In addition, *ASXL1* had mutual exclusiveness with complex karyotype (r = –0.221, *p* = 0.02). No significant differences in mutation distribution was observed in patients with specific isolated cytogenetic abnormalities (Figure 2C).

### Mutation clonality and clonal heterogeneity

**Figure 3:**
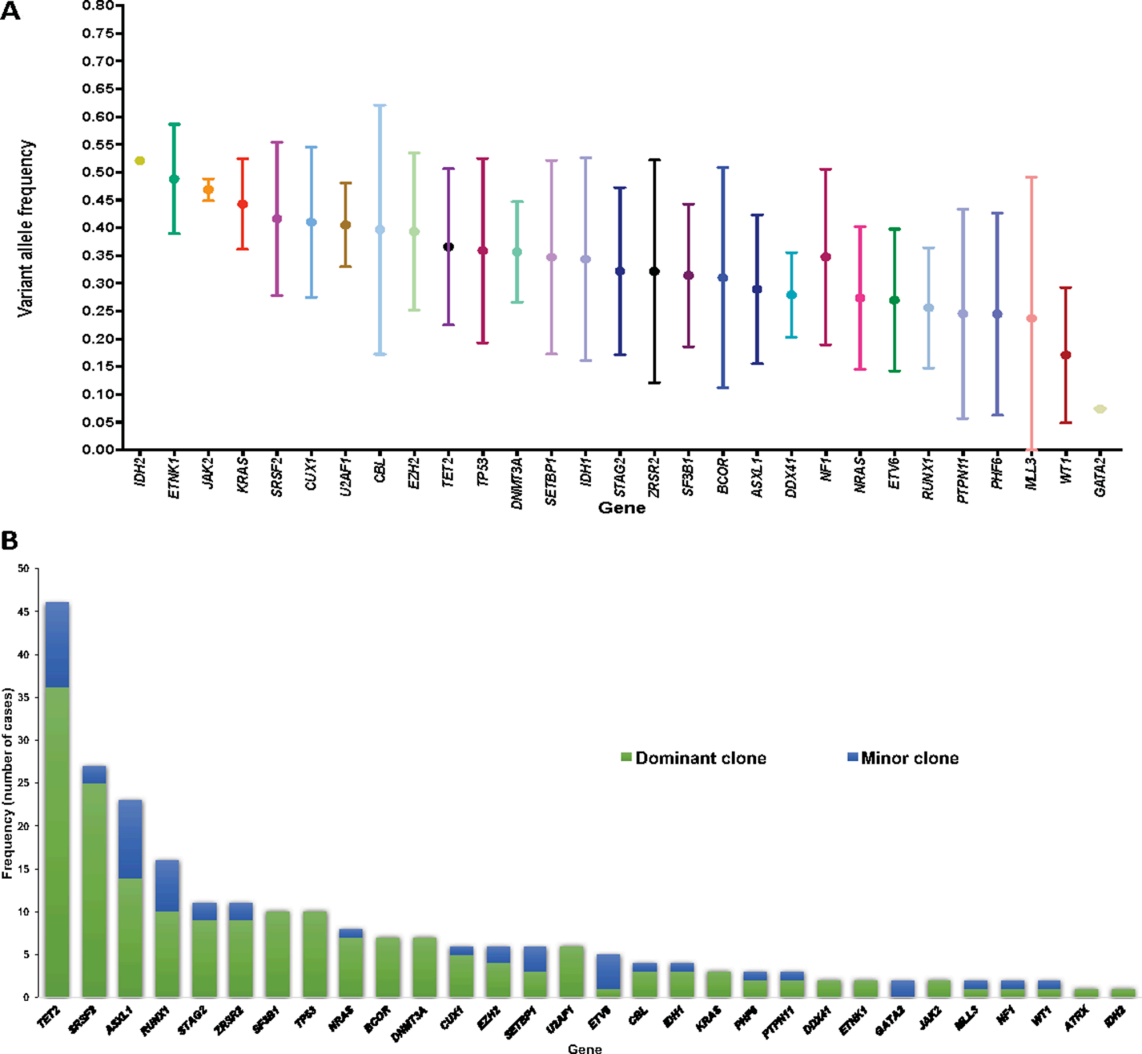
Graphical representation of clonal distribution (**A**) Mean and standard deviation of VAF of identified mutations. (**B**) Distribution and frequency of identified mutations within major or minor clones among patients with clonal heterogeneity.

Figure 3A shows allelic frequencies of detected mutations. When grouping mutations into functionally relevant pathways, mutations in DNA damage, splicing and DNA methylation pathways tended to have higher VAF (mean 0.46, 0.40 and 0.38 respectively). Among the 57 patients evaluable for clonal heterogeneity, 30 (53%) where clonally heterogeneous and carried more than one clone (Supplementary Figure 3, Supplementary Material). Among mutations detected within non-dominant clones (those with significantly smaller VAFs), *TET2* was the most common appearing as a minor clone in 10 occasions, followed by *ASXL1* in 9 and *RUNX1* in 6. Evaluation of clonal size based on VAF and clonal heterogeneity identified mutations which only appeared in dominant clones such as *SF3B1, TP53, BCOR, DNMT3A* or *U2AF1* with other mutational events being limited to minor clones (*ETV6*: 4/5, *GATA:* 2/2) (Figure 3C).

### Prognostic impact of driver mutations

The median follow-up of the cohort was 21.6 months (range 1-102 months). In univariate analysis, mutations in *BCOR* (HR 2.85, 95% CI 1.12–7.29, *p* = 0.029)*, STAG2* (HR 2.45, 95% CI 1.04–5.80, *p* = 0.041) and *TP53* (HR 5.25, 95% CI 2.37–11.63, *p* < 0.001) affected overall survival (OS) unfavorably and *SF3B1* mutation affected OS favorably (median survival NR vs 28.7 months, *p* = 0.023) (Supplementary Table 3, Supplementary Material). When evaluating leukemia-free survival (LFS), *EZH2* (HR 8.49, 95% CI 1.85–39.02, *p* = 0.006) and *TP53* mutations (HR 6.24, 1.63–23.86, *p* = 0.007) predicted for shorter LFS. Overall survival negatively correlated with increased number of mutations (*p* = 0.03, Supplementary Figure 4, Supplementary Material) with patients with 3 or more driver mutations having significantly worse outcomes (HR 1.94, 95% CI 1.10-3.41, *p* = 0.020). Similarly, mutation number was also predictive of shorter LFS (HR 1.5, 95% CI 1.06–2.12, *p* = 0.021) (Supplementary Figure 4, Supplementary Material).

Due to biological differences among MDS and MDS/MPN patients, we performed subset analysis among these two groups of patients separately (Supplementary Tables 4–7, Supplementary Material). When evaluating the 83 patients with MDS, only *STAG2* (HR 2.7, 95% CI 1.11-6.57, *p* = 0.029) and *TP53* (HR 5.19, 95% CI 2.05-13.12, *p* = 0.001) mutations impacted prognosis adversely, with *TP53* mutations also predicting for shorter LFS (HR 8.96, 95% CI 2.10-38.12, *p* = 0.003). Among patients with a diagnosis of MDS/MPN overlap syndrome, 84% of which had CMML, presence of mutations in *EZH2* (HR 28.5, 95% CI 1.78–455.65, *p* = 0.018) and *TP53* (HR 6.6, 95% CI 1.26–34.43, *p* = 0.025) as well as increased number of mutations, particularly if more than 3 (HR 3.5, 95% CI 1.09–11.19, *p* = 0.035) where associated with shorter overall survival. Presence of 4 or more mutations was associated with shorter LFS (HR 15.29, 95% CI 1.58–147.71, *p* = 0.018).

To determine whether the presence of clonal heterogeneity significantly influences the prognosis of patients with MDS, we analyzed survival outcomes based on the presence of clonal heterogeneity. Although a trend to worse OS (median OS 21.3 months vs 43.2 months, HR 1.78, 95% CI 0.98–3.18, *p* = 0.059) was observed in clonally heterogeneous patients, observed differences were not significant. No differences in LFS were observed based on the presence of clonal heterogeneity.

### Characteristics of patients with increased number of mutations

In view of the prognostic impact of mutation number we analyzed the characteristics of this group of patients. Thirty-eight patients (33%) had 3 or more mutations. No significant differences in age, IPSS or IPSS-R scores were observed compared to patients with lower number of mutations (Supplementary Table 8, Supplementary Material). Patients with MDS/MPN (mainly CMML) where more likely to have 3 or more mutations compared to patients with MDS (48% vs 28%, *p* = 0.037). Although not statistically significant, patients with 3 or more mutations did not usually have complex karyotype (8% vs 21% in patients with < 3 mutations, *p* = 0.11). There was no association between presence of 3 or more mutations and that of a normal karyotype (47% vs 57% in those with < 3 and ≥ 3 respectively, *p* = 313). Mutations in *SF3B1* and *TP53*, which carry significant prognostic impact by themselves, were not generally present in these patients (Supplementary Table 8 and Supplementary Figure 5 Supplementary Material).

### Influence on mutation profile in response to hypomethylating agents

Among the 65 patients who received therapy with hypomethylating agents, 61 (94%) where evaluable for response. The overall response rate (ORR) was 51% (31/61) including 16 (26%) complete responses. The median time to response was 2.8 months (range 0.6–12.9) and the median response duration was 5.2 months (0–34.1). To evaluate whether any of the mutational events influenced response outcomes, we studied response based on mutated gene, number of mutations and clonal size of each given mutation. To assess whether clonal size of identified mutations influenced response, specific thresholds of VAF associated with response where generated for all mutations based on AUC using ROC analysis. None of the identified mutations or VAF cutoffs where associated with response outcomes (Supplementary Table 9 and Supplementary Figure 6, Supplementary Material). Increasing number of mutations, particularly if more than 3 (26% vs 50%, OR 0.35, 95% CI 0.12–1.02, *p* = 0.055) or more than 4 (6% vs 27%, OR 0.19, 95% CI 0.04-0.98, *p* = 0.048) was associated with a lower likelihood of achieving response to therapy. Only the number of detectable mutations was significantly associated with a lower likelihood of achieving CR (OR 0.47, 95% CI 0.23-0.96, *p* = 0.039). When evaluating response duration, presence of a normal karyotype was associated with longer response duration (26 vs 5.4 months, OR 0.32, 95% CI 0.11-0.99, *p* = 0.047). Presence of a mutation in a DNA methylation regulator (3.6 vs 26.5 months, OR 2.7, 95% CI 1.01–7.19, *p* = 0.048) and presence of 3 or more mutations (3.6 vs 26.5 months, OR 3.34, 95% CI 1.19–9.36, *p* = 0.022) where associated with shorter median response duration.

Among this treated population, mutations in *TP53* (7.7 vs 15.4 months, HR 3.46, 95% CI 1.52-7.84, *p* = 0.003), *SRSF2* (7.8 vs 14.5 months, HR 2.56, 95% CI 1.09–6.01, *p* = 0.031) and increased number of mutations, particularly if 3 or more (9.6 months vs NR, HR 3.27, 95% CI 1.61-6.61, *p* = 0.001) or 4 or more (6 vs 14.5 months, HR 5.87, 95% CI 2.05-16.87, *p* = 0.001) was associated with shorter median overall survival.

### Integration of molecular data into existing prognostic scoring systems

To evaluate whether integration of identified genomic variables into current prognostic systems could improve stratification potential, we performed multivariate analysis for survival including IPSS-R category along with the prognostic mutational variables identified. IPSS-R group (Intermediate: HR 1.45, 95% CI 0.56–3.77, *p* = 0.45; High/Very High; HR 4.66, 95% CI 2.01–10.84, *p* < 0.001), *TP53* mutation (HR 3.12, 95% CI 1.3–7.49, *p* = 0.011), and ≥ 3 mutations (HR 2.51, 95% CI 1.32–4.76, *p* = 0.005) retained their adverse impact on outcome (Table 2). This data was used to develop a molecular IPSS-R (MIPSS-R) model with scoring being determined by the HR of each given variable: IPSS-R Intermediate = 0.5 points, High/Very High = 1.5 points, presence of *TP53* mutation = 1 point, presence of ≥ 3 mutations = 1 point (Table 2).

**Table 2:** Multivariate analysis for survival integrating IPSS-R variables and mutational data and assigned score in the molecular IPSS-R model

Variable	Hazard Ratio	95% CI	*p* value	Score
IPSS-R				
Intermediate	1.45	0.56–3.77	0.45	0.5
High/Very high	4.66	2.01–10.84	< 0.001	1.5
TP53	3.12	1.3–7.49	0.011	1
Mutations 3 or more	2.51	1.32–4.76	0.005	1

**Figure 4:**
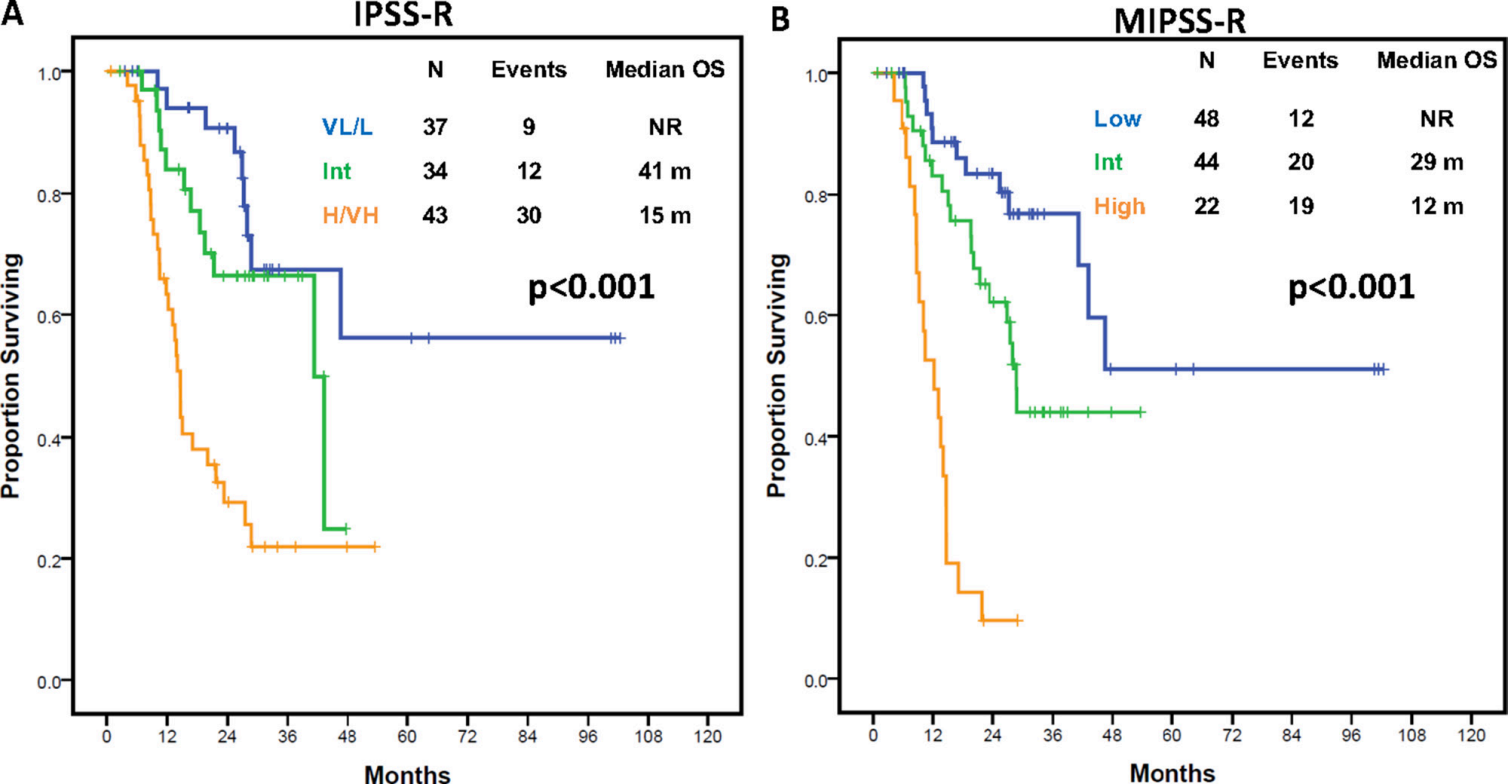
Overall survival outcomes by IPSS-R and molecular IPSS-R model in the discovery cohort (**A**) Kaplan-Meier estimates of overall survival in the study cohort according to the integrated Molecular IPSS-R model. (**B**) Kaplan-Meier estimates of overall survival in the study cohort by IPSS-R scoring system.

Use of this scoring system stratified patients into 7 categories based on score (0, 0.5, 1, 1.5, 2, 2.5 and 3.5 points) with significantly distinct outcomes (*p* < 0.001) (Supplementary Figure 7, Supplementary Material). Patients could be grouped into three categories based on median OS (Table 3): lower risk (score 0–0.5, median OS: NR), intermediate risk (score 1–2, median OS: 29 months) and high risk (score 2.5–3.5, median OS: 12 months) with significantly different OS (Figure 4), and distinct LFS (Supplementary Figure 8, Supplementary Material). This MIPSSR model showed a trend to improved prognostic discrimination potential compared to IPSS-R as indicated by higher Dxy values (0.46 vs 0.43) and Cox model analysis (c-index 0.73 vs 0.71) (Supplementary Table 10, Supplementary Material). To further confirm its prognostic power, the new model was applied to each of the IPSS-R grouped categories. Only among patients with high or very-high risk IPSS-R did the new model predict for significant differences in OS (*p* = 0.033) (Supplementary Figure 9, Supplementary Material).

**Table 3:** New model incorporating IPSS-R and mutation variables

Score	N	Events	Category	Median OS (months)
0	26	5	Low	NR
0.5	22	7		
1	10	4	Int	29
1.5	33	16		
2	1	0		
2.5	20	17	High	12
3.5	2	2		

Low = Category Low of the new Molecular IPSS-R model based on OS of patients with scores 0–0.5. Int = Category Intermediate of the new Molecular IPSS-R model based on OS of patients with scores 1–2. High = Category High of the new Molecular IPSS-R model based on OS of patients with scores 2.5–3.5.

**Figure 5:**
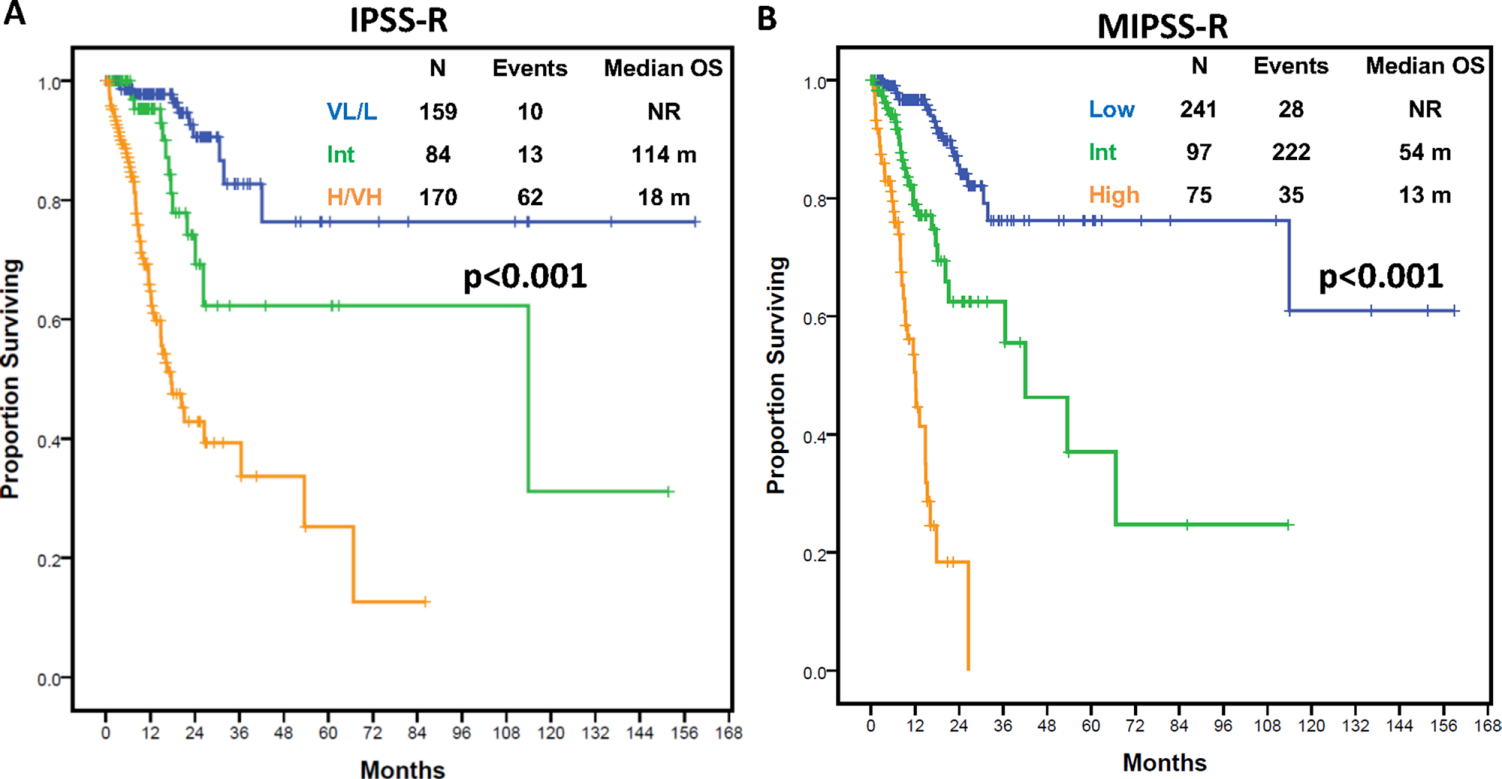
Overall survival outcomes by IPSS-R and molecular IPSS-R model in the additional cohort (**A**) Kaplan-Meier estimates of overall survival in the additional cohort according to the integrated Molecular IPSS-R model. (**B**) Kaplan-Meier estimates of overall survival in the additional cohort by IPSS-R scoring system.

To confirm the impact of the mutation number in survival of patients with MDS, we evaluated its impact within a separate cohort of 413 patients with MDS with genomic analysis at baseline. Patient characteristics can be found in Supplementary Material (Supplementary Table 11). A total of 272 patients (66%) had at least one detectable mutation with 30 patients (7%) having ≥ 3 mutations. Patients with ≥ 3 mutations had shorter OS (HR 2.84, 95% CI 1.53–5.26, *p* = 0.001, Supplementary Figure 10, Supplementary Data) and LFS (HR 2.86, 95% CI 1.62-5.07, *p* < 0.001). We applied the MIPSS-R to this validation cohort. Survival outcomes, in terms of OS and LFS, for each of the MIPSS-R and IPSS-R groups are shown in Figures 5 and Supplementary Figure 11 of Supplementary Material. As observed within the study cohort, MIPPS-R model was associated with higher discrimination potential for survival compared to IPSS-R (Dxy values 0.54 vs 0.51; c-index 0.77 vs 0.75) (Supplementary Table 12, Supplementary Material). Also, as in the discovery cohort, application of the new model to each IPSS-R grouped category rendered similar results, with the new model being able to show significant differences in survival among patients with high or very-high IPSS-R (3-year OS of 37% vs 0%, *p* < 0.001) (Supplementary Figure 12, Supplementary Material). To confirm survival differences were not due to IPSS-R categories, the new model was applied separately to patients with high or very-high IPSS-R. The new model identified patients with shorter than expected survival outcomes within both IPSS-R categories (median OS of 53.5 vs 12.3 months for intermediate and high MIPSS-R within high IPSS-R, *p* < 0.001; median OS of 16.5 and 8.8 months for intermediate vs high MIPSS-R within very-high IPSSR, *p* = 0.003) (Supplementary Figure 13).

## DISCUSSION

In the current study, we conducted WES of 114 patients with MDS or MDS/MPN to describe their mutation profile and clonal architecture at the time of diagnosis and correlate this with clinical outcomes. By doing so, we confirmed the findings of previous studies [4, 5] evaluating the genomic landscape of MDS. Most importantly, we observed that not only some individual mutations have prognostic impact, but the number of driver mutations also determine survival outcomes. In view of these results, we developed a new scoring system that incorporates mutation data into the current IPSS-R. This model was capable of stratifying higher-risk patients with a better predictive potential than IPSS-R. We were then able to apply this model into a separate cohort of patients with similar results.

Although a number of mutations have been identified by recent sequencing studies [3, 5, 7, 8, 24–26], few driver mutations have been shown to be consistently prognostic (e.g. *TP53* and *SF3B1*). The use of targeted amplicon-based next generation sequencing evaluating a set number of genes has allowed to perform mutational evaluation in a more systematic manner but limits our ability to identify novel or less common mutations with possible oncogenic potential. To evaluate the use of a more comprehensive sequencing approach which investigates all reported possible driver mutations we performed whole exome sequencing of baseline bone marrow samples. Although we could identify mutations involving less commonly mutated genes such as *DDX41* or *ETNK1*, the use of WES did not provide significantly more data than that obtained from previous studies evaluating a sufficiently large number of genes on a targeted NGS platform. This suggests use of WES may not be required to perform an adequate genomic evaluation of patients with MDS, and that targeted next-generation sequencing may be sufficient to perform a comprehensive mutation evaluation in the clinical setting.

In our study, we not only focused on individual mutations, but we also analyzed the prognostic impact of number of mutations and their clonal size. The possibility that the number of driver mutations is relevant in prognosis has been suggested by previous studies [5, 27]. Given the strong prognostic value of complex karyotype in MDS, it is conceivable that the number of driver mutations could also have impact on prognosis. In our data, having 3 or more driver mutations had prognostic impact both in the discovery cohort and validation cohort. However, due to the patient numbers in our study population, subset analysis among patients with MDS or MDS/MPN, although revealing worse outcomes among patients with CMML with 3 or more mutations, did not have sufficient power to consistently confirm these findings. In addition, although mutations in *STAG2* seemed to be associated with worse outcomes by univariate analysis, they did not retain this impact after multivariate analysis suggesting larger patient populations will need to be evaluated to confirm these findings.

Previous studies have reported the potential of mutation data as biomarkers of response to hypomethylating agents. Mutations in *TET2* [15] particularly at a VAF > 10% when present in the absence of co-occurring *ASXL1* mutations [12] and, more recently, mutations in *TP53* [13, 14] have been associated with response patterns to these agents. In our study, we were also able to identify specific mutational events which could predict for response outcomes and response duration in patients treated with hypomethylating agents. In our cohort, the number of detectable mutations predicted for lower likelihood of response and complete response as well as shorter response duration to hypomethylating agent therapy further suggesting the role of mutation burden on disease outcomes in MDS, and the potential use of sequencing data to predict patient outcomes.

Clonal heterogeneity defines diversification and evolution of founder clones by which daughter clones accumulate additional aberrations conferring a proliferative or survival advantage. Previous studies have confirmed the prognostic relevance of clonal heterogeneity determined by the presence of additional cytogenetic abnormalities in patients with acute myeloid leukemia [28]. Additionally, recent data suggests dynamic clonal changes during therapy and increase in mutational hierarchy complexity may drive progression and resistance in patients with MDS [29]. Although increased clonal complexity, as defined by the presence of multiple clones, was associated with a trend to shorter LFS and OS in our study, we were not able to confirm its independent adverse impact in prognosis. However, we acknowledge that the size of our patient population limits our power to confirm this finding.

Although the IPSS-R was developed evaluating a cohort of 7012 patients with MDS and did not include CMML or other MDS/MPN patients, a recent study has shown the validity of IPSS-R in patients with a diagnosis of CMML [11, 30]. Due to this previous data suggesting IPSS-R may be a valid prognostic score in CMML, and to increase our power to determine the prognostic impact of mutations we included both patients with MDS and CMML in our model comparison. By incorporating mutation data into the current IPSS-R we could improve our ability to predict the prognosis of patients with higher-risk MDS. Based on our results, patients with high or very high risk IPSS-R with *TP53* mutations and/or a high number of driver mutations, particularly if 3 or more, showed shorter than expected survival and could be up-scaled to a higher risk prognostic category within the new molecular model. This data suggests that a high number of mutations seems to have almost equivalent prognostic impact as *TP53*_mut_, and that the negative prognostic impact of both seems to be additive. This is especially relevant because patients with 3 or more mutations tend not to have complex karyotype, *TP53* or *SF3B1* mutations, suggesting that it identifies distinct subgroup of patients with significantly worse outcomes than would be otherwise expected by purely clinical or cytogenetic parameters.

We acknowledge our study has several limitations. First, our WES sample size is not sufficiently large to generate definite data and, like previous studies, included patients both with MDS and MDS/MPN. Therefore, further studies are required to reproduce our results. In addition, despite the restrictive mutation calling process followed in our study, the absence of matched normal control sample limits our ability to determine with certainty the somatic origin of some of the identified mutations. Also, our cohort included less patients with *SF3B1* and *DNMT3A* mutations compared to other genomic studies [4, 5]. The lower frequency of *SF3B1* mutations is likely due to the small numbers of patients with MDS-RS and a skew towards higher-risk patients in our discovery cohort. It is possible that the lower frequency of *DNMT3A* mutations in our study could be associated: 1) to the lower frequency of patients with MDS-RS in whom *DNMT3A* mutations frequently associate with *SF3B1* mutations(7) and 2) to the overall smaller patient cohort and the lower median age of included patients (66 years compared to 68 years in the study by Papaemmanuil et al and 72.5 years in the study by Haferlach et al) as, mutations in *DNMT3A*, are typical of age-related clonal hematopoiesis and could be more prevalent in older individuals. The presence of lower frequency of *SF3B1* mutations limits our ability to extrapolate our results to a lower-risk population of patients. Although we confirmed the prognostic relevance of mutation number on a separate cohort of patients, the sequencing technique used in this validation cohort only targeted a limited number of genes and did not include relevant genes such as splicing factors (*SF3B1, U2AF1, SRSF2, ZRSR2*), *BCOR* or cohesion genes, therefore significantly limiting our ability to identify patients truly exhibiting 3 or more mutations and validate our findings. Validation in an independent cohort of patients using a more expanded panel would therefore be required. Despite these limitations, we were still able to confirm the impact of mutation number in the validation set.

In conclusion, our study provides additional data determining the prognostic and predictive value of mutations in patients with MDS and shows that incorporation of mutation data into existing risk models can improve prognostication of patients with MDS. Furthermore, like cytogenetic abnormalities, the number of driver mutations have independent prognostic impact in MDS which could be equivalent to that of *TP53* mutations and which could allow identification of a subset of high-risk MDS patients with worse than expected survival outcomes. Future multicenter studies aimed at developing a new prognostic model incorporating molecular data generated by use of a uniform sequencing technology will be able to define the prognostic impact of mutations in a more definite manner and substitute our current clinical prognostic models.

## MATERIALS AND METHODS

### Patients and samples

We studied 114 consecutive patients with previously untreated MDS who were referred to The University of Texas MD Anderson Cancer Center (MDACC) between 2011 and 2014. Median duration from outside diagnosis to MDACC presentation was 2 months (range: 0–87 months). Patients had diagnostic bone marrow aspiration and biopsy at the time of presentation to MDACC. Informed consent was obtained according to protocols approved by the institutional review board in accordance with the Declaration of Helsinki. Diagnosis of MDS or CMML was confirmed in the hematopathology laboratory at MDACC. Cases were classified using the revised 2016 WHO classification [31]. Cytogenetic analysis was performed at the Clinical Cytogenetic Laboratory at MDACC and reported following ISCN 2013 Nomenclature [32]. Prognostic risk was calculated using both IPSS(1) and IPSS-R(2). As a validation cohort, we studied 413 patients with untreated MDS and CMML who were referred to MDACC.

### Whole exome sequencing and variant calling

Genomic DNA was extracted from whole bone marrow aspirate samples using Autopure Extractor (QIAGEN). Exome capture hybrid was performed with using Agilent SureSelect All Exon V4. Illumina HiSeq 2000 sequencer was used for sequencing with 75 base pair paired end read. Sequencing data was aligned to the hg19 human genome reference using Burrows-Wheeler Aligner (BWA) [33] followed by mark duplication, indel realignment, and base recalibration using Genome Analysis Toolkit (https://www.broadinstitute.org/gatk/guide/best-practices?bpm=DNAseq). The resulting BAM [34] files were preprocessed and base substitutions and small insertions/deletions were called using Mutect [35] and Pindel [36], respectively. To overcome the lack of matched normal sample, we generated virtual common normal sequence using in-house pooled normal sequence. This method has been shown to function as almost equivalent as matched normal to call somatic variants [37]. Since we intended to use mutation data for potential clinical application, we wanted to be conservative on variant annotation. Therefore, we modified approach used by Pappaemanuil et al. (5) to call high-confidence driver mutations (Supplementary Methods).

### Targeted gene sequencing analysis in validation cohort

Genomic DNA was extracted from whole bone marrow aspirate samples and was subject to 28-gene targeted PCR-based sequencing using a next generation sequencing (NGS) platform as previously described [38]. Evaluated genes included: *ABL1, ASXL1, BRAF, DNMT3A, EGFR, EZH2, FLT3, GATA2, HRAS, IDH1, IDH2, IKZF2, JAK2, KIT, KRAS, MDM2, MLL, MPL, MYD88, NOTCH1, NPM1, NRAS, PTPN11, RUNX1, TET2, TP53, WT1*. This analysis was performed within our CLIA-compliant molecular diagnostics laboratory after informed consent (for additional data see Supplementary Material). The limit of detection for SNVs was a tumor allelic frequency of at least 5%. Mutation calling was based on previously reported somatic mutations as registered in the Catalogue of Somatic Mutations in Cancer.

### Pathway definition

Pathways were defined as previously described (4, 5) and included: RNA splicing (*SF3B1, SRSF2, U2AF1,* and *ZRSR2*), DNA methylation (*DNMT3A, IDH1, IDH2* and *TET2*), chromatin regulation (*ASXL1, EZH2* and *MLL3*), transcription regulation (*BCOR, CEBPA , CUX1, ETV6, GATA2, PHF6, RUNX1* and *WT1*), kinase signaling (*CBL, FLT3, JAK2, KRAS, PTPN11, NF1, NRAS* and *SETBP1*), cohesin pathway (*STAG2*), DNA damage (*ATRX* and *TP53*) and other (*DDX41 and ETNK1*).

### Statistical analysis

Variant allele frequency (VAF) estimates were used to evaluate clonal and subclonal variant relationships within each individual sample (5). Clonal relationships were tested using Pearson goodness-of-fit tests with heterogeneity being defined in cases with goodness-of-fit *p* values < 0.05. Overall survival (OS) was calculated as the number of months from MDS diagnosis to death or last follow-up date. Leukemia-free survival (LFS) was calculated as the number of months from MDS diagnosis to transformation to leukemia or death. Patients who were alive at their last follow-up were censored on that date. Response was defined following 2006 IWG criteria [39]. Generalized linear models using logistic regression were used to study the association of overall response rates (ORR), complete response (CR) and risk factors. Competing risk analysis was performed to study association of risk factors with response duration. The Kaplan-Meier product limit method [40] was used to estimate the median OS and LFS for each clinical/demographic factor. Univariate Cox proportional hazards regression was used to identify any association with each of the variables and survival outcomes. Mutations present in less than 5 patients were not included in the analysis to prevent biased associations due to small numbers. For the OS multivariate analyses, we used imputation methods to replace missing values. The concordance index determined by Harrel’s C concordance and Somer’s D along with their 95% CI were used for model comparison [41]. Statistical analysis was performed using STATA/SE version 14.1 statistical software (Stata Corp. LP, College Station, TX) and R software version 2.15.0 (The R Foundation for Statistical Computing).

## SUPPLEMENTARY MATERIALS FIGURES AND TABLES




















