# Supplementary material for: Impact of the number of mutations in survival and response outcomes to hypomethylating agents in patients with myelodysplastic syndromes or myelodysplastic/myeloproliferative neoplasms
Source: Oncotarget. 2018 Jan 3;9(11):9714–27. doi: 10.18632/oncotarget.23882 (PMC5839396; doi:10.18632/oncotarget.23882)
Supplement: Supplementary file 1 [file oncotarget-09-9714-s001.pdf]

Impact of the number of mutations in survival and response outcomes to hypomethylating agents in patients with myelodysplastic syndromes or myelodysplastic/myeloproliferative neoplasms

SUPPLEMENTARY MATERIALS

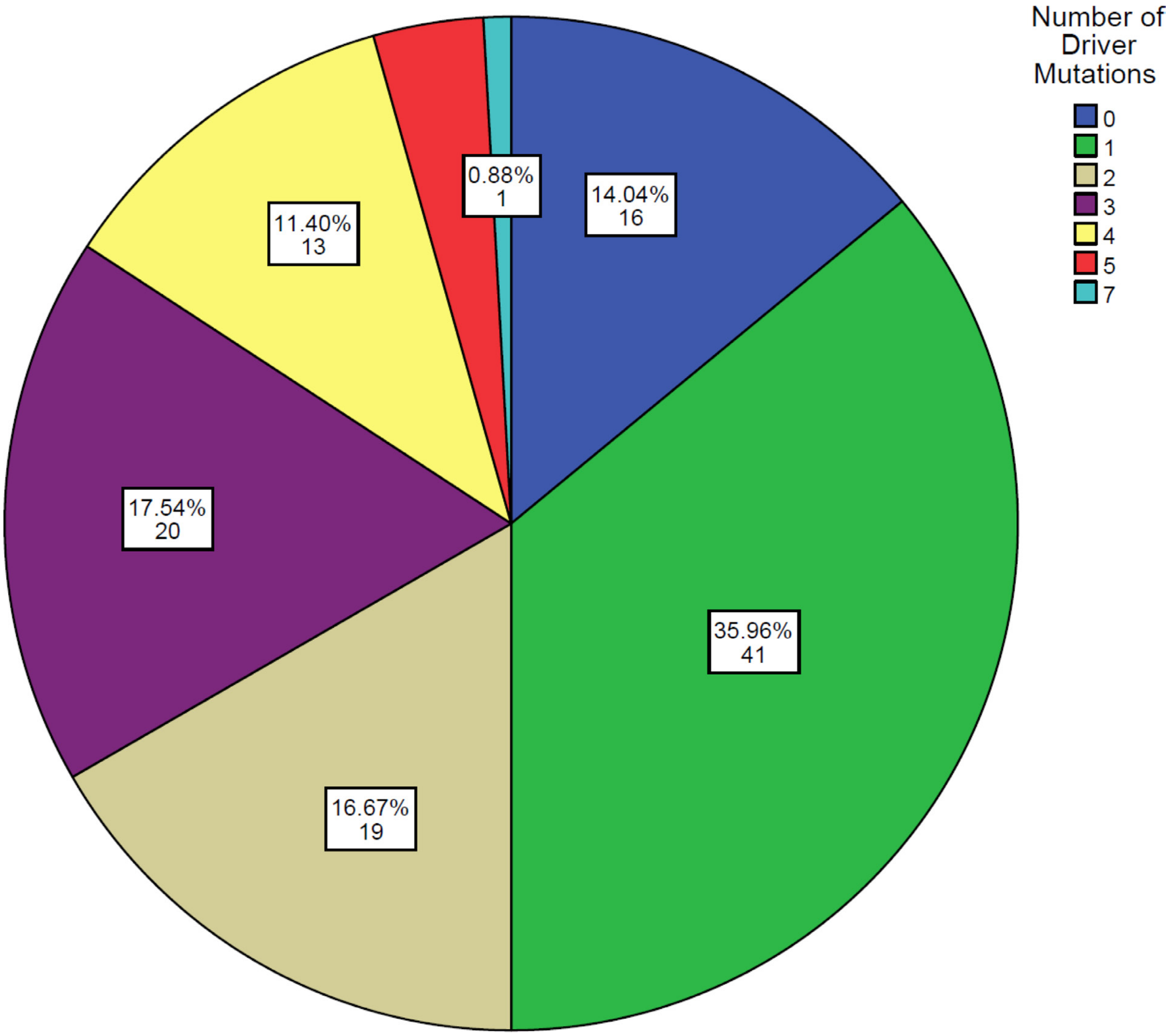

Supplementary Figure 1: Number of driver mutations per patient.

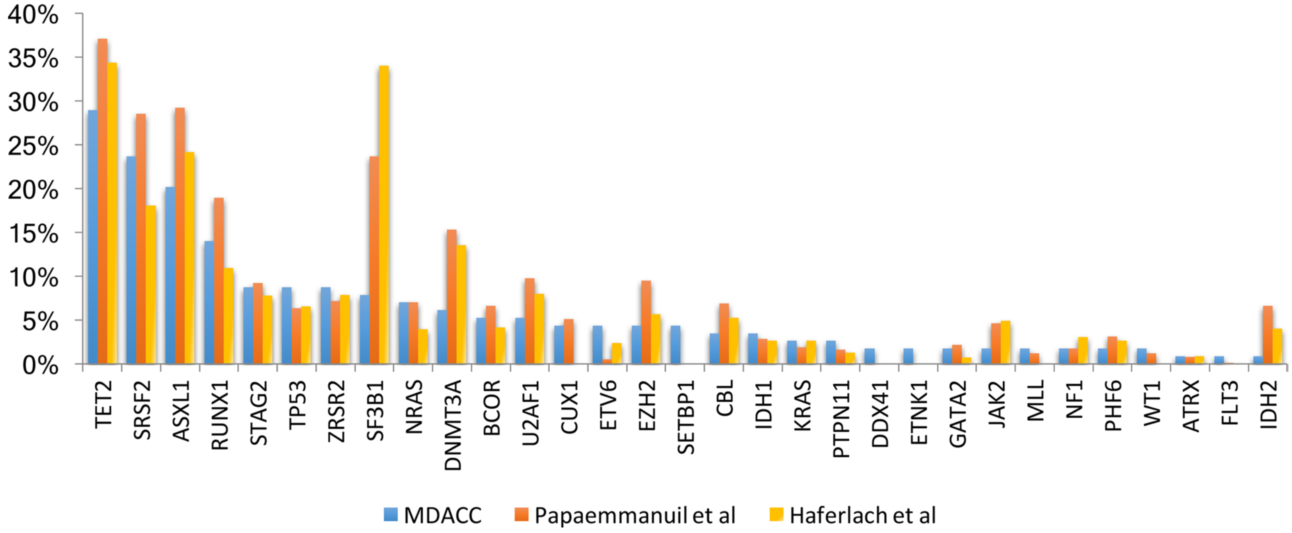

Supplementary Figure 2: Comparison of detected mutations with previous sequencing studies.

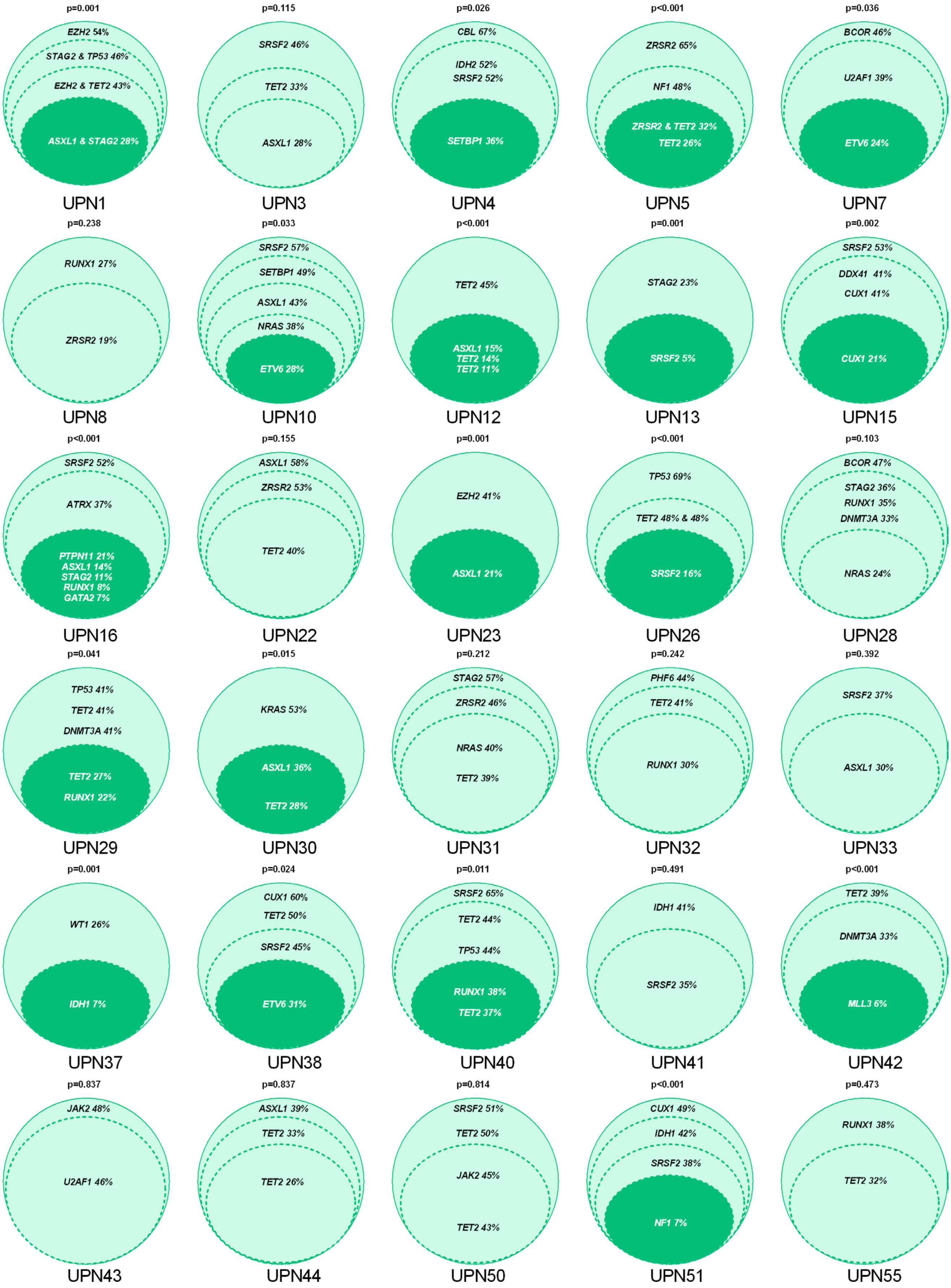

p=0.001
EZH2 54%
STAG2 & TP53 46%
EZH2 & TET2 43%
ASXL1 & STAG2 28%
UPN1
p=0.115
SRSF2 46%
TET2 33%
ASXL1 28%
UPN3
p=0.026
CBL 67%
IDH2 52%
SRSF2 52%
SETBP1 36%
UPN4
p<0.001
ZRSR2 65%
NF1 48%
ZRSR2 & TET2 32%
TET2 26%
UPN5
p=0.036
BCOR 46%
U2AF1 39%
ETV6 24%
UPN7
p=0.238
RUNX1 27%
ZRSR2 19%
UPN8
p=0.033
SRSF2 57%
SETBP1 49%
ASXL1 43%
NRAS 38%
ETV6 28%
UPN10
p<0.001
TET2 45%
ASXL1 15%
TET2 14%
TET2 11%
UPN12
p=0.001
STAG2 23%
SRSF2 5%
UPN13
p=0.002
SRSF2 53%
DDX41 41%
CUX1 41%
CUX1 21%
UPN15
p<0.001
SRSF2 52%
ATRX 37%
PTPN11 21%
ASXL1 14%
STAG2 11%
RUNX1 8%
GATA2 7%
UPN16
p=0.155
ASXL1 58%
ZRSR2 53%
TET2 40%
UPN22
p=0.001
EZH2 41%
ASXL1 21%
UPN23
p<0.001
TP53 69%
TET2 48% & 48%
SRSF2 16%
UPN26
p=0.103
BCOR 47%
STAG2 36%
RUNX1 35%
DNMT3A 33%
NRAS 24%
UPN28
p=0.041
TP53 41%
TET2 41%
DNMT3A 41%
TET2 27%
RUNX1 22%
UPN29
p=0.015
KRAS 53%
ASXL1 36%
TET2 28%
UPN30
p=0.212
STAG2 57%
ZRSR2 46%
NRAS 40%
TET2 39%
UPN31
p=0.242
PHF6 44%
TET2 41%
RUNX1 30%
UPN32
p=0.392
SRSF2 37%
ASXL1 30%
UPN33
p=0.001
WT1 26%
IDH1 7%
UPN37
p=0.024
CUX1 60%
TET2 50%
SRSF2 45%
ETV6 31%
UPN38
p=0.011
SRSF2 65%
TET2 44%
TP53 44%
RUNX1 38%
TET2 37%
UPN40
p=0.491
IDH1 41%
SRSF2 35%
UPN41
p<0.001
TET2 39%
DNMT3A 33%
MLL3 6%
UPN42
p=0.837
JAK2 48%
U2AF1 46%
UPN43
p=0.837
ASXL1 39%
TET2 33%
TET2 26%
UPN44
p=0.814
SRSF2 51%
TET2 50%
JAK2 45%
TET2 43%
UPN50
p<0.001
CUX1 49%
IDH1 42%
SRSF2 38%
NF1 7%
UPN51
p=0.473
RUNX1 38%
TET2 32%
UPN55

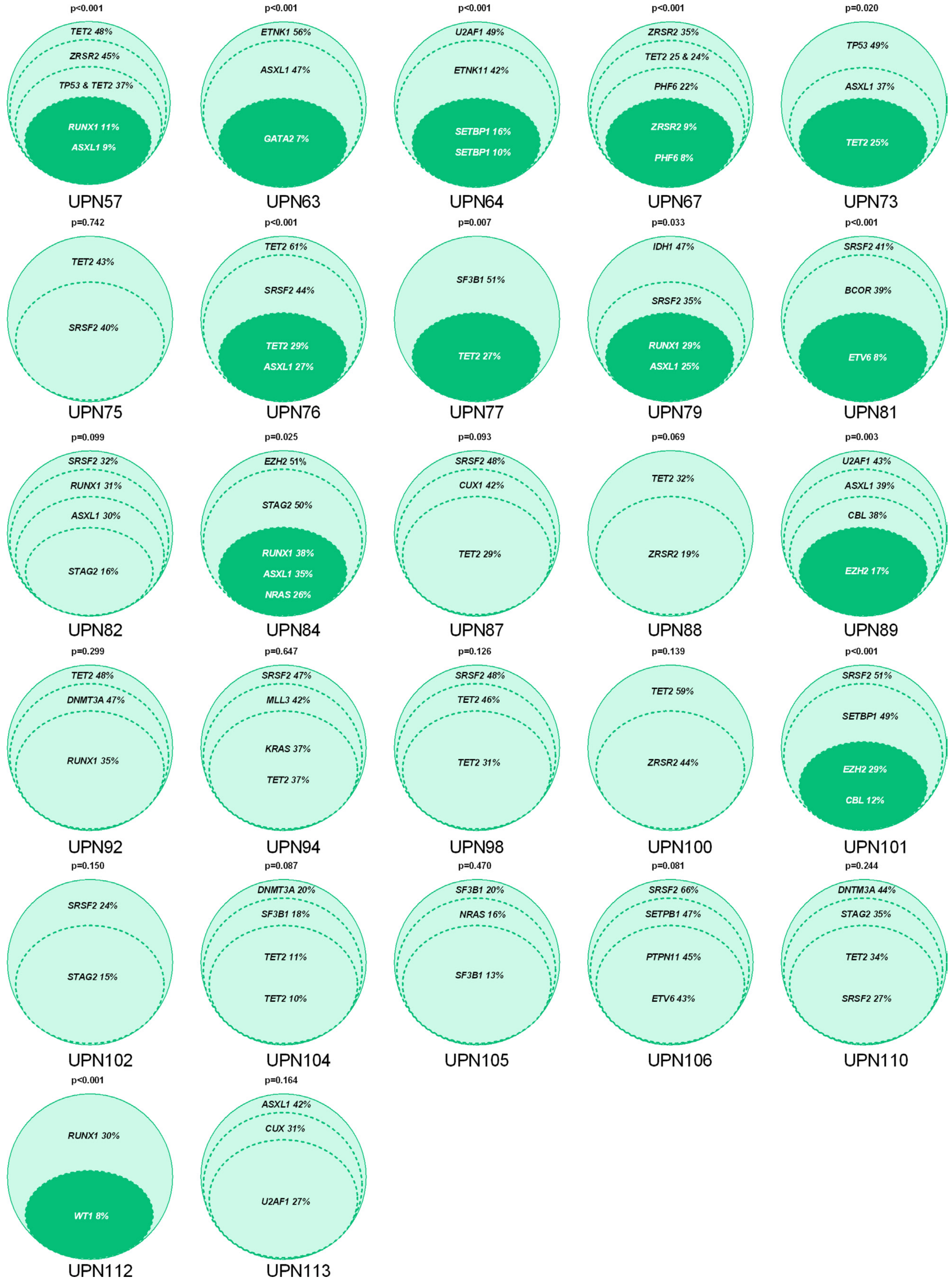

Supplementary Figure 3: Clonal composition of patients with 2 or more mutations. Percentage details the variant allele frequencies of detected mutations. *P* values correspond to those obtained by Pearson's goodness-of-fit Chi squared to determine clonal relationshipts.

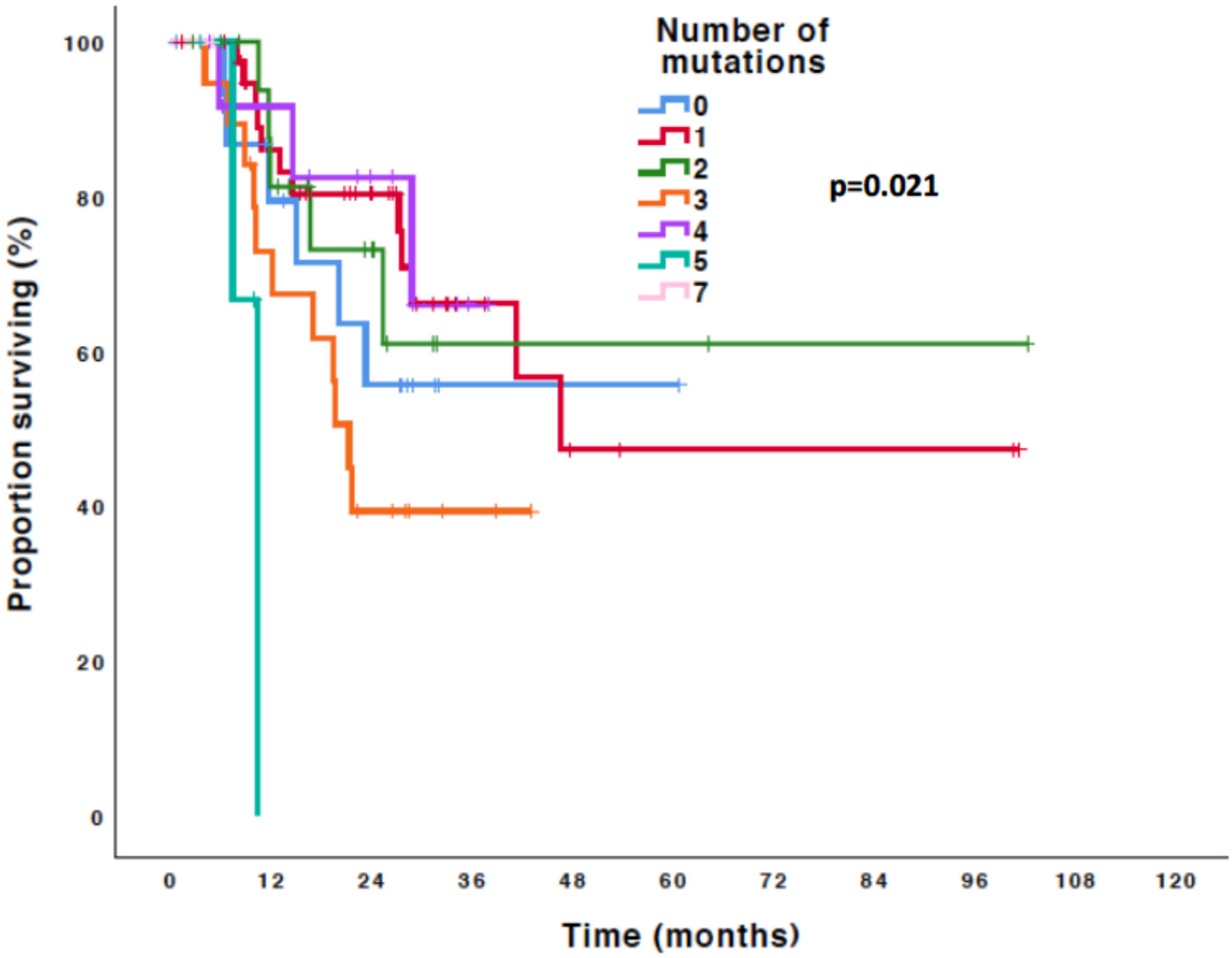

Supplementary Figure 4: Impact of number of driver mutations on leukemia-free survival.

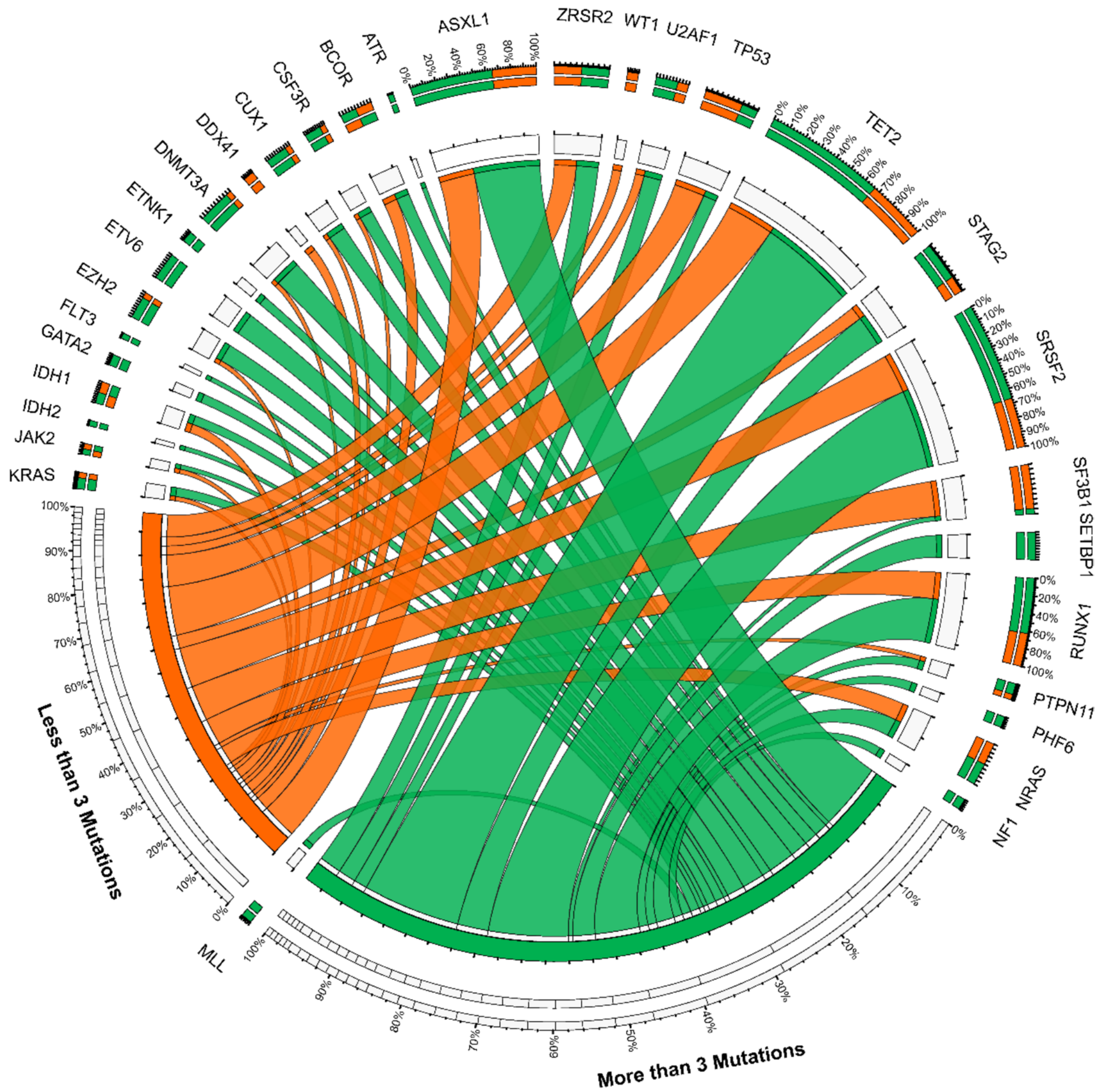

Supplementary Figure 5: Mutation distribution in patients with 3 or more mutations vs < 3 mutations.

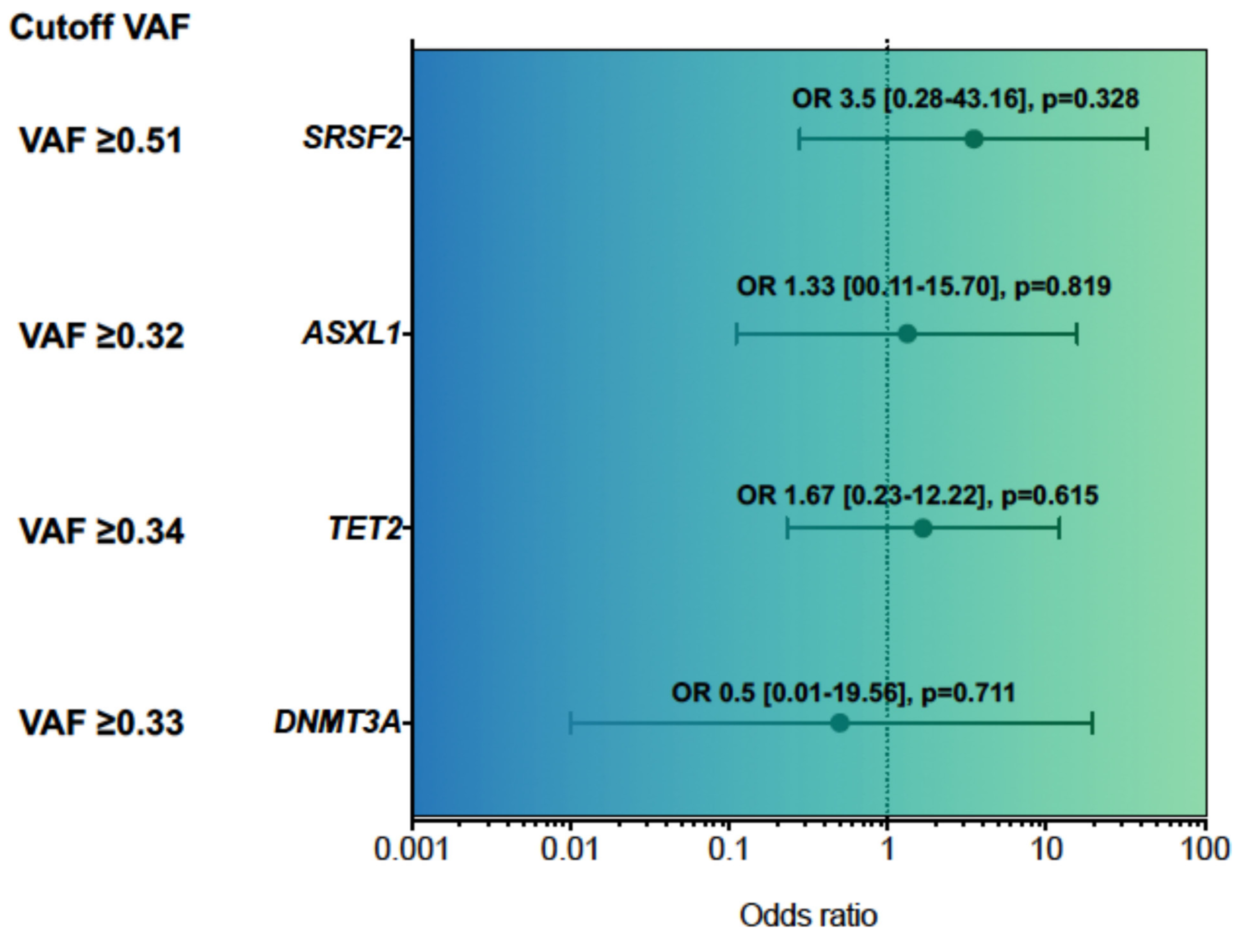

Supplementary Figure 6: Cut-off values of VAF for each given mutation and their impact in response to HMAs.

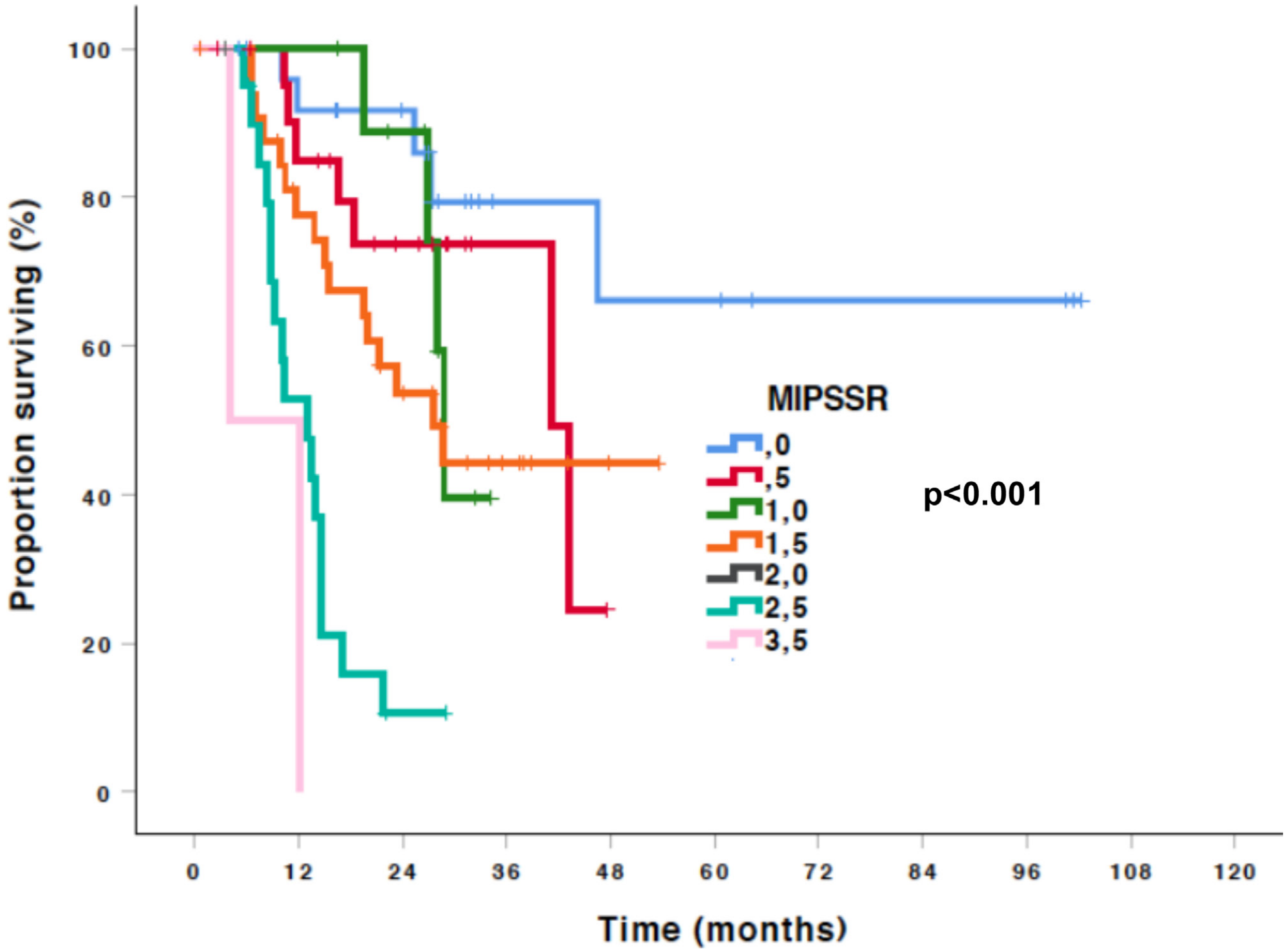

Supplementary Figure 7: Survival by MIPSS-R score: kaplan-meier curve for overall survival based on the attributed score following the generated molecular IPSS-R.

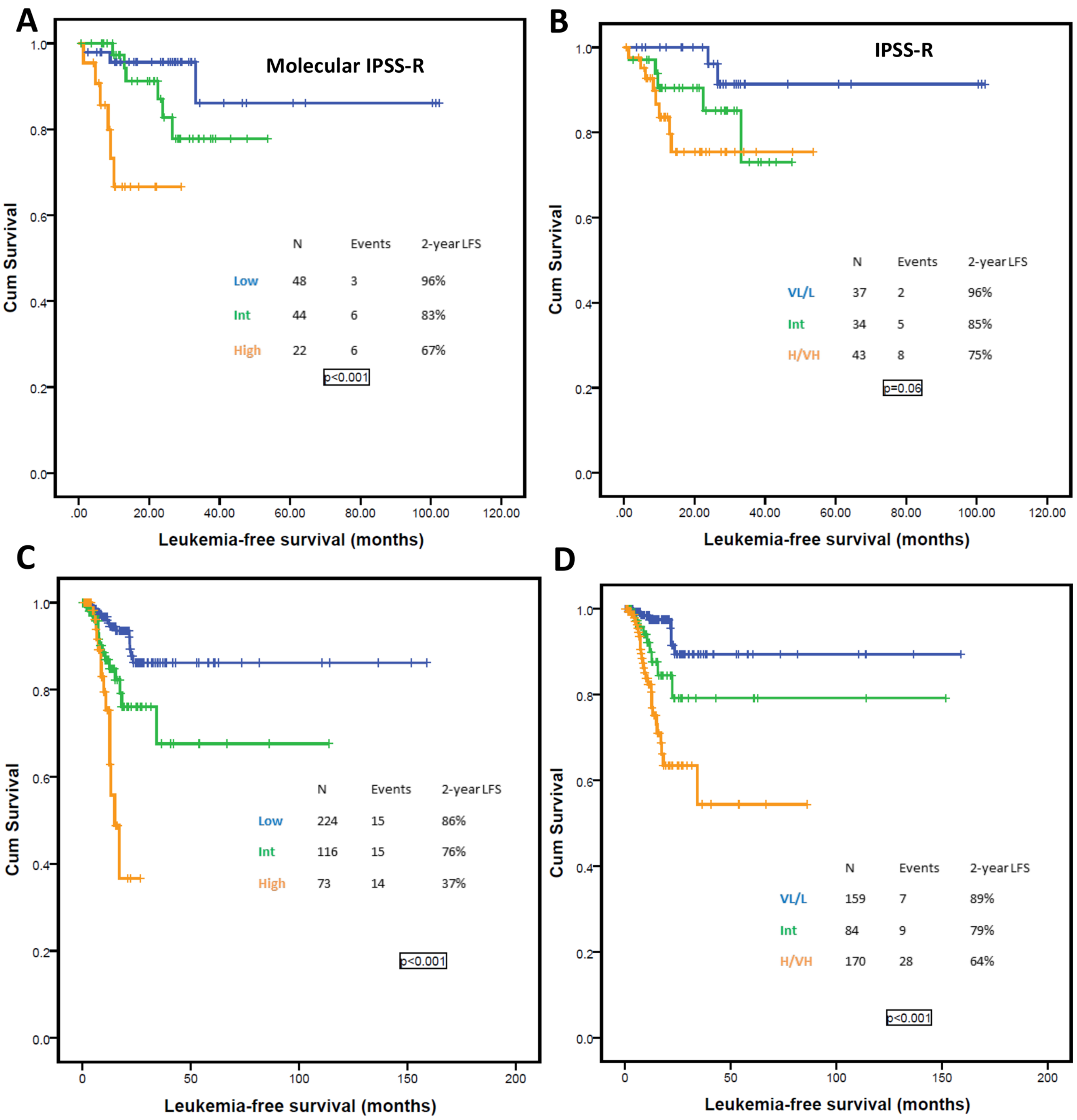

Supplementary Figure 8: Leukemia-free survival outcomes by integrated IPSS-R and molecular IPSS-R model. (**A**) Kaplan-Meier estimates of leukemia-free survival in the study cohort according to the integrated Molecular IPSS-R model. (**B**) Kaplan-Meier estimates of leukemia-free survival in the study cohort by IPSS-R scoring system. (**C**) Kaplan-Meier estimates of leukemia-free survival in the validation cohort according to the integrated Molecular IPSS-R Model. (**D**) Kaplan-Meier estimates of leukemia-free survival in the validation cohort by IPSS-R scoring system.

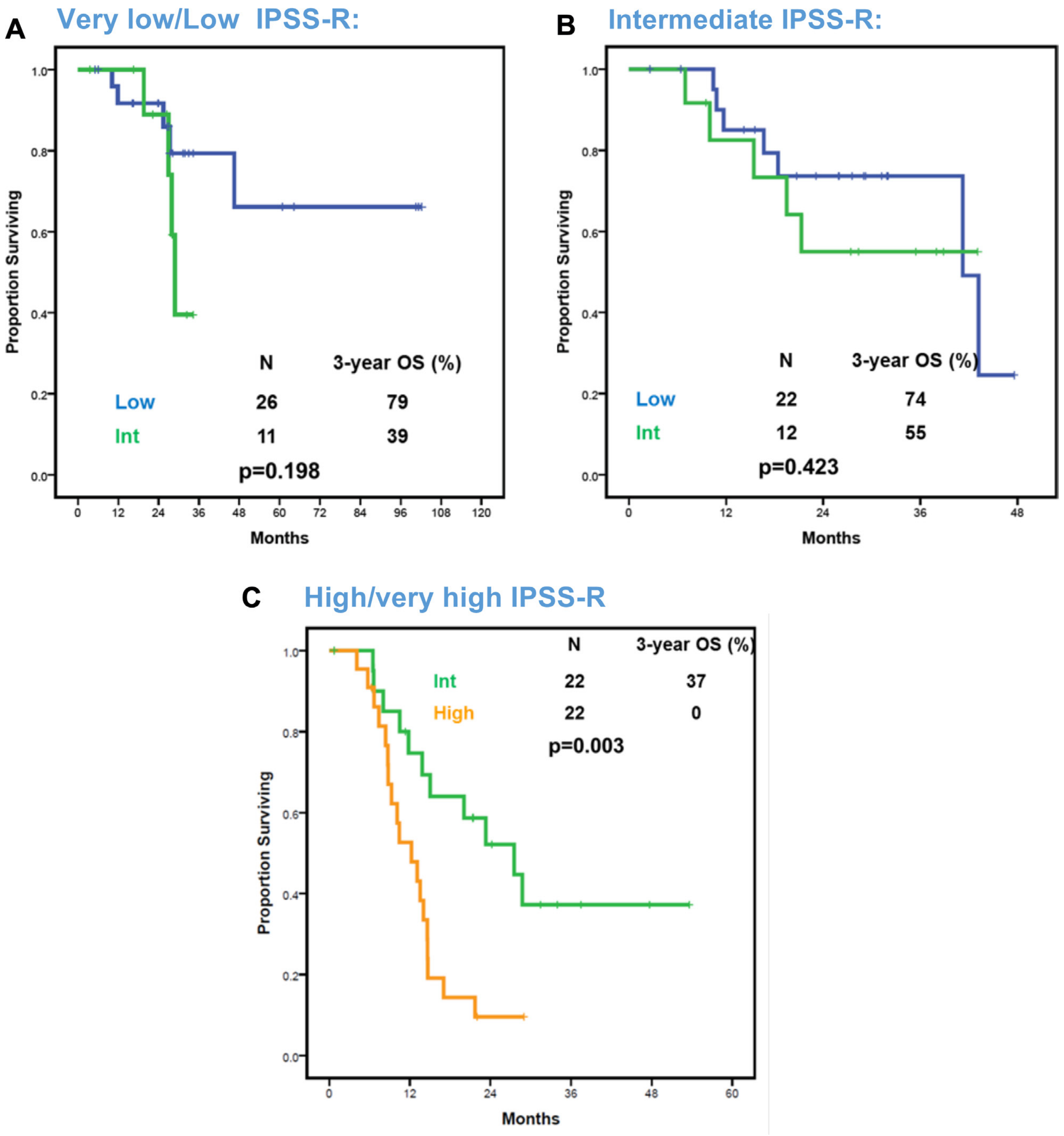

Supplementary Figure 9: Survival outcomes by MIPSS-R within each IPSS-R in the discovery cohort. (**A**) Very low/Low IPSS-R. (**B**) Intermediate IPSS-R. (**C**) High/very high IPSS-R.

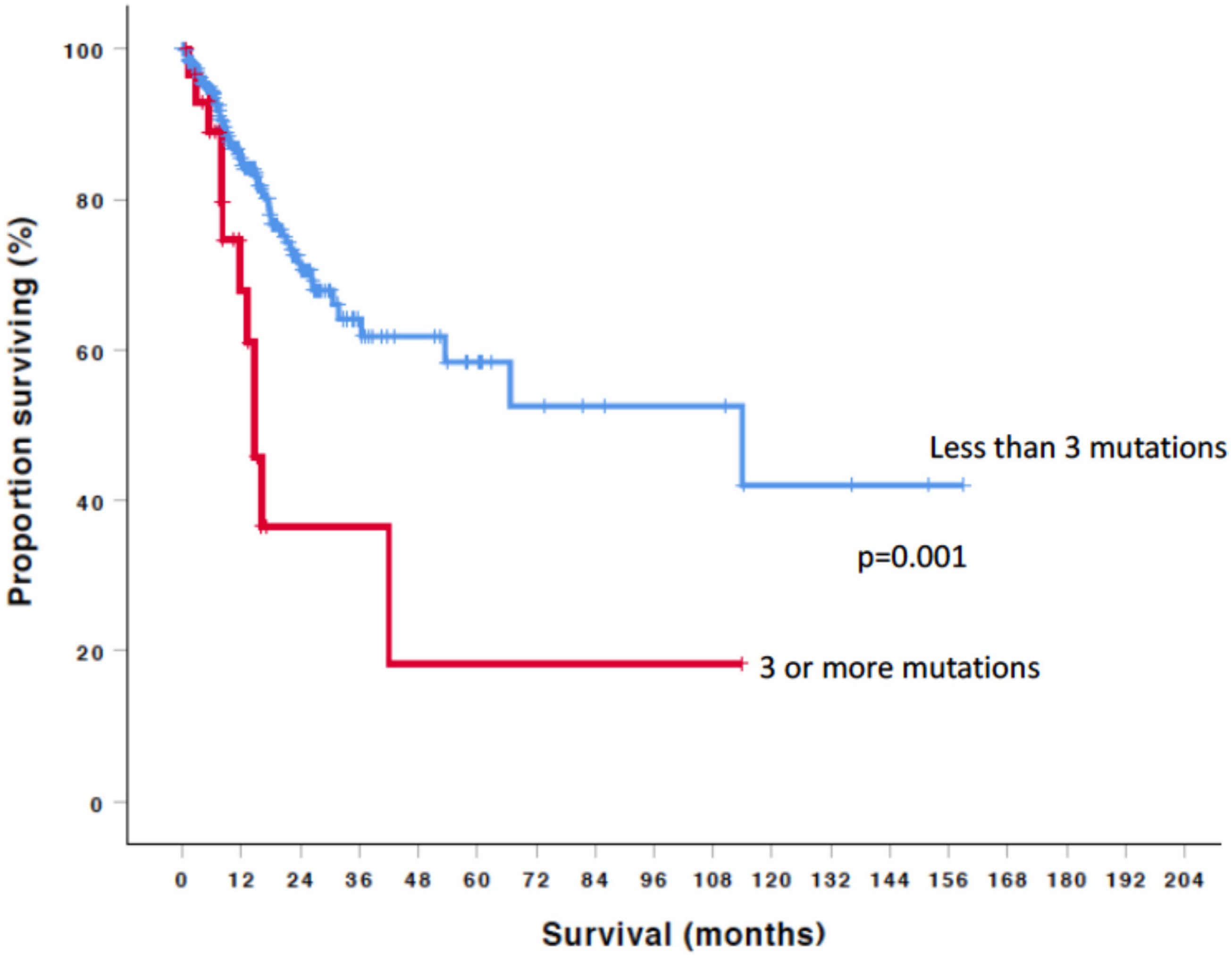

Supplementary Figure 10: Survival outcomes by presence of 3 or more mutations in the additional cohort.

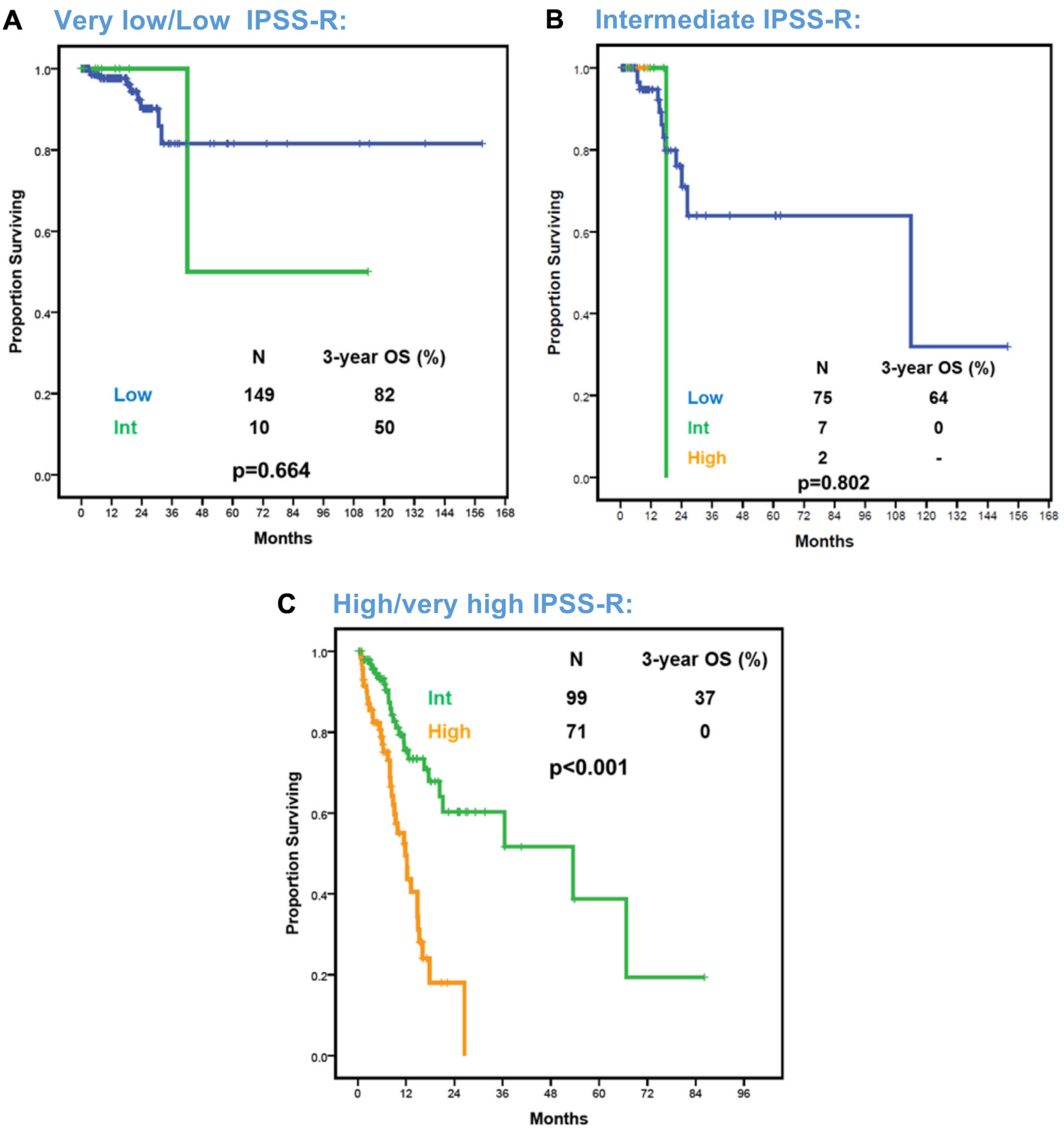

Supplementary Figure 11: Survival outcomes by MIPSS-R within each IPSS-R in the additional cohort. (**A**) Very low/Low IPSS-R: (**B**) Intermediate IPSS-R: (**C**) High/very high IPSS-R.

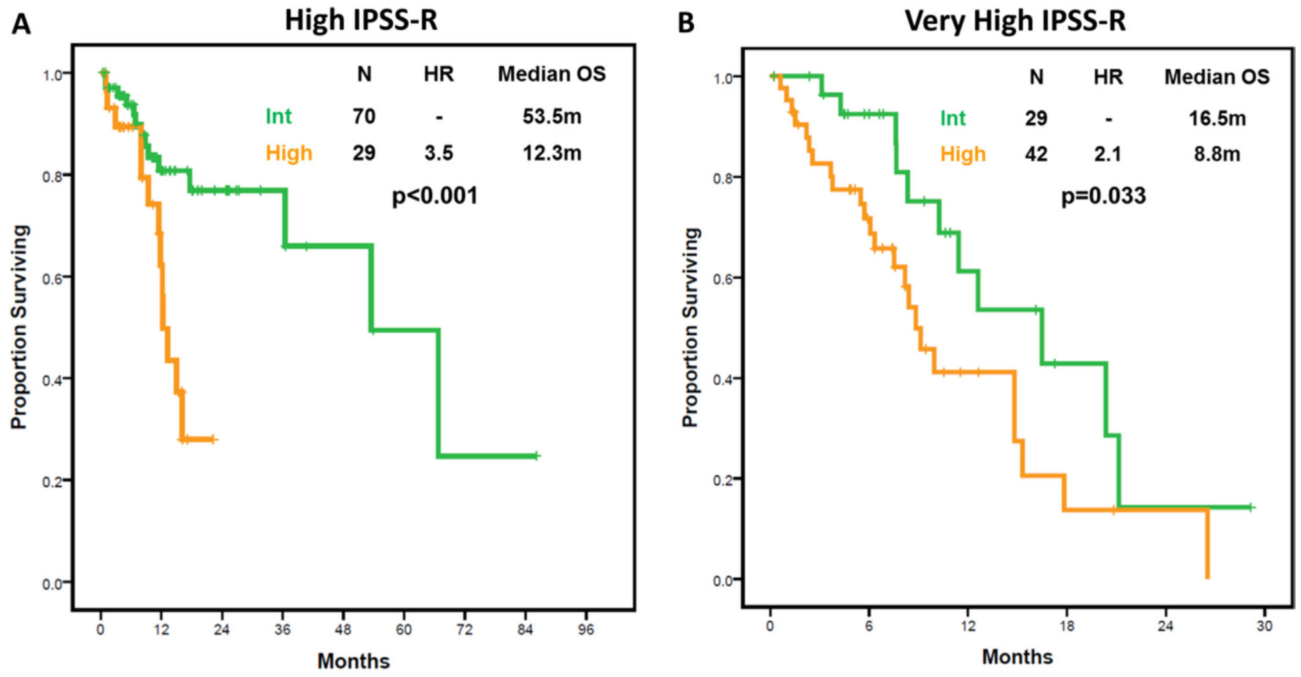

Supplementary Figure 12: Overall survival outcomes by the molecular IPSS-R model within high-risk MDS patients. (**A**) Kaplan-Meier estimates of overall survival in the validation cohort within patients with high risk IPSS-R according to the integrated Molecular IPSS-R model. (**B**) Kaplan-Meier estimates of overall survival in the additional cohort within patients with very high risk IPSS-R according to the integrated Molecular IPSS-R model.

Supplementary Table 1: Identified high-confidence mutations. See_Supplementary Table_1

Supplementary Table 2: Co-occurrence of mutation patterns and distribution of cytogenetic abnormalties. See_Supplementary Table_2

Supplementary Table 3: Univariate analysis for survival of identified mutations. See_Supplementary Table_3

Supplementary Table 4: Univariate analysis for survival of in patients with MDS. See_Supplementary Table_4

Supplementary Table 5: Univariate analysis for survival of in patients with MDS/MPN. See_ Supplementary Table_5

Supplementary Table 6: Univariate analysis for leukemia-free survival in patients with MDS. See_ Supplementary Table_6

Supplementary Table 7: Univariate analysis for leukemia-free survival in patients with MDS/ MPN. See_Supplementary Table_7

Supplementary Table 8: Characteristics of Patients with 3 or more mutations

Variable	<3 Mutations (*n* = 76) (%)	≥3 Mutations (*n* = 38) (%)	*p* value
WBC (x10^9/L)	3.6 (1–49.8)	2.9 (0.2–42.5)	0.031
MDS/MPN	16 (52)	15 (48)	0.037
Bone marrow blasts %	4 (0–20)	5 (2–18)	0.025
Normal karyotype	34 (47)	21 (57)	0.313
Complex karyotype	15 (21)	3 (8)	0.11
IPSS			
Low	14 (20)	8 (22)	
Int-1	28 (41)	17 (47)	0.840
Int-2	20 (29)	9 (25)	
High	7 (10)	2 (6)	
IPSS-R			
Very low	8 (11)	2 (8)	
Low	15 (21)	8 (22)	
Intermediate	21 (30)	12 (32)	0.527
High	16 (23)	12 (32)	
Very high	11 (16)	2 (5)	
HMA therapy	41 (77)	24 (86)	0.757
Response (ORR)	31 (63)	10 (37)	0.028
NR	19 (38)	17 (63)	
CR	25 (50)	5 (19)	0.014
CRp	4 (8)	1 (4)	
HI	2 (4)	4 (15)	
ASXL1	8 (11)	15 (40)	<0.001
CUX1	1 (1)	4 (11)	0.042
DNMT3A	1 (11)	6 (16)	0.005
ETV6	0 (0)	5 (23)	0.003
EZH2	1 (1)	4 (11)	0.042
RUNX1	6 (8)	10 (26)	0.008
SETBP1	0 (0)	5 (13)	0.03
SF3B1	8 (11)	1 (3)	0.133
SRSF2	9 (12)	18 (47)	<0.001
STAG2	3 (4)	7 (18)	0.015
TET2	11 (15)	22 (58)	<0.001
TP53	7 (9)	3 (8)	0.599

Supplementary Table 9: Univariate analysis for response outcomes after hypomethylating agent therapy. See_Supplementary Table_9

Supplementary Table 10: Model comparison study cohort

A: Univariate Analysis for Overall Survival using the current dataset

	N	Events	Median	log-rank	HR	95% CI for HR	*p*-value
Model 1: IPSSR							
VL	11	2	NR	<0.001			
L	23	6	NR		1.18	(0.24–5.85)	0.842
I	29	9	43.20		1.69	(0.36–7.84)	0.502
H	26	17	14.67		5.51	(1.27–23.92)	0.023
VH	13	9	13.83		6.10	(1.31–28.38)	0.021
Model 2: IPSS-R							
VL/L	34	8	NR	<0.001			
I	29	9	43.20		1.50	(0.58–3.90)	0.406
H/VH	39	26	14.60		5.06	(2.27–11.27)	<0.001
Model 3: IPSS-M							
Low	41	9	NR	<0.001			
Intermediate	42	18	28.80		2.59	(1.15–5.85)	0.022
High	19	16	13.10		11.43	(4.76–27.47)	<0.001

[*]If IPSS-R = I then Score = 0.5; If IPSS-R = H/VH then Score = 1.5; If TP53 = Positive then Score = 1; and If Mutations≥3 = Yes then Score = 1. If Score ≤ 0.5 then IPSS-M = Low; If Score ≥ 1 OR Score ≤ 2 then IPSS-M = Intermediate; and If Score ≥ 2.5 then IPSS-M = High.

B: Model Comparison using the current dataset

	Model 1	Model 2	Model 3	% change (Model 1 & Model 3)	% change (Model 2 & Model 3)
Harrell's C concordance statistic	0.7151	0.7133	0.7296	2.03	2.28
Somers' D	0.4301	0.4266	0.4591	6.74	7.62

Supplementary Table 11: Characteristics of patients in the additional cohort. See_Supplementary Table_11

Supplementary Table 12: Model comparison additional cohort

A: Univariate Analysis for Overall Survival using the new dataset

	N	Events	Median	log-rank	HR	95% CI for HR	*p*-value
Model 1: IPSS-R							
VL	42	3	NR	<0.001			
L	116	7	NR		0.74	(0.19– 2.87)	0.662
I	84	13	113.93		2.33	(0.66– 8.21)	0.190
H	99	27	53.50		5.35	(1.62–17.69)	0.006
VH	71	35	11.43		17.28	(5.25–56.87)	<0.001
Model 2: IPSS-R							
VL/L	158	10	NR	<0.001			
I	84	13	113.93		2.85	(1.25– 6.52)	0.013
H/VH	170	62	17.60		10.44	(5.31–20.55)	<0.001
Model 3: IPSS-M							
Low	224	21	NR	<0.001			
Intermediate	115	27	42.06		3.66	(2.05–6.53)	<0.001
High	73	37	12.18		15.34	(8.57–27.45)	<0.001

B: Model Comparison using the new dataset

	Model 1	Model 2	Model 3	% change (Model 1 & Model 3)	% change (Model 2 & Model 3)
Harrell's C concordance statistic	0.7910	0.7567	0.7727	2.31	2.11
Somers' D	0.5819	0.5134	0.5453	6.29	6.23
