# Supplementary material for: Impact of the number of mutations in survival and response outcomes to hypomethylating agents in patients with myelodysplastic syndromes or myelodysplastic/myeloproliferative neoplasms
Source: Oncotarget. 2018 Jan 3;9(11):9714–27. doi: 10.18632/oncotarget.23882 (PMC5839396; doi:10.18632/oncotarget.23882)
Supplement: Supplementary file 2 [file oncotarget-09-9714-s002.docx]

**Supplementary Table 1: Identified high-confidence mutations**

| **Patient** | **Diagnosis** | **Gene** | **Mutation location** | **Type of Mutation** | **cDNA Change** | **Amino acid change** |
| --- | --- | --- | --- | --- | --- | --- |
| 1 | MDS-RS | *SF3B1* | exonic | nonsynonymous SNV | c.A2098G | p.K700E |
| 2 | CMML | *ASXL1* | exonic | stopgain SNV | c.C2407T | p.Q803X |
|  |  | *TP53* | exonic | nonsynonymous SNV | c.A536G | p.H179R |
|  |  | *TET2* | exonic | frameshift deletion | c.1206delC |  |
| 3 | MDS-MLD | *EZH2* | exonic | stopgain SNV | c.C619T | p.R207X |
|  |  | *EZH2* | exonic | nonsynonymous SNV | c.G2069A | p.R690H |
|  |  | *STAG2* | exonic | stopgain SNV | c.C3133T | p.R1045X |
|  |  | *STAG2* | exonic | stopgain SNV | c.C2857T | p.R953X |
|  |  | *TET2* | exonic | nonsynonymous SNV | c.G3845A | p.R1282H |
|  |  | *TP53* | exonic | nonsynonymous SNV | c.C817T | p.R273C |
|  |  | *ASXL1* | exonic | frameshift deletion | c.1157_1158del |  |
| 4 | CMML | *SRSF2* | exonic | nonsynonymous SNV | c.C284T | p.P95L |
|  |  | *TET2* | splicing |  |  |  |
|  |  | *ASXL1* | exonic | frameshift deletion | c.3037delA |  |
| 5 | CMML | *CBL* | exonic | nonsynonymous SNV | c.T1210G | p.C404G |
|  |  | *IDH2* | exonic | nonsynonymous SNV | c.C418T | p.R140W |
|  |  | *SETBP1* | exonic | nonsynonymous SNV | c.G2608A | p.G870S |
|  |  | *SRSF2* | exonic | nonframeshift deletion | c.284_307del |  |
| 6 | MDS-EB | *NF1* | exonic | stopgain SNV | c.C2977T | p.Q993X |
|  |  | *ZRSR2* | splicing |  |  |  |
|  |  | *TET2* | exonic | stopgain SNV | c.1684delC |  |
|  |  | *TET2* | exonic | frameshift deletion | c.5187delT |  |
| 7 | MDS-EB | *ASXL1* | exonic | frameshift insertion | c.1925_1926insA |  |
| 8 | CMML | *U2AF1* | exonic | nonsynonymous SNV | c.C101A | p.S34Y |
|  |  | *BCOR* | exonic | frameshift insertion | c.4863_4864insG |  |
|  |  | *ETV6* | exonic | frameshift insertion | c.904_905insAGCC |  |
| 9 | MDS-SLD | *RUNX1* | exonic | frameshift insertion | c.525_526insT |  |
|  |  | *ZRSR2* | exonic | frameshift deletion | c.733delC |  |
| 10 | CMML | *ASXL1* | exonic | stopgain SNV | c.C2122T | p.Q708X |
|  |  | *NRAS* | exonic | nonsynonymous SNV | c.G34C | p.G12R |
|  |  | *SETBP1* | exonic | nonsynonymous SNV | c.G2608A | p.G870S |
|  |  | *SRSF2* | exonic | nonsynonymous SNV | c.C284A | p.P95H |
|  |  | *ETV6* | exonic | frameshift deletion | c.225delT |  |
| 11 | MDS-SLD | *NRAS* | exonic | nonsynonymous SNV | c.G34C | p.G12R |
| 12 | MDS-SLD | *ASXL1* | exonic | stopgain SNV | c.C2893T | p.R965X |
|  |  | *TET2* | exonic | frameshift deletion | c.4323delA |  |
|  |  | *TET2* | exonic | frameshift deletion | c.4778delG |  |
|  |  | *TET2* | exonic | frameshift insertion | c.4057_4058insTTAT |  |
| 13 | MDS-EB | *SRSF2* | exonic | nonsynonymous SNV | c.C284G | p.P95R |
|  |  | *STAG2* | exonic | frameshift insertion | c.3411_3412insT |  |
| 14 | MDS-RS | *SF3B1* | exonic | nonsynonymous SNV | c.A2098G | p.K700E |
| 15 | CMML | *CUX1* | exonic | stopgain SNV | c.C3019T | p.R1007X |
|  |  | *SRSF2* | exonic | nonsynonymous SNV | c.C284A | p.P95H |
|  |  | *CUX1* | exonic | frameshift deletion | c.2373delC |  |
| 16 | MDS | *PTPN11* | exonic | nonsynonymous SNV | c.A227T | p.E76V |
|  |  | *RUNX1* | exonic | stopgain SNV | c.C1106A | p.S369X |
|  |  | *SRSF2* | exonic | nonsynonymous SNV | c.C284A | p.P95H |
|  |  | *STAG2* | exonic | stopgain SNV | c.C3133T | p.R1045X |
|  |  | *ASXL1* | exonic | frameshift deletion | c.1888_1910del |  |
|  |  | *ATRX* | exonic | frameshift insertion | c.6020_6021insA |  |
|  |  | *GATA2* | exonic | nonframeshift insertion | c.1012_1013insCCACTCATCAAGCCCAAGCGA |  |
| 17 | MDS-RS | *SF3B1* | exonic | nonsynonymous SNV | c.A2098G | p.K700E |
| 18 | CMML | *ASXL1* | exonic | stopgain SNV | c.C1773G | p.Y591X |
|  |  | *TET2* | exonic | stopgain SNV | c.C1504T | p.Q502X |
|  |  | *ZRSR2* | exonic | frameshift insertion | c.288_289insA |  |
| 19 | MDS-EB | *ASXL1* | exonic | frameshift deletion | c.1888_1910del |  |
|  |  | *EZH2* | exonic | frameshift insertion | c.1897_1898insT |  |
| 20 | CMML | *SRSF2* | exonic | nonsynonymous SNV | c.C284A | p.P95H |
| 21 | MDS-MLD | *BCOR* | exonic | frameshift insertion | c.2211_2212insAA |  |
| 22 | MDS-EB | *SRSF2* | exonic | nonsynonymous SNV | c.C284A | p.P95H |
|  |  | *TET2* | exonic | stopgain SNV | c.G1755A | p.W585X |
|  |  | *TET2* | exonic | stopgain SNV | c.G3071A | p.W1024X |
|  |  | *TP53* | exonic | nonsynonymous SNV | c.G814A | p.V272M |
| 23 | MDS-EB | *KRAS* | exonic | nonsynonymous SNV | c.G35C | p.G12A |
| 24 | MDS-EB | *DNMT3A* | exonic | nonsynonymous SNV | c.G2645A | p.R882H |
|  |  | *NRAS* | exonic | nonsynonymous SNV | c.G37C | p.G13R |
|  |  | *STAG2* | splicing | splicing | c.2533+1G>A |  |
|  |  | *BCOR* | exonic | frameshift deletion | c.1384delG |  |
|  |  | *RUNX1* | exonic | frameshift deletion | c.809delC |  |
| 25 | MDS-EB | *DNMT3A* | exonic | nonsynonymous SNV | c.G2645A | p.R882H |
|  |  | *TET2* | exonic | stopgain SNV | c.G5854T | p.E1952X |
|  |  | *RUNX1* | exonic | frameshift insertion | c.1190_1191insC |  |
|  |  | *TET2* | exonic | frameshift deletion | c.3366delC |  |
| 26 | CMML | *KRAS* | exonic | nonsynonymous SNV | c.G37T | p.G13C |
|  |  | *ASXL1* | exonic | frameshift deletion | c.1888_1910del |  |
|  |  | *TET2* | exonic | frameshift deletion | c.1488delG |  |
| 27 | MDS-EB | *NRAS* | exonic | nonsynonymous SNV | c.G35A | p.G12D |
|  |  | *TET2* | exonic | nonsynonymous SNV | c.T3806C | p.L1269P |
|  |  | *ZRSR2* | exonic | stopgain SNV | c.C298T | p.Q100X |
|  |  | *STAG2* | exonic | frameshift deletion | c.3681delT |  |
| 28 | MDS-MLD | *PHF6* | exonic | stopgain SNV | c.C385T | p.R129X |
|  |  | *TET2* | exonic | stopgain SNV | c.C2689T | p.Q897X |
|  |  | *RUNX1* | exonic | frameshift deletion | c.292delC |  |
| 29 | CMML | *SRSF2* | exonic | nonsynonymous SNV | c.C283G | p.P95A |
|  |  | *ASXL1* | exonic | frameshift deletion | c.2354delT |  |
| 30 | MDS-EB | *TP53* | exonic | nonsynonymous SNV | c.G711A | p.M237I |
| 31 | MDS-EB | *ASXL1* | exonic | frameshift deletion | c.2083_2095del |  |
| 32 | MDS-EB | *IDH1* | exonic | nonsynonymous SNV | c.G395A | p.R132H |
|  |  | *WT1* | exonic | frameshift insertion | c.1143_1144insCGGTC |  |
| 33 | MDS-MLD | *CUX1* | exonic | stopgain SNV | c.C3733T | p.Q1245X |
|  |  | *SRSF2* | exonic | nonsynonymous SNV | c.C284A | p.P95H |
|  |  | *TET2* | exonic | nonsynonymous SNV | c.A5774G | p.H1925R |
|  |  | *ETV6* | exonic | frameshift insertion | c.770_771insCC |  |
| 34 | MDS-MLD | *PTPN11* | exonic | stopgain SNV | c.T591G | p.Y197X |
| 35 | MDS-SLD | *RUNX1* | exonic | nonsynonymous SNV | c.C422T | p.S141L |
|  |  | *SRSF2* | exonic | nonsynonymous SNV | c.C284T | p.P95L |
|  |  | *TET2* | exonic | nonsynonymous SNV | c.T3806C | p.L1269P |
|  |  | *TET2* | splicing |  |  |  |
| 36 | MDS-MLD | *IDH1* | exonic | nonsynonymous SNV | c.C394T | p.R132C |
|  |  | *SRSF2* | exonic | nonsynonymous SNV | c.C284A | p.P95H |
| 37 | MDS-EB | *DNMT3A* | exonic | nonsynonymous SNV | c.G2645A | p.R882H |
|  |  | *MLL3* | exonic | stopgain SNV | c.T930A | p.C310X |
|  |  | *TET2* | exonic | frameshift deletion |  | c.3792_3796del |
| 38 | MDS/MPN | *JAK2* | exonic | nonsynonymous SNV | c.G1849T | p.V617F |
|  |  | *U2AF1* | exonic | nonsynonymous SNV | c.A470G | p.Q157R |
| 39 | MDS-SLD | *ASXL1* | exonic | stopgain SNV | c.C1331G | p.S444X |
|  |  | *TET2* | exonic | stopgain SNV | c.T4074A | p.Y1358X |
|  |  | *TET2* | exonic | frameshift insertion | c.2388_2389insT |  |
| 40 | MDS-MLD | *NRAS* | exonic | nonsynonymous SNV | c.G37C | p.G13R |
| 41 | MDS-RS | *MLLT6* | exonic | frameshift deletion | c.901delA |  |
|  |  | *NFE2* | exonic | stopgain SNV | c.252_253insATAA |  |
| 42 | MDS-MLD | *TET2* | exonic | frameshift deletion | c.4725_4726del |  |
| 43 | MDS-SLD | *TP53* | exonic | nonsynonymous SNV | c.T526A | p.C176S |
| 44 | MDS-MLD | *RUNX1* | exonic | frameshift insertion | c.419_420insT |  |
| 45 | CMML | *JAK2* | exonic | nonsynonymous SNV | c.G1849T | p.V617F |
|  |  | *SRSF2* | exonic | nonsynonymous SNV | c.C284A | p.P95H |
|  |  | *TET2* | exonic | stopgain SNV | c.C1651T | p.Q551X |
|  |  | *TET2* | exonic | stopgain SNV | c.C2033A | p.S678X |
| 46 | MDS-RS | *IDH1* | exonic | nonsynonymous SNV | c.G395A | p.R132H |
|  |  | *CUX1* | exonic | frameshift insertion | c.1200_1201insCGCT |  |
|  |  | *NF1* | exonic | frameshift insertion | c.4791_4792insA |  |
|  |  | *SRSF2* | exonic | nonframeshift deletion | c.284_307del |  |
| 47 | MDS-MLD | *ASXL1* | exonic | frameshift deletion | c.1888_1910del |  |
| 48 | MDS/MPN-U | *RUNX1* | exonic | frameshift insertion | c.1245_1246insA |  |
|  |  | *TET2* | exonic | frameshift deletion | c.755delC |  |
| 49 | MDS-EB | *TET2* | exonic | stopgain SNV | c.G3484T | p.E1162X |
|  |  | *ZRSR2* | splicing |  |  |  |
|  |  | *ASXL1* | exonic | frameshift insertion | c.1872_1873insCGAGGGGCGAGAGGTCACCACTGCCATAGAGAGGCGG |  |
|  |  | *RUNX1* | exonic | frameshift insertion | c.464_465insG |  |
|  |  | *TET2* | exonic | frameshift insertion | c.5638_5639insT |  |
| 50 | CMML | *CBL* | exonic | nonsynonymous SNV | c.G1211A | p.C404Y |
| 51 | MDS/MPN-U | *ASXL1* | exonic | stopgain SNV | c.C1249T | p.R417X |
|  |  | *ETNK1* | exonic | nonsynonymous SNV | c.A731G | p.N244S |
|  |  | *GATA2* | exonic | frameshift deletion | c.194_209del |  |
| 52 | MDS-MLD | *ETNK1* | exonic | nonsynonymous SNV | c.A731G | p.N244S |
|  |  | *SETBP1* | exonic | nonsynonymous SNV | c.G2602A | p.D868N |
|  |  | *SETBP1* | exonic | nonsynonymous SNV | c.G2608A | p.G870S |
|  |  | *U2AF1* | exonic | nonsynonymous SNV | c.A470C | p.Q157P |
| 53 | MDS-EB | *TP53* | exonic | stopgain SNV | c.C528A | p.C176X |
| 54 | CMML | *TET2* | exonic | frameshift insertion | c.5727_5728insCCCA |  |
| 55 | MDS-EB | *PHF6* | exonic | nonsynonymous SNV | c.G824A | p.R275Q |
|  |  | *PHF6* | exonic | stopgain SNV | c.C925T | p.Q309X |
|  |  | *TET2* | exonic | stopgain SNV | c.C4663T | p.Q1555X |
|  |  | *TET2* | exonic | stopgain SNV | c.C5100G | p.Y1700X |
|  |  | *ZRSR2* | splicing |  |  |  |
|  |  | *ZRSR2* | splicing |  |  |  |
| 56 | CMML | *BCOR* | exonic | stopgain SNV | c.C1024T | p.R342X |
|  |  | *BCOR* | exonic | frameshift insertion | c.3664_3665insATGGGAGCAGCAG |  |
| 57 | CMML | *TET2* | exonic | stopgain SNV | c.C3709T | p.R1237X |
| 58 | MDS-MLD | *STAG2* | exonic | stopgain SNV | c.C1810T | p.R604X |
| 59 | CMML | *NRAS* | exonic | nonsynonymous SNV | c.G35A | p.G12D |
| 60 | CMML | *TP53* | exonic | nonsynonymous SNV | c.T487G | p.Y163D |
| 61 | MDS-MLD | *SF3B1* | exonic | nonsynonymous SNV | c.C1873T | p.R625C |
| 62 | CMML | *SRSF2* | exonic | nonsynonymous SNV | c.C284G | p.P95R |
|  |  | *TET2* | exonic | stopgain SNV | c.C2809T | p.Q937X |
| 63 | CMML | *SRSF2* | exonic | nonsynonymous SNV | c.C284A | p.P95H |
|  |  | *ASXL1* | exonic | frameshift deletion | c.2481delT |  |
|  |  | *TET2* | exonic | frameshift insertion | c.2008_2009insATGT |  |
|  |  | *TET2* | exonic | stopgain SNV | c.4741_4742insA |  |
| 64 | MDS-RS | *SF3B1* | exonic | nonsynonymous SNV | c.A1997G | p.K666R |
|  |  | *TET2* | exonic | frameshift deletion | c.2277delC |  |
| 65 | MDS-SLD | *RUNX1* | exonic | frameshift deletion | c.1189delC |  |
| 66 | MDS-MLD | *IDH1* | exonic | nonsynonymous SNV | c.G395A | p.R132H |
|  |  | *ASXL1* | exonic | frameshift deletion | c.2263delG |  |
|  |  | *RUNX1* | exonic | stopgain SNV | c.567_568insA |  |
|  |  | *SRSF2* | exonic | nonframeshift insertion | c.284_285insGCC |  |
| 67 | MDS-MLD | *SRSF2* | exonic | nonsynonymous SNV | c.C284G | p.P95R |
| 68 | MDS-MLD | *BCOR* | splicing |  |  |  |
|  |  | *SRSF2* | exonic | nonsynonymous SNV | c.C284G | p.P95R |
|  |  | *ETV6* | exoni | frameshift deletion | c.1244delT |  |
| 69 | MDS-EB | *RUNX1* | exonic | stopgain SNV | c.C908G | p.S303X |
|  |  | *SRSF2* | exonic | nonsynonymous SNV | c.C284A | p.P95H |
|  |  | *ASXL1* | exonic | frameshift deletion | c.1888_1910del |  |
|  |  | *STAG2* | exonic | frameshift deletion | c.1542_1545del |  |
| 70 | MDS-MLD | *U2AF1* | exonic | nonsynonymous SNV | c.A470C | p.Q157P |
| 71 | MDS-EB | *NRAS* | exonic | nonsynonymous SNV | c.G37T | p.G13C |
|  |  | *ASXL1* | exonic | frameshift deletion | c.1917_1927del |  |
|  |  | *EZH2* | exonic | frameshift insertion | c.456_457insA |  |
|  |  | *RUNX1* | exonic | frameshift deletion | c.1202_1221del |  |
|  |  | *STAG2* | exonic | frameshift insertion | c.1642_1643insTT |  |
| 72 | MDS-EB | *RUNX1* | exonic | frameshift insertion | c.1084_1085insC |  |
| 73 | MDS-EB | *DDX41* | splicing |  |  |  |
| 74 | CMML | *CUX1* | exonic | stopgain SNV | c.C3019T | p.R1007X |
|  |  | *SRSF2* | exonic | nonsynonymous SNV | c.C284T | p.P95L |
|  |  | *TET2* | exonic | frameshift insertion | c.4374_4375insA |  |
| 75 | CMML | *ZRSR2* | exonic | stopgain SNV | c.G920A | p.W307X |
|  |  | *TET2* | exonic | frameshift insertion | c.742_743insA |  |
| 76 | CMML | *ASXL1* | exonic | stopgain SNV | c.C2077T | p.R693X |
|  |  | *CBL* | exonic | nonsynonymous SNV | c.T1111C | p.Y371H |
|  |  | *U2AF1* | exonic | nonsynonymous SNV | c.A470C | p.Q157P |
|  |  | *EZH2* | exonic | frameshift insertion | c.716_717insC |  |
| 77 | CMML | *ASXL1* | exonic | stopgain SNV | c.T2324G | p.L775X |
| 78 | MDS-EB | *TP53* | exonic | stopgain SNV | c.C574T | p.Q192X |
| 79 | MDS/MPN-U | *DNMT3A* | exonic | stopgain SNV | c.C1843T | p.Q615X |
|  |  | *TET2* | exonic | stopgain SNV | c.T4607A | p.L1536X |
|  |  | *RUNX1* | exonic | frameshift insertion | c.502_503insT |  |
| 80 | MDS-EB | *DDX41* | exonic | frameshift deletion | c.1586_1587del |  |
| 81 | CMML | *KRAS* | exonic | nonsynonymous SNV | c.G436C | p.A146P |
|  |  | *SRSF2* | exonic | nonsynonymous SNV | c.C284G | p.P95R |
|  |  | *MLL3* | exonic | frameshift deletion | c.4507_4507del |  |
|  |  | *TET2* | splicing |  |  |  |
| 82 | MDS-EB | *ZRSR2* | exonic | stopgain SNV | c.C868T | p.R290X |
| 83 | MDS-MLD | *ZRSR2* | splicing |  |  |  |
| 84 | MDS/MPN-U | *SRSF2* | exonic | nonsynonymous SNV | c.C284A | p.P95H |
|  |  | *TET2* | exonic | stopgain SNV | c.C2491T | p.Q831X |
|  |  | *TET2* | exonic | frameshift deletion | c.1564delT |  |
| 85 | MDS-EB | *BCOR* | exonic | stopgain SNV | c.C4288T | p.Q1430X |
| 86 | CMML | *ZRSR2* | exonic | stopgain SNV | c.C868T | p.R290X |
|  |  | *TET2* | exonic | frameshift deletion | c.5365delG |  |
| 87 | MDS-EB | *CBL* | exonic | nonsynonymous SNV | c.T1111C | p.Y371H |
|  |  | *SETBP1* | exonic | nonsynonymous SNV | c.G2608A | p.G870S |
|  |  | *SRSF2* | exonic | nonsynonymous SNV | c.C284T | p.P95L |
|  |  | *EZH2* | exonic | frameshift deletion | c.1890delG |  |
| 88 | MDS-EB | *SRSF2* | exonic | nonsynonymous SNV | c.C284A | p.P95H |
|  |  | *STAG2* | exonic | frameshift insertion | c.3594_3595insCT |  |
| 89 | MDS-U | *DNMT3A* | exonic | nonsynonymous SNV | c.C2644T | p.R882C |
| 90 | MDS-EB | *DNMT3A* | exonic | nonsynonymous SNV | c.C1903T | p.R635W |
|  |  | *SF3B1* | exonic | nonsynonymous SNV | c.A2098G | p.K700E |
|  |  | *TET2* | splicing |  |  |  |
|  |  | *TET2* | exonic | frameshift insertion | c.2209_2210insC |  |
| 91 | MDS-EB | *NRAS* | exonic | nonsynonymous SNV | c.G35A | p.G12D |
|  |  | *SF3B1* | exonic | nonsynonymous SNV | c.A2098G | p.K700E |
|  |  | *SF3B1* | exonic | nonsynonymous SNV | c.A1876G | p.N626D |
| 92 | CMML | *ETV6* | exonic | stopgain SNV | c.C673T | p.Q225X |
|  |  | *PTPN11* | exonic | nonsynonymous SNV | c.A182G | p.D61G |
|  |  | *SETBP1* | exonic | nonsynonymous SNV | c.G2608A | p.G870S |
|  |  | *SRSF2* | exonic | nonsynonymous SNV | c.C284A | p.P95H |
| 93 | MDS-RS | *SF3B1* | exonic | nonsynonymous SNV | c.A2098G | p.K700E |
| 94 | MDS-EB | *TP53* | exonic | nonsynonymous SNV | c.C827A | p.A276D |
| 95 | MDS-EB | *DNMT3A* | exonic | nonsynonymous SNV | c.G2645A | p.R882H |
|  |  | *SRSF2* | exonic | nonsynonymous SNV | c.C284T | p.P95L |
|  |  | *STAG2* | exonic | stopgain SNV | c.C913T | p.R305X |
|  |  | *TET2* | exonic | nonsynonymous SNV | c.C5756T | p.S1919F |
| 96 | MDS-EB | *TP53* | splicing |  |  |  |
| 97 | MDS-U | *WT1* | exonic | nonsynonymous SNV | c.C1384T | p.R462W |
|  |  | *RUNX1* | exonic | frameshift insertion | c.273_274insGGCGAGCTGGTGCG |  |
| 98 | MDS-MLD | *U2AF1* | exonic | nonsynonymous SNV | c.A470C | p.Q157P |
|  |  | *ASXL1* | exonic | frameshift deletion | c.1744_1760del |  |
|  |  | *CUX1* | exonic | frameshift deletion | c.466_467del |  |
| 99 | MDS-RS | *SF3B1* | exonic | nonsynonymous SNV | c.A2098G | p.K700E |

MDS-SLD = MDS with single lineage dysplasia. MDS-MLD = MDS with multilineage dysplasia. MDS-EB = MDS with excess blasts. MDS-RS = MDS with ring sideroblasts. CMML = Chronic myelomonocytic leukemia. MDS-U = MDS unclassifiable. MDS/MPN-U = MDS/MPN unclassifiable.
