# Supplementary material for: Impact of the number of mutations in survival and response outcomes to hypomethylating agents in patients with myelodysplastic syndromes or myelodysplastic/myeloproliferative neoplasms
Source: Oncotarget. 2018 Jan 3;9(11):9714–27. doi: 10.18632/oncotarget.23882 (PMC5839396; doi:10.18632/oncotarget.23882)
Supplement: Supplementary file 3 [file oncotarget-09-9714-s003.docx]

**Supplementary Table 2: Co-occurrence of mutation patterns and distribution of cytogenetic abnormalties.**

| Table S2. Co-occurrence of mutation patterns and distribution by cytogenetic group | | |
| --- | --- | --- |
|  | Pearson correlation  Coefficient (ρ) | p-value |
| Chr-Y & Chr3r | -0.052 | 0.587 |
| Chr-Y & Del(5q) | 0.218 | 0.022 |
| Chr-Y & Del(7q) | 0.055 | 0.566 |
| Chr-Y & Trisomy 8 | -0.073 | 0.450 |
| Chr-Y & Del11q | -0.030 | 0.758 |
| Chr-Y & Del12 | -0.052 | 0.587 |
| Chr-Y & Del(17p) | 0.140 | 0.145 |
| Chr-Y & i(17q) | -0.030 | 0.758 |
| Chr-Y & Trisomy 19 | 0.297 | 0.002 |
| Chr-Y & Del(20q) | -0.069 | 0.474 |
| Chr-Y & ASXL1 | -0.109 | 0.257 |
| Chr-Y & ATRX | -0.021 | 0.828 |
| Chr-Y & BCOR | -0.052 | 0.587 |
| Chr-Y & CBL | -0.042 | 0.660 |
| Chr-Y & CSF3R | . | . |
| Chr-Y & CUX1 | -0.048 | 0.621 |
| Chr-Y & DDX41 | -0.030 | 0.758 |
| Chr-Y & DNMT3A | -0.048 | 0.621 |
| Chr-Y & ETNK1 | 0.297 | 0.002 |
| Chr-Y & ETV6 | -0.048 | 0.621 |
| Chr-Y & EZH2 | -0.048 | 0.621 |
| Chr-Y & FLT3 | -0.021 | 0.828 |
| Chr-Y & GATA2 | -0.030 | 0.758 |
| Chr-Y & IDH1 | -0.042 | 0.660 |
| Chr-Y & IDH2 | -0.021 | 0.828 |
| Chr-Y & JAK2 | -0.030 | 0.758 |
| Chr-Y & KRAS | -0.037 | 0.705 |
| Chr-Y & MLL3 | -0.021 | 0.828 |
| Chr-Y & NF1 | -0.030 | 0.758 |
| Chr-Y & NRAS | -0.057 | 0.555 |
| Chr-Y & PHF6 | 0.297 | 0.002 |
| Chr-Y & PTPN11 | -0.037 | 0.705 |
| Chr-Y & RUNX1 | -0.090 | 0.350 |
| Chr-Y & SETBP1 | 0.162 | 0.091 |
| Chr-Y & SF3B1 | -0.065 | 0.499 |
| Chr-Y & SRSF2 | -0.023 | 0.811 |
| Chr-Y & STAG2 | -0.069 | 0.474 |
| Chr-Y & TET2 | 0.057 | 0.552 |
| Chr-Y & TP53 | 0.235 | 0.014 |
| Chr-Y & U2AF1 | 0.140 | 0.145 |
| Chr-Y & WT1 | -0.030 | 0.758 |
| Chr-Y & ZRSR2 | 0.083 | 0.390 |
| Chr-Y & Normal karyotype | -0.218 | 0.022 |
| Chr-Y & Complex karyotype | 0.139 | 0.146 |
| Chr3 & Del(5q) | 0.187 | 0.051 |
| Chr3 & Del(7q) | 0.160 | 0.095 |
| Chr3 & Trisomy 8 | 0.053 | 0.580 |
| Chr3 & Del(11q) | -0.033 | 0.735 |
| Chr3 & Del(12) | 0.119 | 0.217 |
| Chr3 & Del(17p) | 0.119 | 0.217 |
| Chr3 & i(17q) | -0.033 | 0.735 |
| Chr3 & Trisomy 19 | -0.033 | 0.735 |
| Chr3 & Del(20q) | -0.076 | 0.430 |
| Chr3 & ASXL1 | -0.020 | 0.836 |
| Chr3 & ATRX | -0.023 | 0.811 |
| Chr3 & BCOR | -0.058 | 0.549 |
| Chr3 & CBL | -0.047 | 0.628 |
| Chr3 & CSF3R | . | . |
| Chr3 & CUX1 | -0.052 | 0.587 |
| Chr3 & DDX41 | -0.033 | 0.735 |
| Chr3 & DNMT3A | -0.052 | 0.587 |
| Chr3 & ETNK1 | -0.033 | 0.735 |
| Chr3 & ETV6 | -0.052 | 0.587 |
| Chr3 & EZH2 | -0.052 | 0.587 |
| Chr3 & FLT3 | -0.023 | 0.811 |
| Chr3 & GATA2 | -0.033 | 0.735 |
| Chr3 & IDH1 | -0.047 | 0.628 |
| Chr3 & IDH2 | -0.023 | 0.811 |
| Chr3 & JAK2 | -0.033 | 0.735 |
| Chr3 & KRAS | 0.206 | 0.031 |
| Chr3 & MLL3 | -0.023 | 0.811 |
| Chr3 & NF1 | -0.033 | 0.735 |
| Chr3 & NRAS | -0.063 | 0.516 |
| Chr3 & PHF6 | -0.033 | 0.735 |
| Chr3 & PTPN11 | -0.040 | 0.677 |
| Chr3 & RUNX1 | -0.099 | 0.303 |
| Chr3 & SETBP1 | -0.052 | 0.587 |
| Chr3 & SF3B1 | 0.220 | 0.021 |
| Chr3 & SRSF2 | -0.137 | 0.154 |
| Chr3 & STAG2 | -0.076 | 0.430 |
| Chr3 & TET2 | -0.150 | 0.117 |
| Chr3 & TP53 | 0.203 | 0.034 |
| Chr3 & U2AF1 | -0.058 | 0.549 |
| Chr3 & WT1 | -0.033 | 0.735 |
| Chr3 & ZRSR2 | -0.076 | 0.430 |
| Chr3 & Normal karyotype | -0.240 | 0.011 |
| Chr3 & Complex karyotype | 0.218 | 0.022 |
| Del(5q) & Del(7q) | 0.441 | 0.000 |
| Del(5q) & Trisomy 8 | 0.192 | 0.045 |
| Del(5q) & Del(11q) | -0.045 | 0.638 |
| Del(5q) & Del(12) | 0.454 | 0.000 |
| Del(5q) & Del(17p) | 0.187 | 0.051 |
| Del(5q) & i(17q) | -0.045 | 0.638 |
| Del(5q) & Trisomy 19 | 0.408 | 0.000 |
| Del(5q) & Del(20q) | 0.000 | 1.000 |
| Del(5q) & ASXL1 | -0.167 | 0.082 |
| Del(5q) & ATRX | -0.032 | 0.741 |
| Del(5q) & BCOR | -0.080 | 0.406 |
| Del(5q) & CBL | -0.065 | 0.502 |
| Del(5q) & CSF3R | . | . |
| Del(5q) & CUX1 | -0.073 | 0.450 |
| Del(5q) & DDX41 | -0.045 | 0.638 |
| Del(5q) & DNMT3A | -0.073 | 0.450 |
| Del(5q) & ETNK1 | -0.045 | 0.638 |
| Del(5q) & ETV6 | -0.073 | 0.450 |
| Del(5q) & EZH2 | -0.073 | 0.450 |
| Del(5q) & FLT3 | -0.032 | 0.741 |
| Del(5q) & GATA2 | -0.045 | 0.638 |
| Del(5q) & IDH1 | -0.065 | 0.502 |
| Del(5q) & IDH2 | -0.032 | 0.741 |
| Del(5q) & JAK2 | -0.045 | 0.638 |
| Del(5q) & KRAS | -0.056 | 0.562 |
| Del(5q) & MLL3 | -0.032 | 0.741 |
| Del(5q) & NF1 | -0.045 | 0.638 |
| Del(5q) & NRAS | -0.087 | 0.367 |
| Del(5q) & PHF6 | -0.045 | 0.638 |
| Del(5q) & PTPN11 | -0.056 | 0.562 |
| Del(5q) & RUNX1 | -0.138 | 0.152 |
| Del(5q) & SETBP1 | -0.073 | 0.450 |
| Del(5q) & SF3B1 | -0.100 | 0.301 |
| Del(5q) & SRSF2 | -0.120 | 0.213 |
| Del(5q) & STAG2 | -0.105 | 0.273 |
| Del(5q) & TET2 | -0.141 | 0.140 |
| Del(5q) & TP53 | 0.527 | 0.000 |
| Del(5q) & U2AF1 | -0.080 | 0.406 |
| Del(5q) & WT1 | -0.045 | 0.638 |
| Del(5q) & ZRSR2 | -0.000 | 1.000 |
| Del(5q) & Normal karyotype | -0.333 | 0.000 |
| Del(5q) & Complex karyotype | 0.590 | 0.000 |
| Del(7q) & Trisomy 8 | 0.066 | 0.495 |
| Del(7q) & Del(11q) | -0.050 | 0.605 |
| Del(7q) & Del(12) | 0.408 | 0.000 |
| Del(7q) & Del(17p) | 0.284 | 0.003 |
| Del(7q) & i(17q) | -0.050 | 0.605 |
| Del(7q) & Trisomy 19 | 0.161 | 0.093 |
| Del(7q) & Del(20q) | 0.178 | 0.063 |
| Del(7q) & ASXL1 | -0.183 | 0.056 |
| Del(7q) & ATRX | -0.035 | 0.716 |
| Del(7q) & BCOR | 0.036 | 0.708 |
| Del(7q) & CBL | -0.071 | 0.460 |
| Del(7q) & CSF3R | . | . |
| Del(7q) & CUX1 | -0.080 | 0.407 |
| Del(7q) & DDX41 | -0.050 | 0.605 |
| Del(7q) & DNMT3A | 0.055 | 0.566 |
| Del(7q) & ETNK1 | -0.050 | 0.605 |
| Del(7q) & ETV6 | 0.055 | 0.566 |
| Del(7q) & EZH2 | -0.080 | 0.407 |
| Del(7q) & FLT3 | 0.262 | 0.006 |
| Del(7q) & GATA2 | -0.050 | 0.605 |
| Del(7q) & IDH1 | -0.071 | 0.460 |
| Del(7q) & IDH2 | -0.035 | 0.716 |
| Del(7q) & JAK2 | -0.050 | 0.605 |
| Del(7q) & KRAS | 0.112 | 0.246 |
| Del(7q) & MLL3 | -0.035 | 0.716 |
| Del(7q) & NF1 | -0.050 | 0.605 |
| Del(7q) & NRAS | -0.095 | 0.321 |
| Del(7q) & PHF6 | -0.050 | 0.605 |
| Del(7q) & PTPN11 | -0.061 | 0.525 |
| Del(7q) & RUNX1 | -0.071 | 0.460 |
| Del(7q) & SETBP1 | -0.080 | 0.407 |
| Del(7q) & SF3B1 | -0.109 | 0.256 |
| Del(7q) & SRSF2 | -0.078 | 0.418 |
| Del(7q) & STAG2 | -0.116 | 0.228 |
| Del(7q) & TET2 | -0.104 | 0.279 |
| Del(7q) & TP53 | 0.472 | 0.000 |
| Del(7q) & U2AF1 | -0.088 | 0.361 |
| Del(7q) & WT1 | -0.050 | 0.605 |
| Del(7q) & ZRSR2 | -0.116 | 0.228 |
| Del(7q) & Normal karyotype | -0.366 | 0.000 |
| Del(7q) & Complex karyotype | 0.523 | 0.000 |
| Trisomy 8 & Del(11q) | -0.045 | 0.638 |
| Trisomy 8 & Del(12) | 0.053 | 0.580 |
| Trisomy 8 & Del(17p) | 0.053 | 0.580 |
| Trisomy 8 & i(17q) | -0.045 | 0.638 |
| Trisomy 8 & Trisomy 19 | -0.045 | 0.638 |
| Trisomy 8 & Del(20q) | -0.105 | 0.273 |
| Trisomy 8 & ASXL1 | -0.015 | 0.875 |
| Trisomy 8 & ATRX | -0.032 | 0.741 |
| Trisomy 8 & BCOR | 0.187 | 0.051 |
| Trisomy 8 & CBL | 0.097 | 0.313 |
| Trisomy 8 & CSF3R | . | . |
| Trisomy 8 & CUX1 | -0.073 | 0.450 |
| Trisomy 8 & DDX41 | -0.045 | 0.638 |
| Trisomy 8 & DNMT3A | -0.073 | 0.450 |
| Trisomy 8 & ETNK1 | -0.045 | 0.638 |
| Trisomy 8 & ETV6 | 0.073 | 0.450 |
| Trisomy 8 & EZH2 | -0.073 | 0.450 |
| Trisomy 8 & FLT3 | -0.032 | 0.741 |
| Trisomy 8 & GATA2 | -0.045 | 0.638 |
| Trisomy 8 & IDH1 | -0.065 | 0.502 |
| Trisomy 8 & IDH2 | -0.032 | 0.741 |
| Trisomy 8 & JAK2 | -0.045 | 0.638 |
| Trisomy 8 & KRAS | -0.056 | 0.562 |
| Trisomy 8 & MLL3 | -0.032 | 0.741 |
| Trisomy 8 & NF1 | -0.045 | 0.638 |
| Trisomy 8 & NRAS | -0.087 | 0.367 |
| Trisomy 8 & PHF6 | -0.045 | 0.638 |
| Trisomy 8 & PTPN11 | -0.056 | 0.562 |
| Trisomy 8 & RUNX1 | -0.052 | 0.593 |
| Trisomy 8 & SETBP1 | -0.073 | 0.450 |
| Trisomy 8 & SF3B1 | -0.100 | 0.301 |
| Trisomy 8 & SRSF2 | -0.190 | 0.047 |
| Trisomy 8 & STAG2 | -0.000 | 1.000 |
| Trisomy 8 & TET2 | -0.074 | 0.442 |
| Trisomy 8 & TP53 | -0.000 | 1.000 |
| Trisomy 8 & U2AF1 | 0.053 | 0.580 |
| Trisomy 8 & WT1 | -0.045 | 0.638 |
| Trisomy 8 & ZRSR2 | -0.000 | 1.000 |
| Trisomy 8 & Normal karyotype | -0.333 | 0.000 |
| Trisomy 8 & Complex karyotype | 0.016 | 0.865 |
| Del(11q) & Del(12) | -0.033 | 0.735 |
| Del(11q) & Del(17p) | -0.033 | 0.735 |
| Del(11q) & i(17q) | -0.019 | 0.848 |
| Del(11q) & Trisomy 19 | -0.019 | 0.848 |
| Del(11q) & Del(20q) | -0.043 | 0.655 |
| Del(11q) & ASXL1 | -0.068 | 0.480 |
| Del(11q) & ATRX | -0.013 | 0.892 |
| Del(11q) & BCOR | -0.033 | 0.735 |
| Del(11q) & CBL | -0.026 | 0.784 |
| Del(11q) & CSF3R | . | . |
| Del(11q) & CUX1 | -0.030 | 0.758 |
| Del(11q) & DDX41 | -0.019 | 0.848 |
| Del(11q) & DNMT3A | -0.030 | 0.758 |
| Del(11q) & ETNK1 | -0.019 | 0.848 |
| Del(11q) & ETV6 | -0.030 | 0.758 |
| Del(11q) & EZH2 | -0.030 | 0.758 |
| Del(11q) & FLT3 | -0.013 | 0.892 |
| Del(11q) & GATA2 | -0.019 | 0.848 |
| Del(11q) & IDH1 | -0.026 | 0.784 |
| Del(11q) & IDH2 | -0.013 | 0.892 |
| Del(11q) & JAK2 | -0.019 | 0.848 |
| Del(11q) & KRAS | -0.023 | 0.813 |
| Del(11q) & MLL3 | -0.013 | 0.892 |
| Del(11q) & NF1 | -0.019 | 0.848 |
| Del(11q) & NRAS | -0.035 | 0.713 |
| Del(11q) & PHF6 | -0.019 | 0.848 |
| Del(11q) & PTPN11 | -0.023 | 0.813 |
| Del(11q) & RUNX1 | -0.056 | 0.560 |
| Del(11q) & SETBP1 | -0.030 | 0.758 |
| Del(11q) & SF3B1 | -0.041 | 0.673 |
| Del(11q) & SRSF2 | -0.078 | 0.420 |
| Del(11q) & STAG2 | -0.043 | 0.655 |
| Del(11q) & TET2 | -0.085 | 0.376 |
| Del(11q) & TP53 | -0.043 | 0.655 |
| Del(11q) & U2AF1 | -0.033 | 0.735 |
| Del(11q) & WT1 | -0.019 | 0.848 |
| Del(11q) & ZRSR2 | -0.043 | 0.655 |
| Del(11q) & Normal karyotype | -0.136 | 0.156 |
| Del(11q) & Complex karyotype | -0.060 | 0.532 |
| Del(12) & Del(17p) | 0.119 | 0.217 |
| Del(12) & i(17q) | -0.033 | 0.735 |
| Del(12) & Trisomy 19 | 0.267 | 0.005 |
| Del(12) & Del(20q) | 0.063 | 0.511 |
| Del(12) & ASXL1 | -0.120 | 0.211 |
| Del(12) & ATRX | -0.023 | 0.811 |
| Del(12) & BCOR | 0.119 | 0.217 |
| Del(12) & CBL | -0.047 | 0.628 |
| Del(12) & CSF3R | . | . |
| Del(12) & CUX1 | -0.052 | 0.587 |
| Del(12) & DDX41 | -0.033 | 0.735 |
| Del(12) & DNMT3A | -0.052 | 0.587 |
| Del(12) & ETNK1 | -0.033 | 0.735 |
| Del(12) & ETV6 | -0.052 | 0.587 |
| Del(12) & EZH2 | -0.052 | 0.587 |
| Del(12) & FLT3 | -0.023 | 0.811 |
| Del(12) & GATA2 | -0.033 | 0.735 |
| Del(12) & IDH1 | -0.047 | 0.628 |
| Del(12) & IDH2 | -0.023 | 0.811 |
| Del(12) & JAK2 | -0.033 | 0.735 |
| Del(12) & KRAS | -0.040 | 0.677 |
| Del(12) & MLL3 | -0.023 | 0.811 |
| Del(12) & NF1 | -0.033 | 0.735 |
| Del(12) & NRAS | -0.063 | 0.516 |
| Del(12) & PHF6 | -0.033 | 0.735 |
| Del(12) & PTPN11 | -0.040 | 0.677 |
| Del(12) & RUNX1 | -0.099 | 0.303 |
| Del(12) & SETBP1 | -0.052 | 0.587 |
| Del(12) & SF3B1 | -0.072 | 0.457 |
| Del(12) & SRSF2 | -0.137 | 0.154 |
| Del(12) & STAG2 | -0.076 | 0.430 |
| Del(12) & TET2 | -0.150 | 0.117 |
| Del(12) & TP53 | 0.342 | 0.000 |
| Del(12) & U2AF1 | -0.058 | 0.549 |
| Del(12) & WT1 | -0.033 | 0.735 |
| Del(12) & ZRSR2 | -0.076 | 0.430 |
| Del(12) & Normal karyotype | -0.240 | 0.011 |
| Del(12) & Complex karyotype | 0.543 | 0.000 |
| Del(17p) & i(17q) | 0.267 | 0.005 |
| Del(17p) & Trisomy 19 | 0.267 | 0.005 |
| Del(17p) & Del(20q) | 0.203 | 0.034 |
| Del(17p) & ASXL1 | -0.120 | 0.211 |
| Del(17p) & ATRX | -0.023 | 0.811 |
| Del(17p) & BCOR | -0.058 | 0.549 |
| Del(17p) & CBL | -0.047 | 0.628 |
| Del(17p) & CSF3R | . | . |
| Del(17p) & CUX1 | -0.052 | 0.587 |
| Del(17p) & DDX41 | -0.033 | 0.735 |
| Del(17p) & DNMT3A | -0.052 | 0.587 |
| Del(17p) & ETNK1 | -0.033 | 0.735 |
| Del(17p) & ETV6 | 0.140 | 0.145 |
| Del(17p) & EZH2 | -0.052 | 0.587 |
| Del(17p) & FLT3 | -0.023 | 0.811 |
| Del(17p) & GATA2 | -0.033 | 0.735 |
| Del(17p) & IDH1 | -0.047 | 0.628 |
| Del(17p) & IDH2 | -0.023 | 0.811 |
| Del(17p) & JAK2 | -0.033 | 0.735 |
| Del(17p) & KRAS | -0.040 | 0.677 |
| Del(17p) & MLL3 | -0.023 | 0.811 |
| Del(17p) & NF1 | -0.033 | 0.735 |
| Del(17p) & NRAS | -0.063 | 0.516 |
| Del(17p) & PHF6 | -0.033 | 0.735 |
| Del(17p) & PTPN11 | 0.206 | 0.031 |
| Del(17p) & RUNX1 | 0.014 | 0.881 |
| Del(17p) & SETBP1 | 0.140 | 0.145 |
| Del(17p) & SF3B1 | -0.072 | 0.457 |
| Del(17p) & SRSF2 | -0.044 | 0.648 |
| Del(17p) & STAG2 | -0.076 | 0.430 |
| Del(17p) & TET2 | -0.150 | 0.117 |
| Del(17p) & TP53 | 0.481 | 0.000 |
| Del(17p) & U2AF1 | -0.058 | 0.549 |
| Del(17p) & WT1 | -0.033 | 0.735 |
| Del(17p) & ZRSR2 | -0.076 | 0.430 |
| Del(17p) & Normal karyotype | -0.240 | 0.011 |
| Del(17p) & Complex karyotype | 0.435 | 0.000 |
| i(17q) & Trisomy 19 | -0.019 | 0.848 |
| i(17q) & Del(20q) | -0.043 | 0.655 |
| i(17q) & ASXL1 | 0.102 | 0.289 |
| i(17q) & ATRX | -0.013 | 0.892 |
| i(17q) & BCOR | -0.033 | 0.735 |
| i(17q) & CBL | -0.026 | 0.784 |
| i(17q) & CSF3R | . | . |
| i(17q) & CUX1 | -0.030 | 0.758 |
| i(17q) & DDX41 | -0.019 | 0.848 |
| i(17q) & DNMT3A | -0.030 | 0.758 |
| i(17q) & ETNK1 | -0.019 | 0.848 |
| i(17q) & ETV6 | 0.624 | 0.000 |
| i(17q) & EZH2 | -0.030 | 0.758 |
| i(17q) & FLT3 | -0.013 | 0.892 |
| i(17q) & GATA2 | -0.019 | 0.848 |
| i(17q) & IDH1 | -0.026 | 0.784 |
| i(17q) & IDH2 | -0.013 | 0.892 |
| i(17q) & JAK2 | -0.019 | 0.848 |
| i(17q) & KRAS | -0.023 | 0.813 |
| i(17q) & MLL3 | -0.013 | 0.892 |
| i(17q) & NF1 | -0.019 | 0.848 |
| i(17q) & NRAS | 0.243 | 0.010 |
| i(17q) & PHF6 | -0.019 | 0.848 |
| i(17q) & PTPN11 | 0.395 | 0.000 |
| i(17q) & RUNX1 | -0.056 | 0.560 |
| i(17q) & SETBP1 | 0.624 | 0.000 |
| i(17q) & SF3B1 | -0.041 | 0.673 |
| i(17q) & SRSF2 | 0.239 | 0.012 |
| i(17q) & STAG2 | -0.043 | 0.655 |
| i(17q) & TET2 | -0.085 | 0.376 |
| i(17q) & TP53 | -0.043 | 0.655 |
| i(17q) & U2AF1 | -0.033 | 0.735 |
| i(17q) & WT1 | -0.019 | 0.848 |
| i(17q) & ZRSR2 | -0.043 | 0.655 |
| i(17q) & Normal karyotype | -0.136 | 0.156 |
| i(17q) & Complex karyotype | -0.060 | 0.532 |
| Trisomy 19 & Del(20q) | 0.194 | 0.043 |
| Trisomy 19 & ASXL1 | -0.068 | 0.480 |
| Trisomy 19 & ATRX | -0.013 | 0.892 |
| Trisomy 19 & BCOR | -0.033 | 0.735 |
| Trisomy 19 & CBL | -0.026 | 0.784 |
| Trisomy 19 & CSF3R | . | . |
| Trisomy 19 & CUX1 | -0.030 | 0.758 |
| Trisomy 19 & DDX41 | -0.019 | 0.848 |
| Trisomy 19 & DNMT3A | -0.030 | 0.758 |
| Trisomy 19 & ETNK1 | -0.019 | 0.848 |
| Trisomy 19 & ETV6 | -0.030 | 0.758 |
| Trisomy 19 & EZH2 | -0.030 | 0.758 |
| Trisomy 19 & FLT3 | -0.013 | 0.892 |
| Trisomy 19 & GATA2 | -0.019 | 0.848 |
| Trisomy 19 & IDH1 | -0.026 | 0.784 |
| Trisomy 19 & IDH2 | -0.013 | 0.892 |
| Trisomy 19 & JAK2 | -0.019 | 0.848 |
| Trisomy 19 & KRAS | -0.023 | 0.813 |
| Trisomy 19 & MLL3 | -0.013 | 0.892 |
| Trisomy 19 & NF1 | -0.019 | 0.848 |
| Trisomy 19 & NRAS | -0.035 | 0.713 |
| Trisomy 19 & PHF6 | -0.019 | 0.848 |
| Trisomy 19 & PTPN11 | -0.023 | 0.813 |
| Trisomy 19 & RUNX1 | -0.056 | 0.560 |
| Trisomy 19 & SETBP1 | -0.030 | 0.758 |
| Trisomy 19 & SF3B1 | -0.041 | 0.673 |
| Trisomy 19 & SRSF2 | -0.078 | 0.420 |
| Trisomy 19 & STAG2 | -0.043 | 0.655 |
| Trisomy 19 & TET2 | -0.085 | 0.376 |
| Trisomy 19 & TP53 | 0.430 | 0.000 |
| Trisomy 19 & U2AF1 | -0.033 | 0.735 |
| Trisomy 19 & WT1 | -0.019 | 0.848 |
| Trisomy 19 & ZRSR2 | -0.043 | 0.655 |
| Trisomy 19 & Normal karyotype | -0.136 | 0.156 |
| Trisomy 19 & Complex karyotype | 0.308 | 0.001 |
| Del(20q) & ASXL1 | -0.158 | 0.099 |
| Del(20q) & ATRX | -0.030 | 0.753 |
| Del(20q) & BCOR | -0.076 | 0.430 |
| Del(20q) & CBL | 0.108 | 0.264 |
| Del(20q) & CSF3R | . | . |
| Del(20q) & CUX1 | 0.235 | 0.014 |
| Del(20q) & DDX41 | -0.043 | 0.655 |
| Del(20q) & DNMT3A | -0.069 | 0.474 |
| Del(20q) & ETNK1 | -0.043 | 0.655 |
| Del(20q) & ETV6 | 0.083 | 0.390 |
| Del(20q) & EZH2 | -0.069 | 0.474 |
| Del(20q) & FLT3 | -0.030 | 0.753 |
| Del(20q) & GATA2 | -0.043 | 0.655 |
| Del(20q) & IDH1 | -0.061 | 0.524 |
| Del(20q) & IDH2 | 0.303 | 0.001 |
| Del(20q) & JAK2 | -0.043 | 0.655 |
| Del(20q) & KRAS | -0.053 | 0.583 |
| Del(20q) & MLL3 | -0.030 | 0.753 |
| Del(20q) & NF1 | -0.043 | 0.655 |
| Del(20q) & NRAS | -0.082 | 0.392 |
| Del(20q) & PHF6 | 0.194 | 0.043 |
| Del(20q) & PTPN11 | -0.053 | 0.583 |
| Del(20q) & RUNX1 | 0.049 | 0.612 |
| Del(20q) & SETBP1 | 0.083 | 0.390 |
| Del(20q) & SF3B1 | -0.094 | 0.327 |
| Del(20q) & SRSF2 | 0.114 | 0.238 |
| Del(20q) & STAG2 | -0.100 | 0.299 |
| Del(20q) & TET2 | 0.013 | 0.895 |
| Del(20q) & TP53 | 0.230 | 0.016 |
| Del(20q) & U2AF1 | -0.076 | 0.430 |
| Del(20q) & WT1 | -0.043 | 0.655 |
| Del(20q) & ZRSR2 | 0.010 | 0.917 |
| Del(20q) & Normal karyotype | -0.316 | 0.001 |
| Del(20q) & Complex karyotype | 0.202 | 0.034 |
| ASXL1 & ATRX | 0.187 | 0.046 |
| ASXL1 & BCOR | -0.118 | 0.209 |
| ASXL1 & CBL | 0.023 | 0.809 |
| ASXL1 & CSF3R | . | . |
| ASXL1 & CUX1 | -0.001 | 0.992 |
| ASXL1 & DDX41 | -0.067 | 0.478 |
| ASXL1 & DNMT3A | -0.129 | 0.173 |
| ASXL1 & ETNK1 | 0.099 | 0.293 |
| ASXL1 & ETV6 | -0.001 | 0.992 |
| ASXL1 & EZH2 | 0.319 | 0.001 |
| ASXL1 & FLT3 | -0.047 | 0.617 |
| ASXL1 & GATA2 | 0.266 | 0.004 |
| ASXL1 & IDH1 | 0.023 | 0.809 |
| ASXL1 & IDH2 | -0.047 | 0.617 |
| ASXL1 & JAK2 | -0.067 | 0.478 |
| ASXL1 & KRAS | 0.054 | 0.569 |
| ASXL1 & MLL3 | -0.067 | 0.478 |
| ASXL1 & NF1 | -0.067 | 0.478 |
| ASXL1 & NRAS | 0.033 | 0.727 |
| ASXL1 & PHF6 | -0.067 | 0.478 |
| ASXL1 & PTPN11 | 0.054 | 0.569 |
| ASXL1 & RUNX1 | 0.112 | 0.238 |
| ASXL1 & SETBP1 | -0.001 | 0.992 |
| ASXL1 & SF3B1 | -0.147 | 0.118 |
| ASXL1 & SRSF2 | 0.080 | 0.399 |
| ASXL1 & STAG2 | 0.153 | 0.104 |
| ASXL1 & TET2 | 0.113 | 0.232 |
| ASXL1 & TP53 | -0.001 | 0.989 |
| ASXL1 & U2AF1 | 0.077 | 0.414 |
| ASXL1 & WT1 | -0.067 | 0.478 |
| ASXL1 & ZRSR2 | -0.001 | 0.989 |
| ASXL1 & Normal karyotype | 0.182 | 0.057 |
| ASXL1 & Complex karyotype | -0.221 | 0.020 |
| ATRX & BCOR | -0.022 | 0.815 |
| ATRX & CBL | -0.018 | 0.850 |
| ATRX & CSF3R | . | . |
| ATRX & CUX1 | -0.020 | 0.832 |
| ATRX & DDX41 | -0.013 | 0.894 |
| ATRX & DNMT3A | -0.024 | 0.799 |
| ATRX & ETNK1 | -0.013 | 0.894 |
| ATRX & ETV6 | -0.020 | 0.832 |
| ATRX & EZH2 | -0.020 | 0.832 |
| ATRX & FLT3 | -0.009 | 0.926 |
| ATRX & GATA2 | 0.704 | 0.000 |
| ATRX & IDH1 | -0.018 | 0.850 |
| ATRX & IDH2 | -0.009 | 0.926 |
| ATRX & JAK2 | -0.013 | 0.894 |
| ATRX & KRAS | -0.015 | 0.870 |
| ATRX & MLL3 | -0.013 | 0.894 |
| ATRX & NF1 | -0.013 | 0.894 |
| ATRX & NRAS | -0.026 | 0.785 |
| ATRX & PHF6 | -0.013 | 0.894 |
| ATRX & PTPN11 | 0.572 | 0.000 |
| ATRX & RUNX1 | 0.233 | 0.013 |
| ATRX & SETBP1 | -0.020 | 0.832 |
| ATRX & SF3B1 | -0.028 | 0.771 |
| ATRX & SRSF2 | 0.169 | 0.072 |
| ATRX & STAG2 | 0.303 | 0.001 |
| ATRX & TET2 | -0.060 | 0.526 |
| ATRX & TP53 | -0.029 | 0.758 |
| ATRX & U2AF1 | -0.022 | 0.815 |
| ATRX & WT1 | -0.013 | 0.894 |
| ATRX & ZRSR2 | -0.029 | 0.758 |
| ATRX & Normal karyotype | -0.096 | 0.320 |
| ATRX & Complex karyotype | -0.042 | 0.660 |
| CBL & CSF3R | . | . |
| CBL & CUX1 | -0.041 | 0.666 |
| CBL & DDX41 | -0.025 | 0.788 |
| CBL & DNMT3A | -0.049 | 0.606 |
| CBL & ETNK1 | -0.025 | 0.788 |
| CBL & ETV6 | -0.041 | 0.666 |
| CBL & EZH2 | 0.425 | 0.000 |
| CBL & FLT3 | -0.018 | 0.850 |
| CBL & GATA2 | -0.025 | 0.788 |
| CBL & IDH1 | -0.036 | 0.701 |
| CBL & IDH2 | 0.493 | 0.000 |
| CBL & JAK2 | -0.025 | 0.788 |
| CBL & KRAS | -0.031 | 0.741 |
| CBL & MLL3 | -0.025 | 0.788 |
| CBL & NF1 | -0.025 | 0.788 |
| CBL & NRAS | -0.052 | 0.580 |
| CBL & PHF6 | -0.025 | 0.788 |
| CBL & PTPN11 | -0.031 | 0.741 |
| CBL & RUNX1 | -0.077 | 0.415 |
| CBL & SETBP1 | 0.425 | 0.000 |
| CBL & SF3B1 | -0.056 | 0.555 |
| CBL & SRSF2 | 0.118 | 0.211 |
| CBL & STAG2 | -0.059 | 0.532 |
| CBL & TET2 | -0.122 | 0.197 |
| CBL & TP53 | -0.059 | 0.532 |
| CBL & U2AF1 | 0.169 | 0.073 |
| CBL & WT1 | -0.025 | 0.788 |
| CBL & ZRSR2 | -0.059 | 0.532 |
| CBL & Normal karyotype | 0.000 | 1.000 |
| CBL & Complex karyotype | -0.086 | 0.372 |
| CSF3R & CUX1 | . | . |
| CSF3R & DDX41 | . | . |
| CSF3R & DNMT3A | . | . |
| CSF3R & ETNK1 | . | . |
| CSF3R & ETV6 | . | . |
| CSF3R & EZH2 | . | . |
| CSF3R & FLT3 | . | . |
| CSF3R & GATA2 | . | . |
| CSF3R & IDH1 | . | . |
| CSF3R & IDH2 | . | . |
| CSF3R & JAK2 | . | . |
| CSF3R & KRAS | . | . |
| CSF3R & MLL3 | . | . |
| CSF3R & NF1 | . | . |
| CSF3R & NRAS | . | . |
| CSF3R & PHF6 | . | . |
| CSF3R & PTPN11 | . | . |
| CSF3R & RUNX1 | . | . |
| CSF3R & SETBP1 | . | . |
| CSF3R & SF3B1 | . | . |
| CSF3R & SRSF2 | . | . |
| CSF3R & STAG2 | . | . |
| CSF3R & TET2 | . | . |
| CSF3R & TP53 | . | . |
| CSF3R & U2AF1 | . | . |
| CSF3R & WT1 | . | . |
| CSF3R & ZRSR2 | . | . |
| CSF3R & Normal karyotype | . | . |
| CSF3R & Complex karyotype | . | . |
| CUX1 & DDX41 | -0.029 | 0.762 |
| CUX1 & DNMT3A | -0.055 | 0.563 |
| CUX1 & ETNK1 | -0.029 | 0.762 |
| CUX1 & ETV6 | 0.163 | 0.083 |
| CUX1 & EZH2 | -0.046 | 0.628 |
| CUX1 & FLT3 | -0.020 | 0.832 |
| CUX1 & GATA2 | -0.029 | 0.762 |
| CUX1 & IDH1 | 0.192 | 0.041 |
| CUX1 & IDH2 | -0.020 | 0.832 |
| CUX1 & JAK2 | -0.029 | 0.762 |
| CUX1 & KRAS | -0.035 | 0.710 |
| CUX1 & MLL3 | -0.029 | 0.762 |
| CUX1 & NF1 | 0.298 | 0.001 |
| CUX1 & NRAS | -0.059 | 0.534 |
| CUX1 & PHF6 | -0.029 | 0.762 |
| CUX1 & PTPN11 | -0.035 | 0.710 |
| CUX1 & RUNX1 | -0.087 | 0.360 |
| CUX1 & SETBP1 | -0.046 | 0.628 |
| CUX1 & SF3B1 | -0.063 | 0.507 |
| CUX1 & SRSF2 | 0.284 | 0.002 |
| CUX1 & STAG2 | -0.066 | 0.483 |
| CUX1 & TET2 | 0.052 | 0.581 |
| CUX1 & TP53 | -0.066 | 0.483 |
| CUX1 & U2AF1 | 0.141 | 0.134 |
| CUX1 & WT1 | -0.029 | 0.762 |
| CUX1 & ZRSR2 | -0.066 | 0.483 |
| CUX1 & Normal karyotype | -0.044 | 0.651 |
| CUX1 & Complex karyotype | -0.097 | 0.316 |
| DDX41 & DNMT3A | -0.034 | 0.718 |
| DDX41 & ETNK1 | -0.018 | 0.850 |
| DDX41 & ETV6 | -0.029 | 0.762 |
| DDX41 & EZH2 | -0.029 | 0.762 |
| DDX41 & FLT3 | -0.013 | 0.894 |
| DDX41 & GATA2 | -0.018 | 0.850 |
| DDX41 & IDH1 | -0.025 | 0.788 |
| DDX41 & IDH2 | -0.013 | 0.894 |
| DDX41 & JAK2 | -0.018 | 0.850 |
| DDX41 & KRAS | -0.022 | 0.817 |
| DDX41 & MLL3 | -0.018 | 0.850 |
| DDX41 & NF1 | -0.018 | 0.850 |
| DDX41 & NRAS | -0.037 | 0.698 |
| DDX41 & PHF6 | -0.018 | 0.850 |
| DDX41 & PTPN11 | -0.022 | 0.817 |
| DDX41 & RUNX1 | -0.054 | 0.568 |
| DDX41 & SETBP1 | -0.029 | 0.762 |
| DDX41 & SF3B1 | -0.039 | 0.679 |
| DDX41 & SRSF2 | -0.074 | 0.431 |
| DDX41 & STAG2 | -0.041 | 0.662 |
| DDX41 & TET2 | -0.085 | 0.367 |
| DDX41 & TP53 | -0.041 | 0.662 |
| DDX41 & U2AF1 | -0.031 | 0.739 |
| DDX41 & WT1 | -0.018 | 0.850 |
| DDX41 & ZRSR2 | -0.041 | 0.662 |
| DDX41 & Normal karyotype | 0.136 | 0.156 |
| DDX41 & Complex karyotype | -0.060 | 0.532 |
| DNMT3A & ETNK1 | -0.034 | 0.718 |
| DNMT3A & ETV6 | -0.055 | 0.563 |
| DNMT3A & EZH2 | -0.055 | 0.563 |
| DNMT3A & FLT3 | 0.368 | 0.000 |
| DNMT3A & GATA2 | -0.034 | 0.718 |
| DNMT3A & IDH1 | -0.049 | 0.606 |
| DNMT3A & IDH2 | -0.024 | 0.799 |
| DNMT3A & JAK2 | -0.034 | 0.718 |
| DNMT3A & KRAS | -0.042 | 0.657 |
| DNMT3A & MLL3 | 0.244 | 0.009 |
| DNMT3A & NF1 | -0.034 | 0.718 |
| DNMT3A & NRAS | 0.073 | 0.442 |
| DNMT3A & PHF6 | -0.034 | 0.718 |
| DNMT3A & PTPN11 | -0.042 | 0.657 |
| DNMT3A & RUNX1 | 0.212 | 0.023 |
| DNMT3A & SETBP1 | -0.055 | 0.563 |
| DNMT3A & SF3B1 | 0.061 | 0.522 |
| DNMT3A & SRSF2 | -0.057 | 0.550 |
| DNMT3A & STAG2 | 0.179 | 0.057 |
| DNMT3A & TET2 | 0.240 | 0.010 |
| DNMT3A & TP53 | -0.079 | 0.402 |
| DNMT3A & U2AF1 | -0.060 | 0.524 |
| DNMT3A & WT1 | -0.034 | 0.718 |
| DNMT3A & ZRSR2 | -0.079 | 0.402 |
| DNMT3A & Normal karyotype | 0.131 | 0.173 |
| DNMT3A & Complex karyotype | -0.097 | 0.316 |
| ETNK1 & ETV6 | -0.029 | 0.762 |
| ETNK1 & EZH2 | -0.029 | 0.762 |
| ETNK1 & FLT3 | -0.013 | 0.894 |
| ETNK1 & GATA2 | 0.491 | 0.000 |
| ETNK1 & IDH1 | -0.025 | 0.788 |
| ETNK1 & IDH2 | -0.013 | 0.894 |
| ETNK1 & JAK2 | -0.018 | 0.850 |
| ETNK1 & KRAS | -0.022 | 0.817 |
| ETNK1 & MLL3 | -0.018 | 0.850 |
| ETNK1 & NF1 | -0.018 | 0.850 |
| ETNK1 & NRAS | -0.037 | 0.698 |
| ETNK1 & PHF6 | -0.018 | 0.850 |
| ETNK1 & PTPN11 | -0.022 | 0.817 |
| ETNK1 & RUNX1 | -0.054 | 0.568 |
| ETNK1 & SETBP1 | 0.298 | 0.001 |
| ETNK1 & SF3B1 | -0.039 | 0.679 |
| ETNK1 & SRSF2 | -0.074 | 0.431 |
| ETNK1 & STAG2 | -0.041 | 0.662 |
| ETNK1 & TET2 | -0.085 | 0.367 |
| ETNK1 & TP53 | -0.041 | 0.662 |
| ETNK1 & U2AF1 | 0.268 | 0.004 |
| ETNK1 & WT1 | -0.018 | 0.850 |
| ETNK1 & ZRSR2 | -0.041 | 0.662 |
| ETNK1 & Normal karyotype | 0.000 | 1.000 |
| ETNK1 & Complex karyotype | -0.060 | 0.532 |
| ETV6 & EZH2 | -0.046 | 0.628 |
| ETV6 & FLT3 | -0.020 | 0.832 |
| ETV6 & GATA2 | -0.029 | 0.762 |
| ETV6 & IDH1 | -0.041 | 0.666 |
| ETV6 & IDH2 | -0.020 | 0.832 |
| ETV6 & JAK2 | -0.029 | 0.762 |
| ETV6 & KRAS | -0.035 | 0.710 |
| ETV6 & MLL3 | -0.029 | 0.762 |
| ETV6 & NF1 | -0.029 | 0.762 |
| ETV6 & NRAS | 0.109 | 0.249 |
| ETV6 & PHF6 | -0.029 | 0.762 |
| ETV6 & PTPN11 | 0.232 | 0.013 |
| ETV6 & RUNX1 | -0.087 | 0.360 |
| ETV6 & SETBP1 | 0.372 | 0.000 |
| ETV6 & SF3B1 | -0.063 | 0.507 |
| ETV6 & SRSF2 | 0.284 | 0.002 |
| ETV6 & STAG2 | -0.066 | 0.483 |
| ETV6 & TET2 | -0.042 | 0.655 |
| ETV6 & TP53 | -0.066 | 0.483 |
| ETV6 & U2AF1 | 0.141 | 0.134 |
| ETV6 & WT1 | -0.029 | 0.762 |
| ETV6 & ZRSR2 | -0.066 | 0.483 |
| ETV6 & Normal karyotype | -0.218 | 0.022 |
| ETV6 & Complex karyotype | -0.097 | 0.316 |
| EZH2 & FLT3 | -0.020 | 0.832 |
| EZH2 & GATA2 | -0.029 | 0.762 |
| EZH2 & IDH1 | -0.041 | 0.666 |
| EZH2 & IDH2 | -0.020 | 0.832 |
| EZH2 & JAK2 | -0.029 | 0.762 |
| EZH2 & KRAS | -0.035 | 0.710 |
| EZH2 & MLL3 | -0.029 | 0.762 |
| EZH2 & NF1 | -0.029 | 0.762 |
| EZH2 & NRAS | 0.109 | 0.249 |
| EZH2 & PHF6 | -0.029 | 0.762 |
| EZH2 & PTPN11 | -0.035 | 0.710 |
| EZH2 & RUNX1 | 0.037 | 0.698 |
| EZH2 & SETBP1 | 0.163 | 0.083 |
| EZH2 & SF3B1 | -0.063 | 0.507 |
| EZH2 & SRSF2 | -0.019 | 0.845 |
| EZH2 & STAG2 | 0.236 | 0.011 |
| EZH2 & TET2 | -0.042 | 0.655 |
| EZH2 & TP53 | 0.085 | 0.369 |
| EZH2 & U2AF1 | 0.141 | 0.134 |
| EZH2 & WT1 | -0.029 | 0.762 |
| EZH2 & ZRSR2 | -0.066 | 0.483 |
| EZH2 & Normal karyotype | 0.218 | 0.022 |
| EZH2 & Complex karyotype | -0.097 | 0.316 |
| FLT3 & GATA2 | -0.013 | 0.894 |
| FLT3 & IDH1 | -0.018 | 0.850 |
| FLT3 & IDH2 | -0.009 | 0.926 |
| FLT3 & JAK2 | -0.013 | 0.894 |
| FLT3 & KRAS | -0.015 | 0.870 |
| FLT3 & MLL3 | -0.013 | 0.894 |
| FLT3 & NF1 | -0.013 | 0.894 |
| FLT3 & NRAS | -0.026 | 0.785 |
| FLT3 & PHF6 | -0.013 | 0.894 |
| FLT3 & PTPN11 | -0.015 | 0.870 |
| FLT3 & RUNX1 | 0.233 | 0.013 |
| FLT3 & SETBP1 | -0.020 | 0.832 |
| FLT3 & SF3B1 | -0.028 | 0.771 |
| FLT3 & SRSF2 | -0.052 | 0.580 |
| FLT3 & STAG2 | -0.029 | 0.758 |
| FLT3 & TET2 | 0.147 | 0.118 |
| FLT3 & TP53 | -0.029 | 0.758 |
| FLT3 & U2AF1 | -0.022 | 0.815 |
| FLT3 & WT1 | -0.013 | 0.894 |
| FLT3 & ZRSR2 | -0.029 | 0.758 |
| FLT3 & Normal karyotype | -0.096 | 0.320 |
| FLT3 & Complex karyotype | -0.042 | 0.660 |
| IDH1 & IDH2 | -0.018 | 0.850 |
| IDH1 & JAK2 | -0.025 | 0.788 |
| IDH1 & KRAS | -0.031 | 0.741 |
| IDH1 & MLL3 | -0.025 | 0.788 |
| IDH1 & NF1 | 0.338 | 0.000 |
| IDH1 & NRAS | -0.052 | 0.580 |
| IDH1 & PHF6 | -0.025 | 0.788 |
| IDH1 & PTPN11 | -0.031 | 0.741 |
| IDH1 & RUNX1 | 0.060 | 0.525 |
| IDH1 & SETBP1 | -0.041 | 0.666 |
| IDH1 & SF3B1 | -0.056 | 0.555 |
| IDH1 & SRSF2 | 0.230 | 0.014 |
| IDH1 & STAG2 | -0.059 | 0.532 |
| IDH1 & TET2 | -0.122 | 0.197 |
| IDH1 & TP53 | -0.059 | 0.532 |
| IDH1 & U2AF1 | -0.045 | 0.635 |
| IDH1 & WT1 | 0.338 | 0.000 |
| IDH1 & ZRSR2 | -0.059 | 0.532 |
| IDH1 & Normal karyotype | 0.097 | 0.313 |
| IDH1 & Complex karyotype | -0.086 | 0.372 |
| IDH2 & JAK2 | -0.013 | 0.894 |
| IDH2 & KRAS | -0.015 | 0.870 |
| IDH2 & MLL3 | -0.013 | 0.894 |
| IDH2 & NF1 | -0.013 | 0.894 |
| IDH2 & NRAS | -0.026 | 0.785 |
| IDH2 & PHF6 | -0.013 | 0.894 |
| IDH2 & PTPN11 | -0.015 | 0.870 |
| IDH2 & RUNX1 | -0.038 | 0.688 |
| IDH2 & SETBP1 | 0.439 | 0.000 |
| IDH2 & SF3B1 | -0.028 | 0.771 |
| IDH2 & SRSF2 | 0.169 | 0.072 |
| IDH2 & STAG2 | -0.029 | 0.758 |
| IDH2 & TET2 | -0.060 | 0.526 |
| IDH2 & TP53 | -0.029 | 0.758 |
| IDH2 & U2AF1 | -0.022 | 0.815 |
| IDH2 & WT1 | -0.013 | 0.894 |
| IDH2 & ZRSR2 | -0.029 | 0.758 |
| IDH2 & Normal karyotype | -0.096 | 0.320 |
| IDH2 & Complex karyotype | -0.042 | 0.660 |
| JAK2 & KRAS | -0.022 | 0.817 |
| JAK2 & MLL3 | -0.018 | 0.850 |
| JAK2 & NF1 | -0.018 | 0.850 |
| JAK2 & NRAS | -0.037 | 0.698 |
| JAK2 & PHF6 | -0.018 | 0.850 |
| JAK2 & PTPN11 | -0.022 | 0.817 |
| JAK2 & RUNX1 | -0.054 | 0.568 |
| JAK2 & SETBP1 | -0.029 | 0.762 |
| JAK2 & SF3B1 | -0.039 | 0.679 |
| JAK2 & SRSF2 | 0.083 | 0.382 |
| JAK2 & STAG2 | -0.041 | 0.662 |
| JAK2 & TET2 | 0.062 | 0.512 |
| JAK2 & TP53 | -0.041 | 0.662 |
| JAK2 & U2AF1 | 0.268 | 0.004 |
| JAK2 & WT1 | -0.018 | 0.850 |
| JAK2 & ZRSR2 | -0.041 | 0.662 |
| JAK2 & Normal karyotype | -0.000 | 1.000 |
| JAK2 & Complex karyotype | 0.124 | 0.198 |
| KRAS & MLL3 | 0.395 | 0.000 |
| KRAS & NF1 | -0.022 | 0.817 |
| KRAS & NRAS | -0.045 | 0.633 |
| KRAS & PHF6 | -0.022 | 0.817 |
| KRAS & PTPN11 | -0.027 | 0.775 |
| KRAS & RUNX1 | -0.066 | 0.483 |
| KRAS & SETBP1 | -0.035 | 0.710 |
| KRAS & SF3B1 | -0.048 | 0.611 |
| KRAS & SRSF2 | 0.037 | 0.693 |
| KRAS & STAG2 | -0.051 | 0.590 |
| KRAS & TET2 | 0.137 | 0.147 |
| KRAS & TP53 | -0.051 | 0.590 |
| KRAS & U2AF1 | -0.039 | 0.682 |
| KRAS & WT1 | -0.022 | 0.817 |
| KRAS & ZRSR2 | -0.051 | 0.590 |
| KRAS & Normal karyotype | 0.056 | 0.562 |
| KRAS & Complex karyotype | 0.077 | 0.425 |
| MLL3 & NF1 | -0.018 | 0.850 |
| MLL3 & NRAS | -0.037 | 0.698 |
| MLL3 & PHF6 | -0.018 | 0.850 |
| MLL3 & PTPN11 | -0.022 | 0.817 |
| MLL3 & RUNX1 | -0.054 | 0.568 |
| MLL3 & SETBP1 | -0.029 | 0.762 |
| MLL3 & SF3B1 | -0.039 | 0.679 |
| MLL3 & SRSF2 | 0.083 | 0.382 |
| MLL3 & STAG2 | -0.041 | 0.662 |
| MLL3 & TET2 | 0.209 | 0.025 |
| MLL3 & TP53 | -0.041 | 0.662 |
| MLL3 & U2AF1 | -0.031 | 0.739 |
| MLL3 & WT1 | -0.018 | 0.850 |
| MLL3 & ZRSR2 | -0.041 | 0.662 |
| MLL3 & Normal karyotype | 0.096 | 0.320 |
| MLL3 & Complex karyotype | -0.042 | 0.660 |
| NF1 & NRAS | -0.037 | 0.698 |
| NF1 & PHF6 | -0.018 | 0.850 |
| NF1 & PTPN11 | -0.022 | 0.817 |
| NF1 & RUNX1 | -0.054 | 0.568 |
| NF1 & SETBP1 | -0.029 | 0.762 |
| NF1 & SF3B1 | -0.039 | 0.679 |
| NF1 & SRSF2 | 0.083 | 0.382 |
| NF1 & STAG2 | -0.041 | 0.662 |
| NF1 & TET2 | 0.062 | 0.512 |
| NF1 & TP53 | -0.041 | 0.662 |
| NF1 & U2AF1 | -0.031 | 0.739 |
| NF1 & WT1 | -0.018 | 0.850 |
| NF1 & ZRSR2 | 0.195 | 0.038 |
| NF1 & Normal karyotype | 0.136 | 0.156 |
| NF1 & Complex karyotype | -0.060 | 0.532 |
| NRAS & PHF6 | -0.037 | 0.698 |
| NRAS & PTPN11 | -0.045 | 0.633 |
| NRAS & RUNX1 | 0.087 | 0.359 |
| NRAS & SETBP1 | 0.109 | 0.249 |
| NRAS & SF3B1 | 0.047 | 0.620 |
| NRAS & SRSF2 | -0.072 | 0.445 |
| NRAS & STAG2 | 0.279 | 0.003 |
| NRAS & TET2 | -0.100 | 0.292 |
| NRAS & TP53 | -0.085 | 0.367 |
| NRAS & U2AF1 | -0.065 | 0.494 |
| NRAS & WT1 | -0.037 | 0.698 |
| NRAS & ZRSR2 | 0.036 | 0.702 |
| NRAS & Normal karyotype | 0.186 | 0.051 |
| NRAS & Complex karyotype | -0.115 | 0.230 |
| PTPN11 & RUNX1 | 0.091 | 0.334 |
| PTPN11 & SETBP1 | 0.232 | 0.013 |
| PTPN11 & SF3B1 | -0.048 | 0.611 |
| PTPN11 & SRSF2 | 0.166 | 0.077 |
| PTPN11 & STAG2 | 0.143 | 0.130 |
| PTPN11 & TET2 | -0.105 | 0.267 |
| PTPN11 & TP53 | -0.051 | 0.590 |
| PTPN11 & U2AF1 | -0.039 | 0.682 |
| PTPN11 & WT1 | -0.022 | 0.817 |
| PTPN11 & ZRSR2 | -0.051 | 0.590 |
| PTPN11 & Normal karyotype | -0.056 | 0.562 |
| PTPN11 & Complex karyotype | -0.074 | 0.442 |
| RUNX1 & SETBP1 | -0.087 | 0.360 |
| RUNX1 & SF3B1 | -0.118 | 0.210 |
| RUNX1 & SRSF2 | 0.013 | 0.895 |
| RUNX1 & STAG2 | 0.232 | 0.013 |
| RUNX1 & TET2 | 0.076 | 0.420 |
| RUNX1 & TP53 | -0.125 | 0.184 |
| RUNX1 & U2AF1 | -0.095 | 0.313 |
| RUNX1 & WT1 | 0.138 | 0.142 |
| RUNX1 & ZRSR2 | 0.053 | 0.574 |
| RUNX1 & Normal karyotype | -0.000 | 1.000 |
| RUNX1 & Complex karyotype | 0.027 | 0.783 |
| SETBP1 & SF3B1 | -0.063 | 0.507 |
| SETBP1 & SRSF2 | 0.284 | 0.002 |
| SETBP1 & STAG2 | -0.066 | 0.483 |
| SETBP1 & TET2 | -0.137 | 0.147 |
| SETBP1 & TP53 | -0.066 | 0.483 |
| SETBP1 & U2AF1 | 0.141 | 0.134 |
| SETBP1 & WT1 | -0.029 | 0.762 |
| SETBP1 & ZRSR2 | -0.066 | 0.483 |
| SETBP1 & Normal karyotype | -0.131 | 0.173 |
| SETBP1 & Complex karyotype | -0.097 | 0.316 |
| SF3B1 & SRSF2 | -0.163 | 0.083 |
| SF3B1 & STAG2 | -0.091 | 0.337 |
| SF3B1 & TET2 | -0.043 | 0.646 |
| SF3B1 & TP53 | -0.091 | 0.337 |
| SF3B1 & U2AF1 | -0.069 | 0.466 |
| SF3B1 & WT1 | -0.039 | 0.679 |
| SF3B1 & ZRSR2 | -0.091 | 0.337 |
| SF3B1 & Normal karyotype | 0.166 | 0.083 |
| SF3B1 & Complex karyotype | -0.132 | 0.169 |
| SRSF2 & STAG2 | 0.192 | 0.041 |
| SRSF2 & TET2 | 0.145 | 0.124 |
| SRSF2 & TP53 | -0.100 | 0.291 |
| SRSF2 & U2AF1 | -0.131 | 0.164 |
| SRSF2 & WT1 | -0.074 | 0.431 |
| SRSF2 & ZRSR2 | -0.173 | 0.066 |
| SRSF2 & Normal karyotype | 0.148 | 0.123 |
| SRSF2 & Complex karyotype | -0.138 | 0.150 |
| STAG2 & TET2 | 0.007 | 0.939 |
| STAG2 & TP53 | 0.013 | 0.887 |
| STAG2 & U2AF1 | -0.073 | 0.440 |
| STAG2 & WT1 | -0.041 | 0.662 |
| STAG2 & ZRSR2 | 0.013 | 0.887 |
| STAG2 & Normal karyotype | 0.190 | 0.047 |
| STAG2 & Complex karyotype | -0.140 | 0.145 |
| TET2 & TP53 | 0.007 | 0.939 |
| TET2 & U2AF1 | -0.150 | 0.110 |
| TET2 & WT1 | -0.085 | 0.367 |
| TET2 & ZRSR2 | 0.281 | 0.002 |
| TET2 & Normal karyotype | 0.182 | 0.057 |
| TET2 & Complex karyotype | -0.113 | 0.239 |
| TP53 & U2AF1 | -0.073 | 0.440 |
| TP53 & WT1 | -0.041 | 0.662 |
| TP53 & ZRSR2 | -0.096 | 0.309 |
| TP53 & Normal karyotype | -0.253 | 0.008 |
| TP53 & Complex karyotype | 0.544 | 0.000 |
| U2AF1 & WT1 | -0.031 | 0.739 |
| U2AF1 & ZRSR2 | -0.073 | 0.440 |
| U2AF1 & Normal karyotype | 0.000 | 1.000 |
| U2AF1 & Complex karyotype | -0.106 | 0.269 |
| WT1 & ZRSR2 | -0.041 | 0.662 |
| WT1 & Normal karyotype | -0.136 | 0.156 |
| WT1 & Complex karyotype | -0.060 | 0.532 |
| ZRSR2 & Normal karyotype | 0.126 | 0.188 |
| ZRSR2 & Complex karyotype | -0.140 | 0.145 |
| Normal karyotype & Complex karyotype | -0.442 | 0.000 |
