# Supplementary material for: Impact of the number of mutations in survival and response outcomes to hypomethylating agents in patients with myelodysplastic syndromes or myelodysplastic/myeloproliferative neoplasms
Source: Oncotarget. 2018 Jan 3;9(11):9714–27. doi: 10.18632/oncotarget.23882 (PMC5839396; doi:10.18632/oncotarget.23882)
Supplement: Supplementary file 4 [file oncotarget-09-9714-s004.docx]

**Supplementary Table 3: Univariate analysis for survival of identified mutations**

| Table S3. Univariate Analysis for Overall Survival | | | | | | | |
| --- | --- | --- | --- | --- | --- | --- | --- |
|  | N | Events | Median | log-rank | HR | 95% CI for HR | p-value |
| Age | 114 | 51 | 28.80 |  | 1.02 | (0.99-1.05) | 0.135 |
| WBC | 112 | 50 | 28.80 |  | 1.02 | (1.00-1.03) | 0.089 |
| ANC | 112 | 50 | 28.80 |  | 1.02 | (0.99-1.05) | 0.231 |
| RBC | 112 | 50 | 28.80 |  | 0.60 | (0.41-0.88) | 0.009 |
| PLT | 112 | 50 | 28.80 |  | 1.00 | (1.00-1.00) | 0.077 |
| HGB | 112 | 50 | 28.80 |  | 0.79 | (0.68-0.90) | 0.001 |
| NEUT | 112 | 50 | 28.80 |  | 0.99 | (0.98-1.01) | 0.297 |
| PBBL | 112 | 50 | 28.80 |  | 1.14 | (1.06-1.23) | 0.001 |
| BMBL | 111 | 50 | 28.80 |  | 1.06 | (1.00-1.11) | 0.034 |
| Treatment |  |  |  |  |  |  |  |
| HMA | 65 | 35 | 21.30 | 0.291 |  |  |  |
| AraC | 10 | 6 | 26.90 |  | 0.98 | (0.41-2.32) | 0.957 |
| Other | 6 | 2 | NR |  | 0.33 | (0.08-1.41) | 0.135 |
| Transformation |  |  |  |  |  |  |  |
| No | 97 | 38 | 46.50 | 0.007 |  |  |  |
| Yes | 15 | 12 | 15.43 |  | 2.40 | (1.25-4.62) | 0.009 |
| Therapy Related |  |  |  |  |  |  |  |
| No | 94 | 41 | 41.23 | 0.539 |  |  |  |
| Yes | 19 | 10 | 26.90 |  | 1.24 | (0.62-2.48) | 0.540 |
| IPSS |  |  |  |  |  |  |  |
| Low | 22 | 5 | 46.50 | 0.003 |  |  |  |
| INT-1 | 45 | 18 | 41.23 |  | 1.88 | (0.70-5.06) | 0.213 |
| INT-2 | 29 | 18 | 15.03 |  | 4.55 | (1.68-12.34) | 0.003 |
| High | 9 | 5 | 14.60 |  | 3.48 | (1.00-12.08) | 0.050 |
| IPSS |  |  |  |  |  |  |  |
| Low/INT-1 | 67 | 23 | 43.20 | 0.001 |  |  |  |
| INT-2/High | 38 | 23 | 15.03 |  | 2.70 | (1.50-4.84) | 0.001 |
| IPSSR |  |  |  |  |  |  |  |
| VL | 11 | 2 | NR | <0.001 |  |  |  |
| L | 23 | 6 | NR |  | 1.19 | (0.24-5.91) | 0.833 |
| I | 33 | 12 | 41.23 |  | 2.05 | (0.46-9.18) | 0.347 |
| H | 28 | 19 | 14.60 |  | 5.99 | (1.39-25.77) | 0.016 |
| VH | 13 | 9 | 13.83 |  | 6.13 | (1.32-28.51) | 0.021 |
| IPSSR |  |  |  |  |  |  |  |
| VL/L/I | 67 | 20 | 46.50 | <0.001 |  |  |  |
| H/VH | 41 | 28 | 14.03 |  | 3.88 | (2.17-6.94) | <0.001 |
| ANC<0.80 |  |  |  |  |  |  |  |
| No | 82 | 36 | 41.23 | 0.812 |  |  |  |
| Yes | 30 | 14 | 27.53 |  | 1.08 | (0.58-2.00) | 0.812 |
| PLT<50 |  |  |  |  |  |  |  |
| No | 80 | 26 | 46.50 | <0.001 |  |  |  |
| Yes | 32 | 24 | 13.83 |  | 3.77 | (2.14-6.64) | <0.001 |
| Hgb<8 |  |  |  |  |  |  |  |
| No | 105 | 46 | 41.23 | 0.548 |  |  |  |
| Yes | 7 | 4 | 23.30 |  | 1.37 | (0.49-3.80) | 0.550 |
| BMBL>10 |  |  |  |  |  |  |  |
| No | 93 | 40 | 41.23 | 0.121 |  |  |  |
| Yes | 18 | 10 | 14.60 |  | 1.72 | (0.86-3.45) | 0.126 |
| IPSSR cytogenetics |  |  |  |  |  |  |  |
| Very good risk | 3 | 2 | 27.97 | 0.001 |  |  |  |
| Good risk | 62 | 19 | 46.50 |  | 0.64 | (0.15-2.78) | 0.554 |
| Intermediate risk | 24 | 14 | 21.30 |  | 1.37 | (0.31-6.07) | 0.682 |
| High risk | 8 | 4 | 28.73 |  | 1.06 | (0.19-5.79) | 0.949 |
| Very high risk | 13 | 10 | 13.10 |  | 3.22 | (0.69-15.06) | 0.138 |
| Diploid cytogenetics |  |  |  |  |  |  |  |
| No | 55 | 33 | 21.30 | 0.002 |  |  |  |
| Yes | 55 | 16 | 46.50 |  | 0.40 | (0.22-0.73) | 0.003 |
| High risk cytogenetics |  |  |  |  |  |  |  |
| No | 85 | 32 | 43.20 | 0.001 |  |  |  |
| Yes | 25 | 17 | 14.63 |  | 2.71 | (1.50-4.92) | 0.001 |
| Complex cytogenetics |  |  |  |  |  |  |  |
| No | 92 | 35 | 43.20 | <0.001 |  |  |  |
| Yes | 18 | 14 | 13.83 |  | 3.22 | (1.70-6.09) | <0.001 |
| Monosomal karyotype |  |  |  |  |  |  |  |
| No | 95 | 37 | 43.20 | <0.001 |  |  |  |
| Yes | 15 | 12 | 13.10 |  | 3.87 | (1.97-7.58) | <0.001 |
| -Y |  |  |  |  |  |  |  |
| Negative | 105 | 45 | 41.23 | 0.167 |  |  |  |
| Positive | 5 | 4 | 15.43 |  | 2.03 | (0.73-5.68) | 0.176 |
| Chromosome 3 abnormality |  |  |  |  |  |  |  |
| Negative | 104 | 47 | 41.23 | 0.416 |  |  |  |
| Positive | 6 | 2 | . |  | 0.56 | (0.14-2.31) | 0.423 |
| Del(5q) |  |  |  |  |  |  |  |
| Negative | 99 | 40 | 43.20 | <0.001 |  |  |  |
| Positive | 11 | 9 | 11.80 |  | 3.81 | (1.81-8.01) | <0.001 |
| Del(7q) |  |  |  |  |  |  |  |
| Negative | 97 | 37 | 43.20 | <0.001 |  |  |  |
| Positive | 13 | 12 | 11.80 |  | 5.59 | (2.84-11.01) | <0.001 |
| Trisomy 8 |  |  |  |  |  |  |  |
| Negative | 99 | 42 | 41.23 | 0.172 |  |  |  |
| Positive | 11 | 7 | 14.03 |  | 1.74 | (0.78-3.87) | 0.178 |
| Del12 |  |  |  |  |  |  |  |
| Negative | 104 | 44 | 41.23 | 0.015 |  |  |  |
| Positive | 6 | 5 | 13.83 |  | 3.06 | (1.19-7.88) | 0.021 |
| Del17 |  |  |  |  |  |  |  |
| Negative | 104 | 45 | 41.23 | 0.182 |  |  |  |
| Positive | 6 | 4 | 13.10 |  | 1.99 | (0.71-5.56) | 0.191 |
| Del20q |  |  |  |  |  |  |  |
| Negative | 100 | 42 | 41.23 | 0.007 |  |  |  |
| Positive | 10 | 7 | 11.87 |  | 2.92 | (1.30-6.58) | 0.010 |
| ASXL1 |  |  |  |  |  |  |  |
| Negative | 91 | 44 | 27.97 | 0.114 |  |  |  |
| Positive | 23 | 7 | . |  | 0.53 | (0.24-1.18) | 0.120 |
| BCOR |  |  |  |  |  |  |  |
| Negative | 108 | 46 | 41.23 | 0.022 |  |  |  |
| Positive | 6 | 5 | 10.83 |  | 2.85 | (1.12-7.29) | 0.029 |
| CUX1 |  |  |  |  |  |  |  |
| Negative | 109 | 50 | 28.80 | 0.392 |  |  |  |
| Positive | 5 | 1 | . |  | 0.43 | (0.06-3.13) | 0.406 |
| DNMT3A |  |  |  |  |  |  |  |
| Negative | 107 | 47 | 41.23 | 0.209 |  |  |  |
| Positive | 7 | 4 | 14.67 |  | 1.91 | (0.68-5.34) | 0.217 |
| ETV6 |  |  |  |  |  |  |  |
| Negative | 109 | 48 | 41.23 | 0.525 |  |  |  |
| Positive | 5 | 3 | 21.30 |  | 1.46 | (0.45-4.70) | 0.527 |
| EZH2 |  |  |  |  |  |  |  |
| Negative | 109 | 48 | 41.23 | 0.210 |  |  |  |
| Positive | 5 | 3 | 7.40 |  | 2.08 | (0.64-6.73) | 0.220 |
| NRAS |  |  |  |  |  |  |  |
| Negative | 106 | 45 | 41.23 | 0.208 |  |  |  |
| Positive | 8 | 6 | 26.90 |  | 1.72 | (0.73-4.04) | 0.214 |
| RUNX1 |  |  |  |  |  |  |  |
| Negative | 98 | 42 | 41.23 | 0.241 |  |  |  |
| Positive | 16 | 9 | 16.67 |  | 1.54 | (0.74-3.18) | 0.245 |
| SETBP1 |  |  |  |  |  |  |  |
| Negative | 109 | 47 | 41.23 | 0.247 |  |  |  |
| Positive | 5 | 4 | 27.97 |  | 1.82 | (0.65-5.07) | 0.254 |
| SF3B1 |  |  |  |  |  |  |  |
| Negative | 105 | 51 | 28.73 | 0.023 |  |  |  |
| Positive | 9 | 0 | . |  | 0.00 | (0.00- .) | 0.999 |
| SRSF2 |  |  |  |  |  |  |  |
| Negative | 87 | 39 | 41.23 | 0.815 |  |  |  |
| Positive | 27 | 12 | 28.80 |  | 1.08 | (0.56-2.07) | 0.815 |
| STAG2 |  |  |  |  |  |  |  |
| Negative | 104 | 45 | 41.23 | 0.034 |  |  |  |
| Positive | 10 | 6 | 10.50 |  | 2.45 | (1.04-5.80) | 0.041 |
| TET2 |  |  |  |  |  |  |  |
| Negative | 81 | 35 | 43.20 | 0.382 |  |  |  |
| Positive | 33 | 16 | 26.90 |  | 1.30 | (0.72-2.36) | 0.384 |
| TP53 |  |  |  |  |  |  |  |
| Negative | 104 | 43 | 41.23 | <0.001 |  |  |  |
| Positive | 10 | 8 | 12.23 |  | 5.25 | (2.37-11.63) | <0.001 |
| U2AF1 |  |  |  |  |  |  |  |
| Negative | 108 | 48 | 41.23 | 0.998 |  |  |  |
| Positive | 6 | 3 | 27.97 |  | 1.00 | (0.31-3.21) | 0.998 |
| ZRSR2 |  |  |  |  |  |  |  |
| Negative | 104 | 47 | 41.23 | 0.963 |  |  |  |
| Positive | 10 | 4 | 26.90 |  | 0.98 | (0.35-2.72) | 0.963 |
| Mutations≥3 |  |  |  |  |  |  |  |
| No | 76 | 29 | 43.20 | 0.020 |  |  |  |
| Yes | 38 | 22 | 21.30 |  | 1.94 | (1.10-3.41) | 0.022 |
