# Supplementary material for: Impact of the number of mutations in survival and response outcomes to hypomethylating agents in patients with myelodysplastic syndromes or myelodysplastic/myeloproliferative neoplasms
Source: Oncotarget. 2018 Jan 3;9(11):9714–27. doi: 10.18632/oncotarget.23882 (PMC5839396; doi:10.18632/oncotarget.23882)
Supplement: Supplementary file 5 [file oncotarget-09-9714-s005.docx]

**Supplementary Table 4: Univariate analysis for survival of in patients with MDS**

| Table S4. Univariate Analysis for OS (MDS) | | | | | | | |
| --- | --- | --- | --- | --- | --- | --- | --- |
|  | N | Events | Median | log-rank | HR | 95% CI for HR | p-value |
| Age | 83 | 35 | 28.80 |  | 1.03 | (1.00-1.06) | 0.056 |
| WBC | 81 | 34 | 28.80 |  | 0.94 | (0.81-1.11) | 0.478 |
| ANC | 81 | 34 | 28.80 |  | 0.88 | (0.71-1.10) | 0.267 |
| RBC | 81 | 34 | 28.80 |  | 0.55 | (0.34-0.90) | 0.018 |
| PLT | 81 | 34 | 28.80 |  | 1.00 | (1.00-1.00) | 0.356 |
| HGB | 81 | 34 | 28.80 |  | 0.78 | (0.65-0.92) | 0.003 |
| NEUT | 81 | 34 | 28.80 |  | 0.99 | (0.97-1.01) | 0.176 |
| PBBL | 81 | 34 | 28.80 |  | 1.14 | (1.04-1.26) | 0.006 |
| BMBL | 81 | 34 | 28.80 |  | 1.08 | (1.02-1.15) | 0.011 |
| Treatment |  |  |  |  |  |  |  |
| HMA | 44 | 24 | 21.30 | 0.266 |  |  |  |
| AraC | 7 | 3 | 26.90 |  | 0.73 | (0.22-2.45) | 0.616 |
| Other | 6 | 2 | NR |  | 0.32 | (0.07-1.40) | 0.131 |
| Transformation |  |  |  |  |  |  |  |
| No | 73 | 27 | 41.23 | 0.015 |  |  |  |
| Yes | 10 | 8 | 14.60 |  | 2.61 | (1.17-5.80) | 0.019 |
| Therapy Related |  |  |  |  |  |  |  |
| No | 66 | 26 | 41.23 | 0.438 |  |  |  |
| Yes | 16 | 9 | 26.90 |  | 1.35 | (0.63-2.89) | 0.439 |
| IPSShl |  |  |  |  |  |  |  |
| Low/INT-1 | 53 | 18 | 41.23 | 0.004 |  |  |  |
| INT-2/High | 30 | 17 | 15.43 |  | 2.59 | (1.32-5.08) | 0.006 |
| MIPSS-R grouped |  |  |  |  |  |  |  |
| 0-0.5 | 34 | 7 | NR | <0.001 |  |  |  |
| 1-2 | 36 | 17 | 28.73 |  | 2.64 | (1.09-6.39) | 0.032 |
| 2.5-3.5 | 13 | 11 | 13.10 |  | 13.83 | (4.94-38.73) | <0.001 |
| IPSSb |  |  |  |  |  |  |  |
| 0 | 51 | 17 | NR | 0.008 |  |  |  |
| 1 | 29 | 16 | 15.43 |  | 2.47 | (1.24-4.94) | 0.011 |
| IPSSRHIGH |  |  |  |  |  |  |  |
| VL/L/I | 50 | 13 | NR | <0.001 |  |  |  |
| V/VH | 33 | 22 | 14.63 |  | 4.37 | (2.18-8.77) | <0.001 |
| ANC<0.80 |  |  |  |  |  |  |  |
| No | 53 | 21 | 41.23 | 0.610 |  |  |  |
| Yes | 28 | 13 | 27.53 |  | 1.20 | (0.60-2.40) | 0.610 |
| PLT<50 |  |  |  |  |  |  |  |
| No | 58 | 17 | NR | <0.001 |  |  |  |
| Yes | 23 | 17 | 13.10 |  | 4.19 | (2.12-8.29) | <0.001 |
| Hgb<8 |  |  |  |  |  |  |  |
| No | 75 | 31 | 41.23 | 0.850 |  |  |  |
| Yes | 6 | 3 | 27.27 |  | 1.12 | (0.34-3.68) | 0.850 |
|  |  |  |  |  |  |  |  |
| BMBL>10 |  |  |  |  |  |  |  |
| No | 67 | 26 | 41.23 | 0.045 |  |  |  |
| Yes | 14 | 8 | 14.60 |  | 2.23 | (1.00-5.00) | 0.050 |
| CG_IPSSR |  |  |  |  |  |  |  |
| Very good risk | 3 | 2 | 27.97 | 0.007 |  |  |  |
| Good risk | 42 | 11 | NR |  | 0.55 | (0.12-2.49) | 0.435 |
| Intermediate risk | 15 | 8 | 25.37 |  | 1.05 | (0.22-4.94) | 0.955 |
| High risk | 7 | 3 | 28.73 |  | 0.88 | (0.15-5.29) | 0.890 |
| Very high risk | 12 | 9 | 13.10 |  | 2.74 | (0.58-13.00) | 0.205 |
| Normal Karyotype |  |  |  |  |  |  |  |
| No | 43 | 24 | 25.37 | 0.022 |  |  |  |
| Yes | 36 | 9 | NR |  | 0.42 | (0.19-0.90) | 0.026 |
| CGHIGHb |  |  |  |  |  |  |  |
| No | 57 | 19 | NR | 0.002 |  |  |  |
| Yes | 22 | 14 | 14.63 |  | 2.87 | (1.43-5.77) | 0.003 |
| Complex karyotype |  |  |  |  |  |  |  |
| No | 63 | 21 | NR | <0.001 |  |  |  |
| Yes | 16 | 12 | 13.83 |  | 3.52 | (1.70-7.31) | 0.001 |
| CGMKb |  |  |  |  |  |  |  |
| No | 65 | 22 | NR | <0.001 |  |  |  |
| Yes | 14 | 11 | 13.10 |  | 4.31 | (2.02-9.18) | <0.001 |
| Chr-Y |  |  |  |  |  |  |  |
| Negative | 75 | 30 | 41.23 | 0.354 |  |  |  |
| Positive | 4 | 3 | 15.43 |  | 1.74 | (0.53-5.75) | 0.360 |
| Chr3 |  |  |  |  |  |  |  |
| Negative | 73 | 31 | 41.23 | 0.472 |  |  |  |
| Positive | 6 | 2 | NR |  | 0.60 | (0.14-2.49) | 0.477 |
| Del(5q) |  |  |  |  |  |  |  |
| Negative | 69 | 25 | 41.23 | <0.001 |  |  |  |
| Positive | 10 | 8 | 11.80 |  | 4.06 | (1.77-9.31) | 0.001 |
| Del(7q) |  |  |  |  |  |  |  |
| Negative | 67 | 22 | NR | <0.001 |  |  |  |
| Positive | 12 | 11 | 11.80 |  | 6.82 | (3.20-14.54) | <0.001 |
| Trisomy 8 |  |  |  |  |  |  |  |
| Negative | 72 | 28 | 41.23 | 0.075 |  |  |  |
| Positive | 7 | 5 | 10.83 |  | 2.33 | (0.89-6.08) | 0.084 |
| Del12b |  |  |  |  |  |  |  |
| Negative | 73 | 28 | 41.23 | 0.010 |  |  |  |
| Positive | 6 | 5 | 13.83 |  | 3.38 | (1.26-9.05) | 0.016 |
| Del(17p) |  |  |  |  |  |  |  |
| Negative | 75 | 30 | 41.23 | 0.135 |  |  |  |
| Positive | 4 | 3 | 13.10 |  | 2.41 | (0.73-7.96) | 0.148 |
| Del(20q) |  |  |  |  |  |  |  |
| Negative | 71 | 28 | 41.23 | 0.110 |  |  |  |
| Positive | 8 | 5 | 13.10 |  | 2.15 | (0.82-5.63) | 0.119 |
| ASXL1 |  |  |  |  |  |  |  |
| Negative | 70 | 33 | 27.97 | 0.034 |  |  |  |
| Positive | 13 | 2 | NR |  | 0.24 | (0.06-1.01) | 0.051 |
| BCOR |  |  |  |  |  |  |  |
| Negative | 79 | 32 | 41.23 | 0.110 |  |  |  |
| Positive | 4 | 3 | 10.83 |  | 2.57 | (0.77-8.53) | 0.124 |
| CUX1 |  |  |  |  |  |  |  |
| Negative | 80 | 35 | 28.73 | 0.158 |  |  |  |
| Positive | 3 | 0 | NR |  | - | - | - |
| DNMT3A |  |  |  |  |  |  |  |
| Negative | 77 | 32 | 41.23 | 0.336 |  |  |  |
| Positive | 6 | 3 | 17.03 |  | 1.78 | (0.54-5.86) | 0.343 |
| ETV6 |  |  |  |  |  |  |  |
| Negative | 81 | 34 | 28.80 | 0.906 |  |  |  |
| Positive | 2 | 1 | 21.30 |  | 1.13 | (0.15-8.31) | 0.906 |
| EZH2 |  |  |  |  |  |  |  |
| Negative | 79 | 33 | 41.23 | 0.642 |  |  |  |
| Positive | 4 | 2 | 28.80 |  | 1.40 | (0.33-5.90) | 0.643 |
| NRAS |  |  |  |  |  |  |  |
| Negative | 77 | 30 | 41.23 | 0.057 |  |  |  |
| Positive | 6 | 5 | 26.90 |  | 2.46 | (0.94-6.42) | 0.066 |
| RUNX1 |  |  |  |  |  |  |  |
| Negative | 69 | 28 | 28.80 | 0.488 |  |  |  |
| Positive | 14 | 7 | 25.37 |  | 1.34 | (0.58-3.07) | 0.490 |
| SETPB1 |  |  |  |  |  |  |  |
| Negative | 81 | 33 | 41.23 | 0.539 |  |  |  |
| Positive | 2 | 2 | 27.97 |  | 1.56 | (0.37-6.55) | 0.542 |
| SF3B1b |  |  |  |  |  |  |  |
| Negative | 74 | 35 | 27.97 | 0.027 |  |  |  |
| Positive | 9 | 0 | NR |  | - | - | - |
| SRSF2 |  |  |  |  |  |  |  |
| Negative | 69 | 30 | 28.73 | 0.673 |  |  |  |
| Positive | 14 | 5 | NR |  | 0.82 | (0.32-2.10) | 0.674 |
| STAG2 |  |  |  |  |  |  |  |
| Negative | 73 | 29 | 41.23 | 0.023 |  |  |  |
| Positive | 10 | 6 | 10.50 |  | 2.70 | (1.11-6.57) | 0.029 |
| TET2 |  |  |  |  |  |  |  |
| Negative | 66 | 28 | 28.80 | 0.852 |  |  |  |
| Positive | 17 | 7 | 41.23 |  | 1.08 | (0.47-2.49) | 0.852 |
| TP53 |  |  |  |  |  |  |  |
| Negative | 75 | 29 | 41.23 | <0.001 |  |  |  |
| Positive | 8 | 6 | 13.10 |  | 5.19 | (2.05-13.12) | 0.001 |
| U2AF1 |  |  |  |  |  |  |  |
| Negative | 80 | 34 | 28.80 | 0.558 |  |  |  |
| Positive | 3 | 1 | 27.97 |  | 0.56 | (0.08-4.07) | 0.563 |
| ZRSR2 |  |  |  |  |  |  |  |
| Negative | 76 | 31 | 41.23 | 0.426 |  |  |  |
| Positive | 7 | 4 | 26.90 |  | 1.53 | (0.53-4.35) | 0.430 |
| Splicing pathway gene mutated |  |  |  |  |  |  |  |
| Negative | 50 | 25 | 27.53 | 0.109 |  |  |  |
| Positive | 33 | 10 | NR |  | 0.55 | (0.27-1.15) | 0.114 |
| Methylation pathway gene mutated |  |  |  |  |  |  |  |
| Negative | 59 | 26 | 28.73 | 0.740 |  |  |  |
| Positive | 24 | 9 | 41.23 |  | 0.88 | (0.41-1.88) | 0.740 |
| Cohesin pathway gene mutated |  |  |  |  |  |  |  |
| Negative | 73 | 29 | 41.23 | 0.023 |  |  |  |
| Positive | 10 | 6 | 10.50 |  | 2.70 | (1.11-6.57) | 0.029 |
| Mutations>=4 |  |  |  |  |  |  |  |
| No | 71 | 30 | 41.23 | 0.866 |  |  |  |
| Yes | 12 | 5 | 28.80 |  | 1.08 | (0.42-2.81) | 0.866 |
| Mutations>=3 |  |  |  |  |  |  |  |
| No | 60 | 23 | 41.23 | 0.286 |  |  |  |
| Yes | 23 | 12 | 27.97 |  | 1.46 | (0.73-2.94) | 0.289 |
