# Supplementary material for: Impact of the number of mutations in survival and response outcomes to hypomethylating agents in patients with myelodysplastic syndromes or myelodysplastic/myeloproliferative neoplasms
Source: Oncotarget. 2018 Jan 3;9(11):9714–27. doi: 10.18632/oncotarget.23882 (PMC5839396; doi:10.18632/oncotarget.23882)
Supplement: Supplementary file 6 [file oncotarget-09-9714-s006.docx]

**Supplementary Table 5: Univariate analysis for survival of in patients with MDS/MPN**

| Table S5. Univariate Analysis for OS (CMML) | | | | | | | |
| --- | --- | --- | --- | --- | --- | --- | --- |
|  | N | Events | Median | log-rank | HR | 95% CI for HR | p-value |
| Age | 31 | 16 | 43.20 |  | 0.94 | (0.87-1.03) | 0.187 |
| WBC | 31 | 16 | 43.20 |  | 1.02 | (1.00-1.04) | 0.110 |
| ANC | 31 | 16 | 43.20 |  | 1.02 | (0.99-1.06) | 0.220 |
| RBC | 31 | 16 | 43.20 |  | 0.60 | (0.30-1.18) | 0.137 |
| PLT | 31 | 16 | 43.20 |  | 0.99 | (0.99-1.00) | 0.088 |
| HGB | 31 | 16 | 43.20 |  | 0.77 | (0.59-1.02) | 0.065 |
| NEUT | 31 | 16 | 43.20 |  | 1.00 | (0.98-1.02) | 0.917 |
| PBBL | 31 | 16 | 43.20 |  | 1.12 | (0.98-1.29) | 0.086 |
| BMBL | 30 | 16 | 43.20 |  | 0.99 | (0.89-1.11) | 0.915 |
| Treatment |  |  |  |  |  |  |  |
| HMA | 21 | 11 | 19.60 | 0.410 |  |  |  |
| AraC | 3 | 3 | 14.67 |  | 1.72 | (0.47-6.34) | 0.415 |
| Transformation |  |  |  |  |  |  |  |
| No | 26 | 12 | 46.50 | 0.245 |  |  |  |
| Yes | 5 | 4 | 18.43 |  | 1.95 | (0.62-6.14) | 0.254 |
| Therapy Related |  |  |  |  |  |  |  |
| No | 28 | 15 | 43.20 | 0.934 |  |  |  |
| Yes | 3 | 1 | 12.23 |  | 1.09 | (0.14-8.35) | 0.934 |
| IPSShl |  |  |  |  |  |  |  |
| Low/INT-1 | 21 | 8 | 46.50 | 0.008 |  |  |  |
| INT-2/High | 10 | 8 | 12.23 |  | 3.57 | (1.31-9.72) | 0.013 |
| MIPSS-R grouped |  |  |  |  |  |  |  |
| 0-0.5 | 14 | 5 | 46.50 | 0.001 |  |  |  |
| 1-2 | 8 | 3 | NR |  | 1.28 | (0.30-5.41) | 0.741 |
| 2.5-3.5 | 9 | 8 | 12.23 |  | 6.65 | (1.94-22.84) | 0.003 |
| IPSSb |  |  |  |  |  |  |  |
| 0 | 16 | 6 | 43.20 | 0.018 |  |  |  |
| 1 | 9 | 7 | 12.23 |  | 3.55 | (1.16-10.86) | 0.026 |
| IPSSRHIGH |  |  |  |  |  |  |  |
| VL/L/I | 21 | 8 | 46.50 | 0.012 |  |  |  |
| V/VH | 10 | 8 | 12.23 |  | 3.31 | (1.23-8.90) | 0.018 |
| ANC<0.80 |  |  |  |  |  |  |  |
| No | 29 | 15 | 43.20 | 0.787 |  |  |  |
| Yes | 2 | 1 | 10.10 |  | 0.75 | (0.10-5.92) | 0.787 |
| PLT<50 |  |  |  |  |  |  |  |
| No | 22 | 9 | 46.50 | 0.021 |  |  |  |
| Yes | 9 | 7 | 14.67 |  | 3.28 | (1.13-9.52) | 0.029 |
| Hgb<8 |  |  |  |  |  |  |  |
| No | 30 | 15 | 43.20 | <0.001 |  |  |  |
| Yes | 1 | 1 | NR |  | 28.50 | (1.78- 455.65) | 0.018 |
|  |  |  |  |  |  |  |  |
|  |  |  |  |  |  |  |  |
| BMBL>10 |  |  |  |  |  |  |  |
| No | 26 | 14 | 43.20 | 0.921 |  |  |  |
| Yes | 4 | 2 | 12.23 |  | 0.93 | (0.21-4.12) | 0.921 |
| Normal Karyotype |  |  |  |  |  |  |  |
| No | 12 | 9 | 13.53 | 0.008 |  |  |  |
| Yes | 19 | 7 | 46.50 |  | 0.25 | (0.08-0.76) | 0.014 |
| CGHIGHb |  |  |  |  |  |  |  |
| No | 28 | 13 | 43.20 | 0.073 |  |  |  |
| Yes | 3 | 3 | 14.67 |  | 3.05 | (0.84-11.01) | 0.089 |
| Complex karyotype |  |  |  |  |  |  |  |
| No | 29 | 14 | 43.20 | 0.165 |  |  |  |
| Yes | 2 | 2 | 8.73 |  | 2.77 | (0.62-12.42) | 0.184 |
| CGMKb |  |  |  |  |  |  |  |
| No | 30 | 15 | 43.20 | 0.020 |  |  |  |
| Yes | 1 | 1 | NR |  | 9.16 | (0.95-88.05) | 0.055 |
| Chr-Y |  |  |  |  |  |  |  |
| Negative | 30 | 15 | 43.20 | 0.020 |  |  |  |
| Positive | 1 | 1 | NR |  | 9.16 | (0.95-88.05) | 0.055 |
| Del(5q) |  |  |  |  |  |  |  |
| Negative | 30 | 15 | 43.20 | 0.020 |  |  |  |
| Positive | 1 | 1 | NR |  | 9.16 | (0.95-88.05) | 0.055 |
| Del(7q) |  |  |  |  |  |  |  |
| Negative | 30 | 15 | 43.20 | 0.278 |  |  |  |
| Positive | 1 | 1 | NR |  | 2.99 | (0.37-23.98) | 0.302 |
| Trisomy 8 |  |  |  |  |  |  |  |
| Negative | 27 | 14 | 43.20 | 0.943 |  |  |  |
| Positive | 4 | 2 | 16.67 |  | 1.06 | (0.24-4.72) | 0.943 |
| Del(17p) |  |  |  |  |  |  |  |
| Negative | 29 | 15 | 43.20 | 0.816 |  |  |  |
| Positive | 2 | 1 | 8.73 |  | 1.27 | (0.17-9.76) | 0.816 |
| Del(20q) |  |  |  |  |  |  |  |
| Negative | 29 | 14 | 43.20 | <0.001 |  |  |  |
| Positive | 2 | 2 | 5.73 |  | 44.71 | (3.96-505.51) | 0.002 |
| ASXL1 |  |  |  |  |  |  |  |
| Negative | 21 | 11 | 43.20 | 0.993 |  |  |  |
| Positive | 10 | 5 | 19.60 |  | 1.00 | (0.35-2.90) | 0.993 |
| BCOR |  |  |  |  |  |  |  |
| Negative | 29 | 14 | 43.20 | 0.084 |  |  |  |
| Positive | 2 | 2 | 10.10 |  | 3.56 | (0.76-16.59) | 0.106 |
| CUX1 |  |  |  |  |  |  |  |
| Negative | 29 | 15 | 43.20 | 0.350 |  |  |  |
| Positive | 2 | 1 | 6.97 |  | 2.63 | (0.32-21.54) | 0.368 |
| DNMT3A |  |  |  |  |  |  |  |
| Negative | 30 | 15 | 43.20 | 0.278 |  |  |  |
| Positive | 1 | 1 | NR |  | 2.99 | (0.37-23.98) | 0.302 |
| ETV6 |  |  |  |  |  |  |  |
| Negative | 28 | 14 | 43.20 | 0.406 |  |  |  |
| Positive | 3 | 2 | 13.53 |  | 1.87 | (0.42-8.45) | 0.413 |
| EZH2 |  |  |  |  |  |  |  |
| Negative | 30 | 15 | 43.20 | <0.001 |  |  |  |
| Positive | 1 | 1 | NR |  | 28.50 | (1.78- 455.65) | 0.018 |
| NRAS |  |  |  |  |  |  |  |
| Negative | 29 | 15 | 43.20 | 0.708 |  |  |  |
| Positive | 2 | 1 | 13.53 |  | 0.68 | (0.09-5.27) | 0.709 |
| RUNX1 |  |  |  |  |  |  |  |
| Negative | 29 | 14 | 43.20 | 0.147 |  |  |  |
| Positive | 2 | 2 | 14.67 |  | 2.99 | (0.63-14.13) | 0.167 |
| SETPB1 |  |  |  |  |  |  |  |
| Negative | 28 | 14 | 43.20 | 0.300 |  |  |  |
| Positive | 3 | 2 | 13.53 |  | 2.17 | (0.48-9.74) | 0.312 |
| SRSF2 |  |  |  |  |  |  |  |
| Negative | 18 | 9 | 46.50 | 0.542 |  |  |  |
| Positive | 13 | 7 | 21.70 |  | 1.37 | (0.49-3.81) | 0.544 |
| TET2 |  |  |  |  |  |  |  |
| Negative | 15 | 7 | 43.20 | 0.500 |  |  |  |
| Positive | 16 | 9 | 21.70 |  | 1.41 | (0.51-3.89) | 0.501 |
| TP53 |  |  |  |  |  |  |  |
| Negative | 29 | 14 | 43.20 | 0.010 |  |  |  |
| Positive | 2 | 2 | 8.73 |  | 6.60 | (1.26-34.43) | 0.025 |
| U2AF1 |  |  |  |  |  |  |  |
| Negative | 28 | 14 | 43.20 | 0.555 |  |  |  |
| Positive | 3 | 2 | 10.10 |  | 1.58 | (0.34-7.23) | 0.558 |
| ZRSR2 |  |  |  |  |  |  |  |
| Negative | 28 | 16 | 21.70 | 0.227 |  |  |  |
| Positive | 3 | 0 | NR |  | - | - | - |
| Splicing pathway gene mutated |  |  |  |  |  |  |  |
| Negative | 12 | 7 | 19.50 | 0.810 |  |  |  |
| Positive | 19 | 9 | 43.20 |  | 1.13 | (0.41-3.09) | 0.810 |
| Methylation pathway gene mutated |  |  |  |  |  |  |  |
| Negative | 14 | 6 | 43.20 | 0.242 |  |  |  |
| Positive | 17 | 10 | 19.60 |  | 1.84 | (0.65-5.20) | 0.249 |
| Mutations>=4 |  |  |  |  |  |  |  |
| No | 25 | 12 | 43.20 | 0.150 |  |  |  |
| Yes | 6 | 4 | 13.53 |  | 2.30 | (0.72-7.36) | 0.162 |
| Mutations>=3 |  |  |  |  |  |  |  |
| No | 16 | 6 | 46.50 | 0.025 |  |  |  |
| Yes | 15 | 10 | 19.50 |  | 3.50 | (1.09-11.19) | 0.035 |
