# Supplementary material for: Impact of the number of mutations in survival and response outcomes to hypomethylating agents in patients with myelodysplastic syndromes or myelodysplastic/myeloproliferative neoplasms
Source: Oncotarget. 2018 Jan 3;9(11):9714–27. doi: 10.18632/oncotarget.23882 (PMC5839396; doi:10.18632/oncotarget.23882)
Supplement: Supplementary file 7 [file oncotarget-09-9714-s007.docx]

**Supplementary Table 6: Univariate analysis for leukemia-free survival in patients with MDS**

| Table S6. Univariate Analysis for LFS (MDS) | | | | | | | |
| --- | --- | --- | --- | --- | --- | --- | --- |
|  | N | Events | Median | log-rank | HR | 95% CI for HR | p-value |
| Age | 83 | 10 | NR |  | 0.98 | (0.94-1.02) | 0.296 |
| WBC | 81 | 10 | NR |  | 0.87 | (0.62-1.21) | 0.403 |
| ANC | 81 | 10 | NR |  | 0.74 | (0.45-1.21) | 0.233 |
| RBC | 81 | 10 | NR |  | 0.48 | (0.19-1.21) | 0.121 |
| PLT | 81 | 10 | NR |  | 1.00 | (0.99-1.00) | 0.568 |
| HGB | 81 | 10 | NR |  | 0.71 | (0.51-0.98) | 0.039 |
| NEUT | 81 | 10 | NR |  | 0.98 | (0.95-1.01) | 0.135 |
| PBBL | 81 | 10 | NR |  | 1.31 | (1.12-1.53) | 0.001 |
| BMBL | 81 | 10 | NR |  | 1.15 | (1.04-1.27) | 0.005 |
| Treatment |  |  |  |  |  |  |  |
| HMA | 44 | 8 | NR | 0.357 |  |  |  |
| AraC | 7 | 2 | NR |  | 1.46 | (0.31-6.95) | 0.631 |
| Other | 6 | 0 | NR |  | - | - | - |
| Therapy Related |  |  |  |  |  |  |  |
| No | 66 | 8 | NR | 0.998 |  |  |  |
| Yes | 16 | 2 | NR |  | 1.00 | (0.21-4.71) | 0.998 |
| IPSShl |  |  |  |  |  |  |  |
| Low/INT-1 | 53 | 3 | NR | 0.003 |  |  |  |
| INT-2/High | 30 | 7 | NR |  | 6.20 | (1.57-24.55) | 0.009 |
| MIPSS-R grouped |  |  |  |  |  |  |  |
| 0-0.5 | 34 | 1 | NR | 0.001 |  |  |  |
| 1-2 | 36 | 5 | NR |  | 5.19 | (0.61-44.44) | 0.133 |
| 2.5-3.5 | 13 | 4 | NR |  | 27.61 | (2.78-273.88) | 0.005 |
| IPSSb |  |  |  |  |  |  |  |
| 0 | 51 | 3 | NR | 0.004 |  |  |  |
| 1 | 29 | 7 | NR |  | 5.95 | (1.51-23.49) | 0.011 |
| IPSSRHIGH |  |  |  |  |  |  |  |
| VL/L/I | 50 | 4 | NR | 0.046 |  |  |  |
| V/VH | 33 | 6 | NR |  | 3.43 | (0.95-12.41) | 0.060 |
| ANC<0.80 |  |  |  |  |  |  |  |
| No | 53 | 5 | NR | 0.327 |  |  |  |
| Yes | 28 | 5 | NR |  | 1.84 | (0.53-6.37) | 0.335 |
| PLT<50 |  |  |  |  |  |  |  |
| No | 58 | 5 | NR | 0.029 |  |  |  |
| Yes | 23 | 5 | NR |  | 3.72 | (1.06-13.07) | 0.041 |
| Hgb<8 |  |  |  |  |  |  |  |
| No | 75 | 10 | NR | 0.351 |  |  |  |
| Yes | 6 | 0 | NR |  | - | - | - |
| BMBL>10 |  |  |  |  |  |  |  |
| No | 67 | 5 | NR | <0.001 |  |  |  |
| Yes | 14 | 5 | NR |  | 7.81 | (2.19-27.76) | 0.002 |
|  |  |  |  |  |  |  |  |
| CG_IPSSR |  |  |  |  |  |  |  |
| Very good risk | 3 | 2 | 26.15 | 0.004 |  |  |  |
| Good risk | 42 | 3 | NR |  | 0.13 | (0.02-0.76) | 0.024 |
| Intermediate risk | 15 | 1 | NR |  | 0.12 | (0.01-1.31) | 0.081 |
| High risk | 7 | 0 | NR |  | - | - | - |
| Very high risk | 12 | 4 | NR |  | 0.91 | (0.16-5.23) | 0.916 |
| Normal Karyotype |  |  |  |  |  |  |  |
| No | 43 | 7 | NR | 0.287 |  |  |  |
| Yes | 36 | 3 | NR |  | 0.49 | (0.13-1.88) | 0.297 |
| CGHIGHb |  |  |  |  |  |  |  |
| No | 57 | 6 | NR | 0.171 |  |  |  |
| Yes | 22 | 4 | NR |  | 2.39 | (0.66-8.59) | 0.184 |
| Complex karyotype |  |  |  |  |  |  |  |
| No | 63 | 6 | NR | 0.039 |  |  |  |
| Yes | 16 | 4 | NR |  | 3.57 | (0.99-12.95) | 0.052 |
| CGMKb |  |  |  |  |  |  |  |
| No | 65 | 7 | NR | 0.094 |  |  |  |
| Yes | 14 | 3 | NR |  | 3.08 | (0.77-12.32) | 0.111 |
| Chr-Y |  |  |  |  |  |  |  |
| Negative | 75 | 8 | NR | 0.027 |  |  |  |
| Positive | 4 | 2 | 26.15 |  | 4.88 | (1.03-23.08) | 0.045 |
| Chr3 |  |  |  |  |  |  |  |
| Negative | 73 | 9 | NR | 0.901 |  |  |  |
| Positive | 6 | 1 | NR |  | 1.14 | (0.14-9.01) | 0.901 |
| Del(5q) |  |  |  |  |  |  |  |
| Negative | 69 | 8 | NR | 0.214 |  |  |  |
| Positive | 10 | 2 | NR |  | 2.61 | (0.54-12.58) | 0.232 |
| Del(7q) |  |  |  |  |  |  |  |
| Negative | 67 | 7 | NR | 0.017 |  |  |  |
| Positive | 12 | 3 | NR |  | 4.69 | (1.17-18.80) | 0.029 |
| Trisomy 8 |  |  |  |  |  |  |  |
| Negative | 72 | 9 | NR | 0.761 |  |  |  |
| Positive | 7 | 1 | NR |  | 1.38 | (0.17-10.98) | 0.762 |
| Del12b |  |  |  |  |  |  |  |
| Negative | 73 | 7 | NR | 0.001 |  |  |  |
| Positive | 6 | 3 | 13.21 |  | 7.44 | (1.84-30.11) | 0.005 |
| Del(17p) |  |  |  |  |  |  |  |
| Negative | 75 | 8 | NR | 0.013 |  |  |  |
| Positive | 4 | 2 | 8.90 |  | 5.79 | (1.21-27.64) | 0.028 |
| Del(20q) |  |  |  |  |  |  |  |
| Negative | 71 | 9 | NR | 0.941 |  |  |  |
| Positive | 8 | 1 | NR |  | 1.08 | (0.14-8.58) | 0.941 |
| ASXL1 |  |  |  |  |  |  |  |
| Negative | 70 | 8 | NR | 0.730 |  |  |  |
| Positive | 13 | 2 | NR |  | 1.31 | (0.28-6.22) | 0.730 |
| BCOR |  |  |  |  |  |  |  |
| Negative | 79 | 10 | NR | 0.502 |  |  |  |
| Positive | 4 | 0 | NR |  | - | - | - |
| CUX1 |  |  |  |  |  |  |  |
| Negative | 80 | 10 | NR | 0.452 |  |  |  |
| Positive | 3 | 0 | NR |  | - | - | - |
| DNMT3A |  |  |  |  |  |  |  |
| Negative | 77 | 10 | NR | 0.395 |  |  |  |
| Positive | 6 | 0 | NR |  | - | - | - |
| ETV6 |  |  |  |  |  |  |  |
| Negative | 81 | 10 | NR | 0.581 |  |  |  |
| Positive | 2 | 0 | NR |  | - | - | - |
| EZH2 |  |  |  |  |  |  |  |
| Negative | 79 | 9 | NR | 0.145 |  |  |  |
| Positive | 4 | 1 | NR |  | 4.18 | (0.52-33.71) | 0.180 |
| NRAS |  |  |  |  |  |  |  |
| Negative | 77 | 8 | NR | 0.116 |  |  |  |
| Positive | 6 | 2 | 23.46 |  | 3.23 | (0.69-15.25) | 0.138 |
| RUNX1 |  |  |  |  |  |  |  |
| Negative | 69 | 9 | NR | 0.614 |  |  |  |
| Positive | 14 | 1 | NR |  | 0.59 | (0.07-4.67) | 0.618 |
| SETPB1 |  |  |  |  |  |  |  |
| Negative | 81 | 9 | NR | 0.268 |  |  |  |
| Positive | 2 | 1 | 26.15 |  | 3.05 | (0.38-24.39) | 0.293 |
| SF3B1b |  |  |  |  |  |  |  |
| Negative | 74 | 9 | NR | 0.894 |  |  |  |
| Positive | 9 | 1 | NR |  | 0.87 | (0.11-6.87) | 0.894 |
| SRSF2 |  |  |  |  |  |  |  |
| Negative | 69 | 9 | NR | 0.550 |  |  |  |
| Positive | 14 | 1 | NR |  | 0.54 | (0.07-4.25) | 0.556 |
| STAG2 |  |  |  |  |  |  |  |
| Negative | 73 | 8 | NR | 0.190 |  |  |  |
| Positive | 10 | 2 | NR |  | 2.74 | (0.57-13.14) | 0.208 |
| TET2 |  |  |  |  |  |  |  |
| Negative | 66 | 8 | NR | 0.938 |  |  |  |
| Positive | 17 | 2 | NR |  | 1.06 | (0.23-5.01) | 0.938 |
| TP53 |  |  |  |  |  |  |  |
| Negative | 75 | 7 | NR | <0.001 |  |  |  |
| Positive | 8 | 3 | NR |  | 8.96 | (2.10-38.12) | 0.003 |
| U2AF1 |  |  |  |  |  |  |  |
| Negative | 80 | 9 | NR | 0.466 |  |  |  |
| Positive | 3 | 1 | 26.15 |  | 2.12 | (0.27-16.88) | 0.476 |
| ZRSR2 |  |  |  |  |  |  |  |
| Negative | 76 | 8 | NR | 0.163 |  |  |  |
| Positive | 7 | 2 | 23.46 |  | 2.89 | (0.61-13.78) | 0.183 |
| Splicing pathway gene mutated |  |  |  |  |  |  |  |
| Negative | 50 | 5 | NR | 0.585 |  |  |  |
| Positive | 33 | 5 | NR |  | 1.41 | (0.41-4.88) | 0.587 |
| Methylation pathway gene mutated |  |  |  |  |  |  |  |
| Negative | 59 | 8 | NR | 0.520 |  |  |  |
| Positive | 24 | 2 | NR |  | 0.60 | (0.13-2.85) | 0.525 |
| Cohesin pathway gene mutated |  |  |  |  |  |  |  |
| Negative | 73 | 8 | NR | 0.190 |  |  |  |
| Positive | 10 | 2 | NR |  | 2.74 | (0.57-13.14) | 0.208 |
| Mutations>=4 |  |  |  |  |  |  |  |
| No | 71 | 8 | NR | 0.560 |  |  |  |
| Yes | 12 | 2 | NR |  | 1.58 | (0.33-7.46) | 0.564 |
| Mutations>=3 |  |  |  |  |  |  |  |
| No | 60 | 6 | NR | 0.342 |  |  |  |
| Yes | 23 | 4 | NR |  | 1.83 | (0.52-6.49) | 0.350 |
