# Supplementary material for: Impact of the number of mutations in survival and response outcomes to hypomethylating agents in patients with myelodysplastic syndromes or myelodysplastic/myeloproliferative neoplasms
Source: Oncotarget. 2018 Jan 3;9(11):9714–27. doi: 10.18632/oncotarget.23882 (PMC5839396; doi:10.18632/oncotarget.23882)
Supplement: Supplementary file 8 [file oncotarget-09-9714-s008.docx]

**Supplementary Table 7: Univariate analysis for leukemia-free survival in patients with MDS/MPN**

| Table S7. Univariate Analysis for LFS (CMML) | | | | | | | |
| --- | --- | --- | --- | --- | --- | --- | --- |
|  | N | Events | Median | log-rank | HR | 95% CI for HR | p-value |
| Age | 31 | 5 | NR |  | 0.81 | (0.66-1.00) | 0.049 |
| WBC | 31 | 5 | NR |  | 1.03 | (0.98-1.07) | 0.233 |
| ANC | 31 | 5 | NR |  | 1.05 | (0.99-1.12) | 0.099 |
| RBC | 31 | 5 | NR |  | 0.41 | (0.10-1.73) | 0.224 |
| PLT | 31 | 5 | NR |  | 0.99 | (0.97-1.01) | 0.176 |
| HGB | 31 | 5 | NR |  | 0.57 | (0.30-1.08) | 0.083 |
| NEUT | 31 | 5 | NR |  | 1.04 | (0.98-1.11) | 0.171 |
| PBBL | 31 | 5 | NR |  | 0.67 | (0.20-2.31) | 0.531 |
| BMBL | 30 | 5 | NR |  | 1.02 | (0.86-1.22) | 0.791 |
| Treatment |  |  |  |  |  |  |  |
| HMA | 21 | 3 | NR | 0.149 |  |  |  |
| AraC | 3 | 2 | 32.66 |  | 3.51 | (0.57-21.55) | 0.174 |
| Therapy Related |  |  |  |  |  |  |  |
| No | 28 | 4 | NR | 0.182 |  |  |  |
| Yes | 3 | 1 | 22.08 |  | 4.14 | (0.43-39.89) | 0.219 |
| IPSShl |  |  |  |  |  |  |  |
| Low/INT-1 | 21 | 3 | NR | 0.368 |  |  |  |
| INT-2/High | 10 | 2 | NR |  | 2.26 | (0.37-14.00) | 0.381 |
| MIPSS-R grouped |  |  |  |  |  |  |  |
| 0-0.5 | 14 | 2 | NR | 0.352 |  |  |  |
| 1-2 | 8 | 1 | NR |  | 1.30 | (0.11-15.03) | 0.833 |
| 2.5-3.5 | 9 | 2 | NR |  | 4.49 | (0.46-44.21) | 0.198 |
| IPSSb |  |  |  |  |  |  |  |
| 0 | 16 | 3 | NR | 0.452 |  |  |  |
| 1 | 9 | 2 | NR |  | 1.98 | (0.32-12.21) | 0.460 |
| IPSSRHIGH |  |  |  |  |  |  |  |
| VL/L/I | 21 | 3 | NR | 0.398 |  |  |  |
| V/VH | 10 | 2 | NR |  | 2.16 | (0.35-13.43) | 0.408 |
| ANC<0.80 |  |  |  |  |  |  |  |
| No | 29 | 5 | NR | 0.470 |  |  |  |
| Yes | 2 | 0 | NR |  | - | - | - |
| PLT<50 |  |  |  |  |  |  |  |
| No | 22 | 3 | NR | 0.245 |  |  |  |
| Yes | 9 | 2 | NR |  | 3.08 | (0.42-22.68) | 0.269 |
| Hgb<8 |  |  |  |  |  |  |  |
| No | 30 | 4 | NR | <0.001 |  |  |  |
| Yes | 1 | 1 | NR |  | - | - | - |
| BMBL>10 |  |  |  |  |  |  |  |
| No | 26 | 5 | NR | 0.373 |  |  |  |
| Yes | 4 | 0 | NR |  | - | - | - |
| Normal Karyotype |  |  |  |  |  |  |  |
| No | 12 | 1 | NR | 0.617 |  |  |  |
| Yes | 19 | 4 | NR |  | 1.75 | (0.19-16.20) | 0.621 |
| CGHIGHb |  |  |  |  |  |  |  |
| No | 28 | 5 | NR | 0.593 |  |  |  |
| Yes | 3 | 0 | NR |  | - | - | - |
| Complex karyotype |  |  |  |  |  |  |  |
| No | 29 | 5 | NR | 0.694 |  |  |  |
| Yes | 2 | 0 | NR |  | - | - | - |
| CGMKb |  |  |  |  |  |  |  |
| No | 30 | 5 | NR | 0.853 |  |  |  |
| Yes | 1 | 0 | NR |  | - | - | - |
|  |  |  |  |  |  |  |  |
|  |  |  |  |  |  |  |  |
| Chr-Y |  |  |  |  |  |  |  |
| Negative | 30 | 5 | NR | 0.853 |  |  |  |
| Positive | 1 | 0 | NR |  | - | - | - |
| Del(5q) |  |  |  |  |  |  |  |
| Negative | 30 | 5 | NR | 0.853 |  |  |  |
| Positive | 1 | 0 | NR |  | - | - | - |
| Del(7q) |  |  |  |  |  |  |  |
| Negative | 30 | 5 | NR | 0.731 |  |  |  |
| Positive | 1 | 0 | NR |  | - | - | - |
| Trisomy 8 |  |  |  |  |  |  |  |
| Negative | 27 | 5 | NR | 0.342 |  |  |  |
| Positive | 4 | 0 | NR |  | - | - | - |
| Del(17p) |  |  |  |  |  |  |  |
| Negative | 29 | 5 | NR | 0.630 |  |  |  |
| Positive | 2 | 0 | NR |  | - | - | - |
| Del(20q) |  |  |  |  |  |  |  |
| Negative | 29 | 5 | NR | 0.789 |  |  |  |
| Positive | 2 | 0 | NR |  | - | - | - |
| ASXL1 |  |  |  |  |  |  |  |
| Negative | 21 | 3 | NR | 0.595 |  |  |  |
| Positive | 10 | 2 | NR |  | 1.63 | (0.27-9.98) | 0.599 |
| BCOR |  |  |  |  |  |  |  |
| Negative | 29 | 4 | NR | 0.050 |  |  |  |
| Positive | 2 | 1 | 8.77 |  | 7.74 | (0.69-87.04) | 0.097 |
| CUX1 |  |  |  |  |  |  |  |
| Negative | 29 | 5 | NR | 0.694 |  |  |  |
| Positive | 2 | 0 | NR |  | - | - | - |
| DNMT3A |  |  |  |  |  |  |  |
| Negative | 30 | 5 | NR | 0.731 |  |  |  |
| Positive | 1 | 0 | NR |  | - | - | - |
| ETV6 |  |  |  |  |  |  |  |
| Negative | 28 | 4 | NR | 0.332 |  |  |  |
| Positive | 3 | 1 | NR |  | 2.93 | (0.30-28.43) | 0.355 |
| EZH2 |  |  |  |  |  |  |  |
| Negative | 30 | 4 | NR | <0.001 |  |  |  |
| Positive | 1 | 1 | NR |  | - | - | - |
| NRAS |  |  |  |  |  |  |  |
| Negative | 29 | 4 | NR | 0.406 |  |  |  |
| Positive | 2 | 1 | 9.86 |  | 2.49 | (0.27-22.97) | 0.422 |
| RUNX1 |  |  |  |  |  |  |  |
| Negative | 29 | 5 | NR | 0.620 |  |  |  |
| Positive | 2 | 0 | NR |  | - | - | - |
| SETPB1 |  |  |  |  |  |  |  |
| Negative | 28 | 4 | NR | 0.225 |  |  |  |
| Positive | 3 | 1 | 9.86 |  | 3.70 | (0.38-35.71) | 0.258 |
| SRSF2 |  |  |  |  |  |  |  |
| Negative | 18 | 2 | NR | 0.252 |  |  |  |
| Positive | 13 | 3 | 32.66 |  | 2.83 | (0.45-17.94) | 0.270 |
| TET2 |  |  |  |  |  |  |  |
| Negative | 15 | 4 | NR | 0.269 |  |  |  |
| Positive | 16 | 1 | NR |  | 0.30 | (0.03-2.83) | 0.295 |
| TP53 |  |  |  |  |  |  |  |
| Negative | 29 | 5 | NR | 0.694 |  |  |  |
| Positive | 2 | 0 | NR |  | - | - | - |
| U2AF1 |  |  |  |  |  |  |  |
| Negative | 28 | 4 | NR | 0.451 |  |  |  |
| Positive | 3 | 1 | NR |  | 2.29 | (0.25-21.03) | 0.463 |
| ZRSR2 |  |  |  |  |  |  |  |
| Negative | 28 | 5 | NR | 0.521 |  |  |  |
| Positive | 3 | 0 | NR |  | - | - | - |
| Splicing pathway gene mutated |  |  |  |  |  |  |  |
| Negative | 12 | 1 | NR | 0.222 |  |  |  |
| Positive | 19 | 4 | 32.66 |  | 3.70 | (0.40-34.44) | 0.251 |
| Methylation pathway gene mutated |  |  |  |  |  |  |  |
| Negative | 14 | 4 | NR | 0.255 |  |  |  |
| Positive | 17 | 1 | NR |  | 0.29 | (0.03-2.74) | 0.282 |
| Mutations>=4 |  |  |  |  |  |  |  |
| No | 25 | 2 | NR | 0.002 |  |  |  |
| Yes | 6 | 3 | 22.08 |  | 15.29 | (1.58-147.71) | 0.018 |
| Mutations>=3 |  |  |  |  |  |  |  |
| No | 16 | 2 | NR | 0.184 |  |  |  |
| Yes | 15 | 3 | NR |  | 4.21 | (0.42-41.81) | 0.219 |
