# Supplementary material for: Impact of the number of mutations in survival and response outcomes to hypomethylating agents in patients with myelodysplastic syndromes or myelodysplastic/myeloproliferative neoplasms
Source: Oncotarget. 2018 Jan 3;9(11):9714–27. doi: 10.18632/oncotarget.23882 (PMC5839396; doi:10.18632/oncotarget.23882)
Supplement: Supplementary file 9 [file oncotarget-09-9714-s009.docx]

**Supplementary Table 9: Univariate analysis for response outcomes after hypomethylating agent therapy**

| Table S9a. Logistic Regression Models for ORR | | | | | | | |
| --- | --- | --- | --- | --- | --- | --- | --- |
|  | No | | Yes | | OR | 95% CI for OR | p-value |
| Continuous | Mean | SD | Mean | SD |  |  |  |
| Age | 65.4 | (10.3) | 64.2 | (13.0) | 0.99 | 0.95-1.04 | 0.693 |
| PLT | 127.9 | (133.4) | 89.5 | (80.9) | 1.00 | 0.99-1.00 | 0.188 |
| Hgb | 9.8 | (2.0) | 10.6 | (2.1) | 1.20 | 0.93-1.56 | 0.165 |
| Neut | 47.3 | (19.0) | 48.7 | (22.7) | 1.00 | 0.98-1.03 | 0.780 |
| ANC | 5.6 | (11.1) | 6.2 | (9.2) | 1.01 | 0.96-1.06 | 0.821 |
| BM Blast | 6.9 | (5.2) | 7.0 | (4.2) | 1.00 | 0.90-1.12 | 0.934 |
| NUM MUT | 1.3 | (1.3) | 1.0 | (0.9) | 0.74 | 0.47-1.19 | 0.214 |
| Categorical | N | % | N | % |  |  |  |
| Gender |  |  |  |  |  |  |  |
| Female | 10 | 33.33 | 5 | 16.13 |  |  |  |
| Male | 20 | 66.67 | 26 | 83.87 | 2.60 | (0.77-8.82) | 0.125 |
| TMDS |  |  |  |  |  |  |  |
| No | 23 | 76.67 | 27 | 87.10 |  |  |  |
| Yes | 7 | 23.33 | 4 | 12.90 | 0.49 | (0.13-1.87) | 0.295 |
| CMML |  |  |  |  |  |  |  |
| No | 23 | 76.67 | 20 | 64.52 |  |  |  |
| Yes | 7 | 23.33 | 11 | 35.48 | 1.81 | (0.59-5.55) | 0.301 |
| High risk |  |  |  |  |  |  |  |
| No | 15 | 50.00 | 17 | 54.84 |  |  |  |
| Yes | 15 | 50.00 | 14 | 45.16 | 0.82 | (0.30-2.25) | 0.705 |
| IPSS Group |  |  |  |  |  |  |  |
| 1 | 2 | 6.67 | 1 | 3.23 |  |  |  |
| 2 | 13 | 43.33 | 16 | 51.61 | 2.46 | (0.20-30.28) | 0.482 |
| 3 | 11 | 36.67 | 13 | 41.94 | 2.36 | (0.19-29.71) | 0.505 |
| 4 | 4 | 13.33 | 1 | 3.23 | 0.50 | (0.02-12.90) | 0.676 |
| IPSS-R Group |  |  |  |  |  |  |  |
| 1 | 2 | 6.67 | 1 | 3.23 |  |  |  |
| 2 | 3 | 10.00 | 2 | 6.45 | 1.33 | (0.07-26.62) | 0.851 |
| 3 | 12 | 40.00 | 12 | 38.71 | 2.00 | (0.16-25.12) | 0.591 |
| 4 | 6 | 20.00 | 12 | 38.71 | 4.00 | (0.30-53.47) | 0.295 |
| 5 | 7 | 23.33 | 4 | 12.90 | 1.14 | (0.08-16.95) | 0.923 |
| PS |  |  |  |  |  |  |  |
| 0 | 11 | 36.67 | 12 | 38.71 |  |  |  |
| 1 | 16 | 53.33 | 18 | 58.06 | 1.03 | (0.36-2.98) | 0.955 |
| 2-3 | 3 | 10.00 | 1 | 3.23 | 0.31 | (0.03-3.39) | 0.334 |
| CG Complex |  |  |  |  |  |  |  |
| No | 21 | 72.41 | 24 | 80.00 |  |  |  |
| Yes | 8 | 27.59 | 6 | 20.00 | 0.66 | (0.20-2.20) | 0.495 |
| Diploid |  |  |  |  |  |  |  |
| No | 18 | 62.07 | 16 | 53.33 |  |  |  |
| Yes | 11 | 37.93 | 14 | 46.67 | 1.43 | (0.51-4.04) | 0.498 |
| IPSS-R CG |  |  |  |  |  |  |  |
| 0 | 1 | 3.45 | 1 | 3.33 |  |  |  |
| 1 | 14 | 48.28 | 16 | 53.33 | 1.14 | (0.07-20.02) | 0.927 |
| 2 | 7 | 24.14 | 7 | 23.33 | 1.00 | (0.05-19.36) | 0.999 |
| 3 | 0 | 0.00 | 1 | 3.33 |  |  |  |
| 4 | 7 | 24.14 | 5 | 16.67 | 0.71 | (0.04-14.35) | 0.826 |
| Plt<100 |  |  |  |  |  |  |  |
| No | 12 | 40.00 | 8 | 25.81 |  |  |  |
| Yes | 18 | 60.00 | 23 | 74.19 | 1.92 | (0.65-5.68) | 0.241 |
| Plt<50 |  |  |  |  |  |  |  |
| No | 20 | 66.67 | 21 | 67.74 |  |  |  |
| Yes | 10 | 33.33 | 10 | 32.26 | 0.95 | (0.33-2.77) | 0.929 |
| ASXL1 |  |  |  |  |  |  |  |
| No | 23 | 76.67 | 27 | 87.10 |  |  |  |
| Yes | 7 | 23.33 | 4 | 12.90 | 0.49 | (0.13-1.87) | 0.295 |
| RUNX1 |  |  |  |  |  |  |  |
| No | 24 | 80.00 | 28 | 90.32 |  |  |  |
| Yes | 6 | 20.00 | 3 | 9.68 | 0.43 | (0.10-1.90) | 0.265 |
| TET2 |  |  |  |  |  |  |  |
| No | 22 | 73.33 | 23 | 74.19 |  |  |  |
| Yes | 8 | 26.67 | 8 | 25.81 | 0.96 | (0.31-2.99) | 0.939 |
| TP53 |  |  |  |  |  |  |  |
| No | 25 | 83.33 | 27 | 87.10 |  |  |  |
| Yes | 5 | 16.67 | 4 | 12.90 | 0.74 | (0.18-3.07) | 0.679 |
| Mutation 1 or more |  |  |  |  |  |  |  |
| No | 4 | 13.33 | 5 | 16.13 |  |  |  |
| Yes | 26 | 86.67 | 26 | 83.87 | 0.80 | (0.19-3.32) | 0.759 |
| Mutation 2 or more |  |  |  |  |  |  |  |
| No | 14 | 46.67 | 17 | 54.84 |  |  |  |
| Yes | 16 | 53.33 | 14 | 45.16 | 0.72 | (0.26-1.97) | 0.524 |
| Mutation 3 or more |  |  |  |  |  |  |  |
| No | 15 | 50.00 | 23 | 74.19 |  |  |  |
| Yes | 15 | 50.00 | 8 | 25.81 | 0.35 | (0.12-1.02) | 0.055 |
| Mutation 4 or more |  |  |  |  |  |  |  |
| No | 22 | 73.33 | 29 | 93.55 |  |  |  |
| Yes | 8 | 26.67 | 2 | 6.45 | 0.19 | (0.04-0.98) | 0.048 |
| BCOR |  |  |  |  |  |  |  |
| No | 27 | 90.00 | 29 | 93.55 |  |  |  |
| Yes | 3 | 10.00 | 2 | 6.45 | 0.62 | (0.10-4.00) | 0.616 |
| SRSF2 |  |  |  |  |  |  |  |
| No | 21 | 70.00 | 27 | 87.10 |  |  |  |
| Yes | 9 | 30.00 | 4 | 12.90 | 0.35 | (0.09-1.28) | 0.112 |
| STAG2 |  |  |  |  |  |  |  |
| No | 26 | 86.67 | 29 | 93.55 |  |  |  |
| Yes | 4 | 13.33 | 2 | 6.45 | 0.45 | (0.08-2.65) | 0.376 |
| U2AF1 |  |  |  |  |  |  |  |
| No | 28 | 93.33 | 29 | 93.55 |  |  |  |
| Yes | 2 | 6.67 | 2 | 6.45 | 0.97 | (0.13-7.33) | 0.973 |
| ZRSR2 |  |  |  |  |  |  |  |
| No | 28 | 93.33 | 28 | 90.32 |  |  |  |
| Yes | 2 | 6.67 | 3 | 9.68 | 1.50 | (0.23-9.68) | 0.670 |
| Splicing |  |  |  |  |  |  |  |
| No | 15 | 50.00 | 22 | 70.97 |  |  |  |
| Yes | 15 | 50.00 | 9 | 29.03 | 0.41 | (0.14-1.18) | 0.097 |
| DNA methylation |  |  |  |  |  |  |  |
| No | 22 | 73.33 | 20 | 64.52 |  |  |  |
| Yes | 8 | 26.67 | 11 | 35.48 | 1.51 | (0.51-4.52) | 0.458 |
| Cohesins |  |  |  |  |  |  |  |
| No | 26 | 86.67 | 29 | 93.55 |  |  |  |
| Yes | 4 | 13.33 | 2 | 6.45 | 0.45 | (0.08-2.65) | 0.376 |
| Chromatine |  |  |  |  |  |  |  |
| No | 23 | 76.67 | 27 | 87.10 |  |  |  |
| Yes | 7 | 23.33 | 4 | 12.90 | 0.49 | (0.13-1.87) | 0.295 |
| Transcription Factors |  |  |  |  |  |  |  |
| No | 18 | 60.00 | 22 | 70.97 |  |  |  |
| Yes | 12 | 40.00 | 9 | 29.03 | 0.61 | (0.21-1.78) | 0.369 |
| Signaling |  |  |  |  |  |  |  |
| No | 26 | 86.67 | 27 | 87.10 |  |  |  |
| Yes | 4 | 13.33 | 4 | 12.90 | 0.96 | (0.22-4.26) | 0.960 |
| DNA damage |  |  |  |  |  |  |  |
| No | 24 | 80.00 | 27 | 87.10 |  |  |  |
| Yes | 6 | 20.00 | 4 | 12.90 | 0.59 | (0.15-2.35) | 0.457 |
| Other |  |  |  |  |  |  |  |
| No | 29 | 96.67 | 30 | 96.77 |  |  |  |
| Yes | 1 | 3.33 | 1 | 3.23 | 0.97 | (0.06-16.19) | 0.981 |

| Table S9b. Logistic Regression Models for CR | | | | | | | |
| --- | --- | --- | --- | --- | --- | --- | --- |
|  | No | | Yes | | OR | 95% CI for OR | p-value |
| Continuous | Mean | SD | Mean | SD |  |  |  |
| Age | 64.6 | (12.9) | 65.3 | (7.6) | 1.01 | 0.96-1.06 | 0.832 |
| PLT | 113.1 | (121.3) | 94.9 | (74.8) | 1.00 | 0.99-1.00 | 0.573 |
| Hgb | 9.8 | (2.0) | 11.2 | (2.0) | 1.38 | 1.03-1.84 | 0.030 |
| Neut | 49.4 | (20.6) | 44.1 | (21.5) | 0.99 | 0.96-1.02 | 0.385 |
| ANC | 6.8 | (11.3) | 3.4 | (4.8) | 0.95 | 0.88-1.04 | 0.273 |
| BM Blast | 6.4 | (4.9) | 8.7 | (3.6) | 1.11 | 0.98-1.25 | 0.096 |
| NUM MUT | 1.3 | (1.2) | 0.6 | (0.6) | 0.47 | 0.23-0.96 | 0.039 |
| Categorical | N | % | N | % |  |  |  |
| Gender |  |  |  |  |  |  |  |
| Female | 12 | 26.67 | 3 | 18.75 |  |  |  |
| Male | 33 | 73.33 | 13 | 81.25 | 1.58 | (0.38-6.51) | 0.530 |
| TMDS |  |  |  |  |  |  |  |
| No | 35 | 77.78 | 15 | 93.75 |  |  |  |
| Yes | 10 | 22.22 | 1 | 6.25 | 0.23 | (0.03-1.99) | 0.183 |
| CMML |  |  |  |  |  |  |  |
| No | 34 | 75.56 | 9 | 56.25 |  |  |  |
| Yes | 11 | 24.44 | 7 | 43.75 | 2.40 | (0.72-7.97) | 0.152 |
| High risk |  |  |  |  |  |  |  |
| No | 23 | 51.11 | 9 | 56.25 |  |  |  |
| Yes | 22 | 48.89 | 7 | 43.75 | 0.81 | (0.26-2.56) | 0.724 |
| IPSS Group |  |  |  |  |  |  |  |
| 1 | 3 | 6.67 | 0 | 0.00 |  |  |  |
| 2 | 20 | 44.44 | 9 | 56.25 | 1.80 | (0.18-18.47) | 0.621 |
| 3 | 18 | 40.00 | 6 | 37.50 | 1.33 | (0.12-14.38) | 0.813 |
| 4 | 4 | 8.89 | 1 | 6.25 | - | - | - |
| IPSS-R Group |  |  |  |  |  |  |  |
| 1 | 3 | 6.67 | 0 | 0.00 |  |  |  |
| 2 | 4 | 8.89 | 1 | 6.25 | 1.12 | (0.08-16.31) | 0.931 |
| 3 | 18 | 40.00 | 6 | 37.50 | 1.50 | (0.25-8.98) | 0.657 |
| 4 | 11 | 24.44 | 7 | 43.75 | 2.86 | (0.47-17.35) | 0.252 |
| 5 | 9 | 20.00 | 2 | 12.50 | - | - | - |
| PS |  |  |  |  |  |  |  |
| 0 | 14 | 31.11 | 9 | 56.25 |  |  |  |
| 1 | 27 | 60.00 | 7 | 43.75 | 0.40 | (0.12-1.31) | 0.131 |
| 2-3 | 4 | 8.89 | 0 | 0.00 | - | - | - |
| CG Complex |  |  |  |  |  |  |  |
| No | 31 | 72.09 | 14 | 87.50 |  |  |  |
| Yes | 12 | 27.91 | 2 | 12.50 | 0.37 | (0.07-1.87) | 0.229 |
| Diploid |  |  |  |  |  |  |  |
| No | 28 | 65.12 | 6 | 37.50 |  |  |  |
| Yes | 15 | 34.88 | 10 | 62.50 | 3.11 | (0.95-10.23) | 0.062 |
| IPSS-R CG |  |  |  |  |  |  |  |
| 0 | 2 | 4.65 | 0 | 0.00 |  |  |  |
| 1 | 18 | 41.86 | 12 | 75.00 | 3.33 | (0.62-17.97) | 0.161 |
| 2 | 12 | 27.91 | 2 | 12.50 | 0.83 | (0.10-7.03) | 0.867 |
| 3 | 1 | 2.33 | 0 | 0.00 | - | - | - |
| 4 | 10 | 23.26 | 2 | 12.50 | - | - | - |
| Plt<100 |  |  |  |  |  |  |  |
| No | 15 | 33.33 | 5 | 31.25 |  |  |  |
| Yes | 30 | 66.67 | 11 | 68.75 | 1.10 | (0.32-3.75) | 0.879 |
| Plt<50 |  |  |  |  |  |  |  |
| No | 29 | 64.44 | 12 | 75.00 |  |  |  |
| Yes | 16 | 35.56 | 4 | 25.00 | 0.60 | (0.17-2.19) | 0.442 |
| ASXL1 |  |  |  |  |  |  |  |
| No | 36 | 80.00 | 14 | 87.50 |  |  |  |
| Yes | 9 | 20.00 | 2 | 12.50 | 0.57 | (0.11-2.98) | 0.507 |
| RUNX1 |  |  |  |  |  |  |  |
| No | 36 | 80.00 | 16 | 100.00 |  |  |  |
| Yes | 9 | 20.00 | 0 | 0.00 | - | - | - |
| TET2 |  |  |  |  |  |  |  |
| No | 32 | 71.11 | 13 | 81.25 |  |  |  |
| Yes | 13 | 28.89 | 3 | 18.75 | 0.57 | (0.14-2.33) | 0.432 |
| TP53 |  |  |  |  |  |  |  |
| No | 38 | 84.44 | 14 | 87.50 |  |  |  |
| Yes | 7 | 15.56 | 2 | 12.50 | 0.78 | (0.14-4.19) | 0.768 |
| Mutation 1 or more |  |  |  |  |  |  |  |
| No | 5 | 11.11 | 4 | 25.00 |  |  |  |
| Yes | 40 | 88.89 | 12 | 75.00 | 0.37 | (0.09-1.62) | 0.189 |
| Mutation 2 or more |  |  |  |  |  |  |  |
| No | 22 | 48.89 | 9 | 56.25 |  |  |  |
| Yes | 23 | 51.11 | 7 | 43.75 | 0.74 | (0.24-2.34) | 0.613 |
| Mutation 3 or more |  |  |  |  |  |  |  |
| No | 25 | 55.56 | 13 | 81.25 |  |  |  |
| Yes | 20 | 44.44 | 3 | 18.75 | 0.29 | (0.07-1.15) | 0.079 |
| Mutation 4 or more |  |  |  |  |  |  |  |
| No | 36 | 80.00 | 15 | 93.75 |  |  |  |
| Yes | 9 | 20.00 | 1 | 6.25 | 0.27 | (0.03-2.29) | 0.229 |
| BCOR |  |  |  |  |  |  |  |
| No | 41 | 91.11 | 15 | 93.75 |  |  |  |
| Yes | 4 | 8.89 | 1 | 6.25 | 0.68 | (0.07-6.61) | 0.742 |
| CBL |  |  |  |  |  |  |  |
| No | 43 | 95.56 | 15 | 93.75 |  |  |  |
| Yes | 2 | 4.44 | 1 | 6.25 | 1.43 | (0.12-16.97) | 0.775 |
| CUX1 |  |  |  |  |  |  |  |
| No | 44 | 97.78 | 15 | 93.75 |  |  |  |
| Yes | 1 | 2.22 | 1 | 6.25 | 2.93 | (0.17-49.86) | 0.457 |
| DDX41 |  |  |  |  |  |  |  |
| No | 45 | 100.00 | 15 | 93.75 |  |  |  |
| Yes | 0 | 0.00 | 1 | 6.25 | - | - | - |
| ETNK1 |  |  |  |  |  |  |  |
| No | 44 | 97.78 | 16 | 100.00 |  |  |  |
| Yes | 1 | 2.22 | 0 | 0.00 | - | - | - |
| ETV6 |  |  |  |  |  |  |  |
| No | 43 | 95.56 | 15 | 93.75 |  |  |  |
| Yes | 2 | 4.44 | 1 | 6.25 | 1.43 | (0.12-16.97) | 0.775 |
| NF1 |  |  |  |  |  |  |  |
| No | 45 | 100.00 | 15 | 93.75 |  |  |  |
| Yes | 0 | 0.00 | 1 | 6.25 | - | - | - |
| PHF6 |  |  |  |  |  |  |  |
| No | 44 | 97.78 | 16 | 100.00 |  |  |  |
| Yes | 1 | 2.22 | 0 | 0.00 | - | - | - |
| SETBP1 |  |  |  |  |  |  |  |
| No | 43 | 95.56 | 15 | 93.75 |  |  |  |
| Yes | 2 | 4.44 | 1 | 6.25 | 1.43 | (0.12-16.97) | 0.775 |
| SRSF2 |  |  |  |  |  |  |  |
| No | 35 | 77.78 | 13 | 81.25 |  |  |  |
| Yes | 10 | 22.22 | 3 | 18.75 | 0.81 | (0.19-3.40) | 0.771 |
| STAG2 |  |  |  |  |  |  |  |
| No | 39 | 86.67 | 16 | 100.00 |  |  |  |
| Yes | 6 | 13.33 | 0 | 0.00 | - | - | - |
| U2AF1 |  |  |  |  |  |  |  |
| No | 42 | 93.33 | 15 | 93.75 |  |  |  |
| Yes | 3 | 6.67 | 1 | 6.25 | 0.93 | (0.09-9.68) | 0.954 |
| ZRSR2 |  |  |  |  |  |  |  |
| No | 42 | 93.33 | 14 | 87.50 |  |  |  |
| Yes | 3 | 6.67 | 2 | 12.50 | 2.00 | (0.30-13.22) | 0.472 |
| Splicing |  |  |  |  |  |  |  |
| No | 27 | 60.00 | 10 | 62.50 |  |  |  |
| Yes | 18 | 40.00 | 6 | 37.50 | 0.90 | (0.28-2.91) | 0.860 |
| DNA methylation |  |  |  |  |  |  |  |
| No | 30 | 66.67 | 12 | 75.00 |  |  |  |
| Yes | 15 | 33.33 | 4 | 25.00 | 0.67 | (0.18-2.42) | 0.538 |
| Cohesins |  |  |  |  |  |  |  |
| No | 39 | 86.67 | 16 | 100.00 |  |  |  |
| Yes | 6 | 13.33 | 0 | 0.00 | - | - | - |
| Chromatine |  |  |  |  |  |  |  |
| No | 36 | 80.00 | 14 | 87.50 |  |  |  |
| Yes | 9 | 20.00 | 2 | 12.50 | 0.57 | (0.11-2.98) | 0.507 |
| Transcription Factors |  |  |  |  |  |  |  |
| No | 28 | 62.22 | 12 | 75.00 |  |  |  |
| Yes | 17 | 37.78 | 4 | 25.00 | 0.55 | (0.15-1.98) | 0.359 |
| Signaling |  |  |  |  |  |  |  |
| No | 38 | 84.44 | 15 | 93.75 |  |  |  |
| Yes | 7 | 15.56 | 1 | 6.25 | 0.36 | (0.04-3.20) | 0.361 |
| DNA damage |  |  |  |  |  |  |  |
| No | 37 | 82.22 | 14 | 87.50 |  |  |  |
| Yes | 8 | 17.78 | 2 | 12.50 | 0.66 | (0.12-3.50) | 0.626 |
| other |  |  |  |  |  |  |  |
| No | 44 | 97.78 | 15 | 93.75 |  |  |  |
| Yes | 1 | 2.22 | 1 | 6.25 | 2.93 | (0.17-49.86) | 0.457 |

| Table S9c. Univariate Competing Risk for Time to Response | | | | | | |
| --- | --- | --- | --- | --- | --- | --- |
|  | N | Loss of Response | Relapse or Death | HR | 95%CI | p-value |
| Age | 51 | 30 | 17 | 0.99 | (0.97-1.01) | 0.432 |
| PLT | 51 | 30 | 17 | 1.00 | (0.99-1.00) | 0.408 |
| Hgb | 51 | 30 | 17 | 1.09 | (0.91-1.31) | 0.354 |
| Neut | 51 | 30 | 17 | 1.00 | (0.99-1.02) | 0.887 |
| ANC | 51 | 30 | 17 | 1.00 | (0.97-1.03) | 0.986 |
| BM Blast | 51 | 30 | 17 | 1.01 | (0.94-1.08) | 0.867 |
| NUM MUT | 51 | 30 | 17 | 0.91 | (0.65-1.28) | 0.590 |
| Gender |  |  |  |  |  |  |
| Female | 9 | 4 | 3 |  |  |  |
| Male | 42 | 26 | 16 | 1.45 | (0.54-3.87) | 0.462 |
| TMDS |  |  |  |  |  |  |
| No | 42 | 26 | 14 |  |  |  |
| Yes | 9 | 4 | 5 | 0.60 | (0.20-1.75) | 0.346 |
| CMML |  |  |  |  |  |  |
| No | 36 | 20 | 14 |  |  |  |
| Yes | 15 | 10 | 5 | 1.16 | (0.57-2.38) | 0.686 |
| High risk |  |  |  |  |  |  |
| No | 25 | 16 | 8 |  |  |  |
| Yes | 26 | 14 | 11 | 0.82 | (0.40-1.67) | 0.584 |
| IPSS Group |  |  |  |  |  |  |
| 1 | 3 | 1 | 0 |  |  |  |
| 2 | 22 | 15 | 6 | 3.72 | (0.73-19.11) | 0.115 |
| 3 | 22 | 13 | 8 | 3.02 | (0.55-16.45) | 0.201 |
| 4 | 4 | 1 | 3 | 0.91 | (0.07-11.87) | 0.943 |
| IPSS-R Group |  |  |  |  |  |  |
| 1 | 3 | 1 | 0 |  |  |  |
| 2 | 3 | 2 | 2 | 3.54 | (0.43-28.77) | 0.238 |
| 3 | 19 | 11 | 4 | 2.96 | (0.56-15.59) | 0.200 |
| 4 | 16 | 12 | 5 | 4.96 | (0.89-27.59) | 0.067 |
| 5 | 10 | 4 | 6 | 1.49 | (0.23-9.52) | 0.674 |
| PS |  |  |  |  |  |  |
| 0 | 20 | 12 | 5 |  |  |  |
| 1 | 27 | 17 | 11 | 0.80 | (0.38-1.71) | 0.570 |
| 2-3 | 4 | 1 | 0 | 0.28 | (0.03-2.77) | 0.278 |
| CG Complex |  |  |  |  |  |  |
| No | 37 | 23 | 12 |  |  |  |
| Yes | 13 | 6 | 6 | 0.63 | (0.27-1.48) | 0.289 |
| Diploid |  |  |  |  |  |  |
| No | 32 | 16 | 13 |  |  |  |
| Yes | 18 | 13 | 5 | 1.79 | (0.87-3.68) | 0.112 |
| IPSS-R CG |  |  |  |  |  |  |
| 0 | 2 | 1 | 0 |  |  |  |
| 1 | 22 | 15 | 7 | 2.02 | (0.38-10.60) | 0.407 |
| 2 | 13 | 7 | 4 | 1.41 | (0.25-7.92) | 0.699 |
| 3 | 1 | 1 | 0 | 2.84 | (0.61-13.16) | 0.183 |
| 4 | 12 | 5 | 6 | 0.96 | (0.16-5.77) | 0.962 |
| Plt<100 |  |  |  |  |  |  |
| No | 15 | 8 | 7 |  |  |  |
| Yes | 36 | 22 | 12 | 1.37 | (0.64-2.92) | 0.422 |
| Plt<50 |  |  |  |  |  |  |
| No | 34 | 21 | 10 |  |  |  |
| Yes | 17 | 9 | 9 | 0.74 | (0.34-1.60) | 0.443 |
| ASXL1 |  |  |  |  |  |  |
| No | 42 | 26 | 14 |  |  |  |
| Yes | 9 | 4 | 5 | 0.62 | (0.22-1.74) | 0.365 |
| RUNX1 |  |  |  |  |  |  |
| No | 44 | 27 | 15 |  |  |  |
| Yes | 7 | 3 | 4 | 0.59 | (0.20-1.78) | 0.348 |
| TET2 |  |  |  |  |  |  |
| No | 39 | 23 | 14 |  |  |  |
| Yes | 12 | 7 | 5 | 0.81 | (0.37-1.78) | 0.598 |
| TP53 |  |  |  |  |  |  |
| No | 42 | 26 | 14 |  |  |  |
| Yes | 9 | 4 | 0 | 0.63 | (0.21-1.87) | 0.407 |
| Mutation 1 or more |  |  |  |  |  |  |
| No | 9 | 5 | 3 |  |  |  |
| Yes | 42 | 25 | 16 | 1.06 | (0.39-2.85) | 0.908 |
| Mutation 2 or more |  |  |  |  |  |  |
| No | 28 | 17 | 8 |  |  |  |
| Yes | 23 | 13 | 11 | 0.88 | (0.43-1.81) | 0.734 |
| Mutation 3 or more |  |  |  |  |  |  |
| No | 34 | 22 | 9 |  |  |  |
| Yes | 17 | 8 | 10 | 0.64 | (0.27-1.47) | 0.291 |
| Mutation 4 or more |  |  |  |  |  |  |
| No | 46 | 28 | 15 |  |  |  |
| Yes | 5 | 2 | 4 | 0.66 | (0.12-3.65) | 0.631 |
| BCOR |  |  |  |  |  |  |
| No | 47 | 28 | 17 |  |  |  |
| Yes | 4 | 2 | 2 | 0.93 | (0.17-4.96) | 0.931 |
| SRSF2 |  |  |  |  |  |  |
| No | 43 | 27 | 14 |  |  |  |
| Yes | 8 | 3 | 5 | 0.47 | (0.14-1.64) | 0.236 |
| STAG2 |  |  |  |  |  |  |
| No | 48 | 28 | 17 |  |  |  |
| Yes | 3 | 2 | 2 | 1.15 | (0.27-4.83) | 0.853 |
| U2AF1 |  |  |  |  |  |  |
| No | 47 | 28 | 17 |  |  |  |
| Yes | 4 | 2 | 0 | 0.64 | (0.18-2.23) | 0.483 |
| ZRSR2 |  |  |  |  |  |  |
| No | 46 | 27 | 18 |  |  |  |
| Yes | 5 | 3 | 1 | 0.88 | (0.33-2.35) | 0.800 |
| Splicing |  |  |  |  |  |  |
| No | 34 | 22 | 11 |  |  |  |
| Yes | 17 | 8 | 8 | 0.54 | (0.25-1.16) | 0.115 |
| DNA methylation |  |  |  |  |  |  |
| No | 36 | 20 | 14 |  |  |  |
| Yes | 15 | 10 | 5 | 1.26 | (0.59-2.67) | 0.551 |
| Cohesins |  |  |  |  |  |  |
| No | 48 | 28 | 17 |  |  |  |
| Yes | 3 | 2 | 2 | 1.15 | (0.27-4.83) | 0.853 |
| Chromatine |  |  |  |  |  |  |
| No | 42 | 26 | 14 |  |  |  |
| Yes | 9 | 4 | 5 | 0.62 | (0.22-1.74) | 0.365 |
| Transcription Factors |  |  |  |  |  |  |
| No | 35 | 21 | 11 |  |  |  |
| Yes | 16 | 9 | 8 | 0.95 | (0.44-2.06) | 0.896 |
| Signaling |  |  |  |  |  |  |
| No | 45 | 26 | 17 |  |  |  |
| Yes | 6 | 4 | 2 | 1.10 | (0.43-2.81) | 0.843 |
| DNA damage |  |  |  |  |  |  |
| No | 41 | 26 | 13 |  |  |  |
| Yes | 10 | 4 | 0 | 0.52 | (0.18-1.56) | 0.245 |
| other |  |  |  |  |  |  |
| No | 49 | 29 | 18 |  |  |  |
| Yes | 2 | 1 | 0 | 1.09 | (0.07-16.07) | 0.952 |

| Table S9d. Univariate Analysis for Competing Risk for Time to Loss of Response | | | | | | |
| --- | --- | --- | --- | --- | --- | --- |
|  | N | Loss of Response | Relapse or Death | HR | 95%CI | p-value |
| Age | 41 | 18 | 0 | 1.00 | (0.98-1.02) | 0.981 |
| PLT | 41 | 18 | 0 | 1.00 | (1.00-1.01) | 0.252 |
| PLT divided by 100 | 41 | 18 | 0 | 1.63 | (0.71-3.76) | 0.252 |
| Hgb | 41 | 18 | 0 | 0.75 | (0.58-0.98) | 0.032 |
| Neut | 41 | 18 | 0 | 0.99 | (0.97-1.00) | 0.024 |
| ANC | 41 | 18 | 0 | 1.01 | (0.98-1.05) | 0.405 |
| BM Blast | 41 | 18 | 0 | 1.01 | (0.90-1.12) | 0.920 |
| NUM MUT | 41 | 18 | 0 | 1.00 | (0.69-1.44) | 1.000 |
| Gender |  |  |  |  |  |  |
| Female | 8 | 3 | 0 |  |  |  |
| Male | 33 | 15 | 0 | 1.13 | (0.36-3.58) | 0.837 |
| TMDS |  |  |  |  |  |  |
| No | 34 | 16 | 0 |  |  |  |
| Yes | 7 | 2 | 0 | 1.50 | (0.43-5.24) | 0.529 |
| CMML |  |  |  |  |  |  |
| No | 30 | 12 | 0 |  |  |  |
| Yes | 11 | 6 | 0 | 1.43 | (0.61-3.34) | 0.414 |
| Highrisk |  |  |  |  |  |  |
| No | 18 | 9 | 0 |  |  |  |
| Yes | 23 | 9 | 0 | 0.80 | (0.32-2.01) | 0.639 |
| PS |  |  |  |  |  |  |
| 0 | 16 | 7 | 0 |  |  |  |
| 1 | 21 | 10 | 0 | 8.38 | (2.02-34.74) | 0.003 |
| 2-3 | 4 | 1 | 0 | 1.57 | (0.57-4.31) | 0.379 |
| CG Complex |  |  |  |  |  |  |
| No | 29 | 13 | 0 |  |  |  |
| Yes | 11 | 4 | 0 | 1.40 | (0.40-4.87) | 0.602 |
| Diploid |  |  |  |  |  |  |
| No | 29 | 12 | 0 |  |  |  |
| Yes | 11 | 5 | 0 | 0.32 | (0.11-0.99) | 0.047 |
| IPSS-R CG |  |  |  |  |  |  |
| 0 | 2 | 1 | 0 |  |  |  |
| 1 | 15 | 7 | 0 | 0.23 | (0.09-0.62) | 0.004 |
| 2 | 12 | 5 | 0 | 0.46 | (0.14-1.46) | 0.187 |
| 3 | 1 | 1 | 0 | 14.53 | (2.32-91.05) | 0.004 |
| 4 | 10 | 3 | 0 | 0.36 | (0.08-1.64) | 0.184 |
| Plt<100 |  |  |  |  |  |  |
| No | 12 | 5 | 0 |  |  |  |
| Yes | 29 | 13 | 0 | 0.69 | (0.15-3.26) | 0.639 |
| Plt<50 |  |  |  |  |  |  |
| No | 25 | 11 | 0 |  |  |  |
| Yes | 16 | 7 | 0 | 0.71 | (0.30-1.71) | 0.449 |
| ASXL1 |  |  |  |  |  |  |
| No | 33 | 16 | 0 |  |  |  |
| Yes | 8 | 2 | 0 | 0.15 | (0.02-1.11) | 0.063 |
| RUNX1 |  |  |  |  |  |  |
| No | 35 | 16 | 0 |  |  |  |
| Yes | 6 | 2 | 0 | 0.69 | (0.19-2.47) | 0.570 |
| TET2 |  |  |  |  |  |  |
| No | 31 | 13 | 0 |  |  |  |
| Yes | 10 | 5 | 0 | 2.30 | (0.96-5.48) | 0.060 |
| TP53 |  |  |  |  |  |  |
| No | 33 | 15 | 0 |  |  |  |
| Yes | 8 | 3 | 0 | 1.76 | (0.54-5.79) | 0.350 |
| Mutation 1 or more |  |  |  |  |  |  |
| No | 7 | 3 | 0 |  |  |  |
| Yes | 34 | 15 | 0 | 1.04 | (0.31-3.44) | 0.953 |
| Mutation 2 or more |  |  |  |  |  |  |
| No | 20 | 7 | 0 |  |  |  |
| Yes | 21 | 11 | 0 | 1.31 | (0.51-3.37) | 0.576 |
| Mutation 3 or more |  |  |  |  |  |  |
| No | 25 | 11 | 0 |  |  |  |
| Yes | 16 | 7 | 0 | 3.34 | (1.19-9.36) | 0.022 |
| Mutation 4 or more |  |  |  |  |  |  |
| No | 36 | 16 | 0 |  |  |  |
| Yes | 5 | 2 | 0 | 2.23 | (0.94-5.26) | 0.068 |
| BCOR |  |  |  |  |  |  |
| No | 37 | 16 | 0 |  |  |  |
| Yes | 4 | 2 | 0 | 1.82 | (0.80-4.16) | 0.154 |
| SRSF2 |  |  |  |  |  |  |
| No | 33 | 15 | 0 |  |  |  |
| Yes | 8 | 3 | 0 | 1.58 | (0.44-5.63) | 0.478 |
| STAG2 |  |  |  |  |  |  |
| No | 38 | 16 | 0 |  |  |  |
| Yes | 3 | 2 | 0 | 3.38 | (0.88-12.93) | 0.075 |
| U2AF1 |  |  |  |  |  |  |
| No | 38 | 17 | 0 |  |  |  |
| Yes | 3 | 1 | 0 | 2.07 | (0.88-4.86) | 0.097 |
| ZRSR2 |  |  |  |  |  |  |
| No | 38 | 17 | 0 |  |  |  |
| Yes | 3 | 1 | 0 | 2.48 | (0.96-6.41) | 0.060 |
| Splicing |  |  |  |  |  |  |
| No | 27 | 13 | 0 |  |  |  |
| Yes | 14 | 5 | 0 | 2.03 | (0.70-5.88) | 0.193 |
| DNA methylation |  |  |  |  |  |  |
| No | 28 | 10 | 0 |  |  |  |
| Yes | 13 | 8 | 0 | 2.70 | (1.01-7.19) | 0.048 |
| Cohesins |  |  |  |  |  |  |
| No | 38 | 16 | 0 |  |  |  |
| Yes | 3 | 2 | 0 | 3.38 | (0.88-12.93) | 0.075 |
| Chromatine |  |  |  |  |  |  |
| No | 33 | 16 | 0 |  |  |  |
| Yes | 8 | 2 | 0 | 0.15 | (0.02-1.11) | 0.063 |
| Transcription Factors |  |  |  |  |  |  |
| No | 28 | 12 | 0 |  |  |  |
| Yes | 13 | 6 | 0 | 0.92 | (0.39-2.16) | 0.855 |
| Signaling |  |  |  |  |  |  |
| No | 36 | 15 | 0 |  |  |  |
| Yes | 5 | 3 | 0 | 2.60 | (0.82-8.24) | 0.104 |
| DNA damage |  |  |  |  |  |  |
| No | 32 | 15 | 0 |  |  |  |
| Yes | 9 | 3 | 0 | 1.72 | (0.52-5.64) | 0.374 |
| Other |  |  |  |  |  |  |
| No | 39 | 18 | 0 |  |  |  |
| Yes | 2 | 0 | 0 | - | - | - |
