# Supplementary material for: Impact of the number of mutations in survival and response outcomes to hypomethylating agents in patients with myelodysplastic syndromes or myelodysplastic/myeloproliferative neoplasms
Source: Oncotarget. 2018 Jan 3;9(11):9714–27. doi: 10.18632/oncotarget.23882 (PMC5839396; doi:10.18632/oncotarget.23882)
Supplement: Supplementary file 10 [file oncotarget-09-9714-s010.docx]

**Supplementary Table 11. Characteristics of patients in the additional cohort.**

| **Variable** | **Number**  **[Range]/(%)** |
| --- | --- |
| Age | 68 [25-91] |
| Female | 129 (31.2) |
| WBC (x10^9^/L) | 6.8 [0.5-183.4] |
| ANC (x10^9^/L) | 2.9 [0-80.69] |
| Hb (g/dL) | 10.2 [5-16.5] |
| PLT (x10^9^/L) | 131 [3-1554] |
| Bone marrow blasts (%) | 5 [0-19] |
| WHO Classification |  |
| MDS-SLD | 6 (1.5) |
| MDS-RS | 38 (9.2) |
| MDS-MLD | 123 (29.8) |
| MDS-EB | 136 (32.9) |
| MDS-U | 23 (5.6) |
| MDS/MPN | 15 (3.6) |
| MDS with del(5q) | 9 (2.2) |
| CMML | 63 (2.2) |
| IPSS-R Cytogenetics |  |
| Very good | 9 (2.2) |
| Good | 226 (54.7) |
| Intermediate | 75 (18.2) |
| Poor | 23 (5.6) |
| Very poor | 79 (19.1) |
| IPSS |  |
| Low | 101 (24.5) |
| Int-1 | 160 (38.7) |
| Int-2 | 122 (29.5) |
| High | 30 (7.3) |
| IPSS-R |  |
| Very low | 42 (10.2) |
| Low | 117 (28.3) |
| Intermediate | 84 (20.3) |
| High | 99 (24) |
| Very high | 71 (17.2) |
| Mutated Genes |  |
| ABL1 | 2 (0.5) |
| ASXL1 | 58 (14) |
| BRAF | 4 (1) |
| DNMT3A | 41 (9.9) |
| EGFR | 2 (0.5) |
| EZH2 | 15 (3.6) |
| FLT3 | 4 (1) |
| GATA1 | 3 (0.7) |
| GATA2 | 3 (0.7) |
| HRAS | 0 (0) |
| IDH1 | 13 (3.1) |
| IDH2 | 25 (6.1) |
| IKFZ2 | 0 (0) |
| JAK2 | 12 (2.9) |
| KIT | 1 (0..2) |
| KRAS | 6 (1.5) |
| MDM2 | 0 (0) |
| MLL | 2 (0.5) |
| MPL | 3 (0.7) |
| MYD88 | 0 (0) |
| NOTCH1 | 9 (2.2) |
| NPM1 | 16 (3.9) |
| NRAS | 18 (4.4) |
| PTPN11 | 6 (1.5) |
| RUNX1 | 46 (11.1) |
| TET2 | 77 (18.6) |
| TP53 | 66 (16) |
| WT1 | 2 (0.5) |
